# Isolation, Bioactivity, and Molecular Docking of a Rare Gastrodin Isocitrate and Diverse Parishin Derivatives from *Gastrodia elata* Blume

**DOI:** 10.1021/acsomega.4c00436

**Published:** 2024-03-14

**Authors:** Jie Zhou, Jia-Qian Chen, Shilin Gong, Yu-Juan Ban, Li Zhang, Ying Liu, Jian-Lin Wu, Na Li

**Affiliations:** † State Key Laboratory of Quality Research in Chinese Medicine, Macau Institute for Applied Research in Medicine and Health, Macau University of Science and Technology, Taipa 999078 SAR, China; ‡ School of Basic Medicinal Sciences and Nursing, Chengdu University, Chengdu 610106, PR China

## Abstract

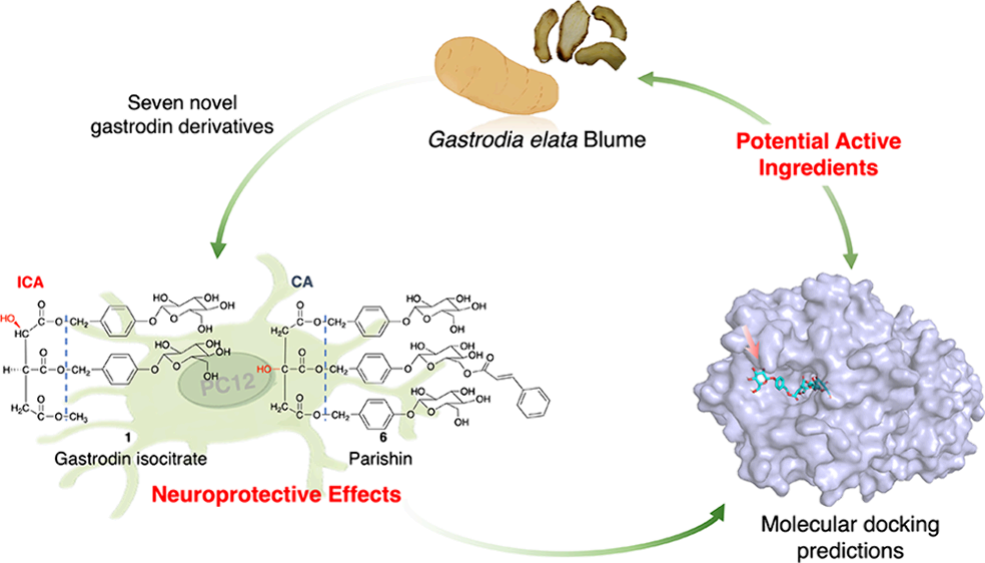

*Gastrodia elata* Blume (*G. elata*) is a well-known medicine food homology plant widely used in treating neurological disorders such as Alzheimer’s disease (AD). Here, undiscovered gastrodin derivatives were systematically studied. Seven novel gastrodin derivatives (**1**–**7**), including a unique gastrodin isocitrate (**1**) and six differently substituted parishin derivatives (**2**–**7**), were isolated. Structural identification was mainly based on 1D and 2D NMR data, high-resolution ESI-MS data, and HPLC analysis. Notably, the stereochemistry of **1** was further elucidated by ECD calculations. Compounds **1** and **6** showed neuroprotective effects on the H_2_O_2_-induced PC12 cell injury model. Molecular docking analysis exhibited that **1** and **6** had good affinities with three popular AD-related targets. These findings not only enriched the chemical diversity but also revealed potential active components in *G. elata*.

## 1 Introduction

Alzheimer’s disease (AD) is a common neurodegenerative disorder characterized by a gradual decline in cognitive function, memory impairment, and behavioral abnormalities.^1,2^ As the aging population increases worldwide, AD has emerged as a crucial public health issue that poses a significant threat to the health and life of the elderly.^3^ Unfortunately, there are no genuinely effective interventions available to treat, reverse, or delay the progression of AD. Therefore, the need for innovative bioactive molecules focused on the treatment or targeted prevention of AD has become increasingly urgent. Recently, an increasing number of people have adopted the concept of “food as medicine” in disease prevention, treatment, and health care.^4^ Numerous epidemiological studies have indicated an association between consuming foods that are rich in polyphenols and preventing chronic diseases.^5^ Phenolics from dietary or plant sources have been shown to have neuroprotective effects, particularly against AD.^6−8^ The promising medicine food homology plant *Gastrodia elata* Blume (*G. elata*) is an excellent example.^9,10^ Modern chemical analysis and pharmacological studies have shown that the active constituents of *G. elata* are mainly gastrodin (a phenolic glycoside) and its derivatives.^11−14^ This has stimulated further exploration of the potentially active gastrodin derivatives in *G. elata*.

In this study, we performed a systematic and comprehensive investigation of gastrodin derivatives with potential activity in *G. elata*. First, gastrodin derivatives were isolated and purified by silica gel column chromatography (CC), microporous resin chromatography, and high-performance liquid chromatography (HPLC). Then, their structures were elucidated by NMR and HR-ESI-MS data, derivatization-HPLC analyses, and electronic circular dichroism (ECD) calculations. Next, a reactive oxygen species (ROS)-induced neuronal injury model, using H_2_O_2_ to induce rapid contraction and apoptosis in PC12 cells, was applied for neuroprotective activity evaluation *in vitro*.^15^ Finally, molecular docking was used to predict the binding energies of the target proteins. Herein, we report the structure elucidation, neuroprotection, and molecular docking of gastrodin derivatives.

## 2 Experimental Section

### 2.1 Materials

AR-grade *n*-hexane, ethyl acetate, *n*-butanol, methanol (MeOH), 95% ethanol, dichloromethane (DCM), and HPLC-grade MeOH and acetonitrile (ACN) were purchased from Anaqua Chemicals Supply (ACS, USA). d-(+)-Glucose (≥99.5%), pyridine, the NMR solvent methanol-*d*_4_ (MeOD, 99.8 atom % D), and Trolox (97%) were provided by Sigma–Aldrich (USA). The solid phases used for the separation were silica gel (75–150 mesh; Grace, USA), Diaion HP-20 macroporous resin (>250 μm; Mitsubishi, Japan), and octadecylsilyl (ODS; 35–70 μm; Grace, USA). Sodium hydroxide (NaOH, 96% flaky solid), hydrochloric acid (HCl, 37% solution in water), l-(−)-glucose (≥99.5%), l-cysteine methyl ester hydrochloride (98%), and *o*-toryl isothiocyanate (98%) were obtained from Macklin (Shanghai, China).

### 2.2 General Experimental Procedures

HR-ESI-MS data were measured using an Agilent 6230 accurate mass time-of-flight mass spectrometer with an Agilent Eclipse Plus C18 RRHD column (2.1 × 100 mm, 1.8 μm). All semipreparative HPLC separations were equipped with an Agilent 1100 multiple-wavelength absorbance detector with a mobile phase using the H_2_O/ACN system and detected on a BEH phenyl column (250 × 10 mm, 5 μm, Waters) at 210 and 254 nm. All NMR data were collected by Bruker Ascend 600 NMR using methanol-*d*_4_ as a solvent. A Rudolph Research Analytical Autopol I automatic polarimeter was used for recording optical rotations, a Beckman UV–Vis Spectrometer (DU-800) was used for acquiring UV spectra, and an Agilent Cary 600 series Fourier Transform Infrared (FT-IR) Spectrometer was used for obtaining IR spectra (KBr).

### 2.3 Plant Material

The rhizome of *G. elata* was purchased from Guangyuan City of Sichuan Province, China, and authenticated by one of the authors, Dr. Ying Liu.

### 2.4 Extraction and Isolation

*G. elata* powders (4.4 kg) were extracted with 95% ethanol (3 × 6 L) by heating with reflux. The combined ethanol extract (539.5 g) was evaporated under a vacuum, dissolved in H_2_O, and then sequentially engaged with *n*-hexane, ethyl acetate, and *n*-butanol. Nine fractions (Fr.1–Fr.9) were obtained from the *n*-butanol extract (86.21 g) by silica gel CC with DCM/MeOH (1:0–0:1, v/v) as the eluent. Subfractions Fr.5–Fr.7 were selected for further separation. Fr.5 and Fr.7 were repeatedly eluted with DCM/MeOH (1:0–0:1, v/v) via silica gel CC to afford Fr.5–1–Fr.5–9 and Fr.7–1–Fr.7–8, respectively. Then, Fr.5–8 and Fr.6 were eluted stepwise using a Diaion HP-20 column with H_2_O/MeOH (1:0–0:1, v/v) to give Fr.5–8–1–Fr.5–8–11 and Fr.6–1–Fr.6–12, respectively. Fr.5–8–10 was isolated by semipreparative HPLC (22%–30% ACN-H_2_O, 28–52 min) to obtain subfraction Fr.5–8–10–3 (t_R_ = 35–40 min). Further purification of Fr.5–8–10–3 was performed using 21% ACN-H_2_O, which afforded compounds **4** (2.1 mg, t_R_ = 55.0 min) and **5** (2.2 mg, t_R_ = 52.2 min). Additionally, Fr.6–9 was purified by semipreparative HPLC and eluted with 15% ACN to obtain compound **1** (6.2 mg, t_R_ = 18.2 min). Fr.6–11 was isolated by semipreparative HPLC with 20% ACN to yield compounds **2** (8.8 mg, t_R_ = 28.5 min) and **3** (15.9 mg, t_R_ = 32.4 min). Finally, Fr.6–12 was eluted with 20% ACN to afford compounds **6** (5.3 mg, t_R_ = 50.1 min) and **7** (2.2 mg, t_R_ = 52.7 min).

1. Light-yellow amorphous powder, [α]^22.5^_D_ – 37.4 (*c* 0.05, MeOH); UV (MeOH) λ_max_ (log ε) 223 (4.29), 271 (3.07), 277 (2.96); IR (KBr) ν_*max*_: 3356, 2924, 2855, 1736, 1612, 1512, 1373, 1227, 1072 cm^–1^; ECD (MeOH) λ_max_ (Δε): 200.5 (+0.33), 221.4 (−0.41); HR-ESI-MS (neg.): *m*/*z* 741.2274 [M – H]^−^ (calcd for C_33_H_42_O_19_, 741.2248).
2. Yellow amorphous powder, [α]^22.5^_D_ – 53.6 (*c* 0.05, MeOH); UV (MeOH) λ_max_ (log ε) 223 (4.51), 271 (3.62), 277 (3.60); IR (KBr) ν_*max*_: 3364, 2924, 2855, 1736, 1612, 1512, 1366, 1227, 1072 cm^–1^; HR-ESI-MS (neg.): *m*/*z* 1101.3504 [M – H]^−^ (calcd for C_52_H_62_O_26_, 1101.3457).
3. Yellow amorphous powder, [α]^22.5^_D_ – 54.7 (*c* 0.05, MeOH); UV (MeOH) λ_max_ (log ε) 223 (4.56), 270 (3.60), 277 (3.59); IR (KBr) ν_*max*_: 3364, 2924, 2855, 1736, 1612, 1512, 1373, 1227, 1072 cm^–1^; HR-ESI-MS (neg.): *m*/*z* 1101.3499 [M – H]^−^ (calcd for C_52_H_62_O_26_, 1101.3457).
4. Light-yellow amorphous powder, [α]^22.5^_D_ – 40.3 (*c* 0.05, MeOH); UV (MeOH) λ_max_ (log ε) 223 (4.53), 272(3.88), 278(3.96), 312 (3.26); IR (KBr) ν_*max*_: 3372, 2924, 2855, 1736, 1512, 1373, 1227, 1072 cm^–1^; HR-ESI-MS (neg.): *m*/*z* 1141.3435 [M – H]^−^ (calcd for C_54_H_62_O_27_, 1141.3406).
5. Light-yellow amorphous powder, [α]^22.5^_D_ – 53.9 (*c* 0.05, MeOH); UV (MeOH) λ_max_ (log ε) 222 (7.06), 272(4.24), 277 (4.24); IR (KBr) ν_*max*_: 3395, 2927, 2855, 1743, 1512, 1373, 1227, 1072 cm^–1^; HR-ESI-MS (neg.): *m*/*z* 1125.3506 [M – H]^−^ (calcd for C_54_H_62_O_26_, 1125.3457).
6. Light-yellow amorphous powder, [α]^22.5^_D_ – 41.7 (*c* 0.05, MeOH); UV (MeOH) λ_max_ (log ε) 222 (6.44), 271(4.29), 278 (4.22); IR (KBr) ν_*max*_: 3356, 2924, 2855, 1736, 1612, 1512, 1366, 1227, 1072 cm^–1^; HR-ESI-MS (neg.): *m*/*z* 1125.3486 [M – H]^−^ (calcd for C_54_H_62_O_26_, 1125.3457).

### 2.5 Derivatization-HPLC Analysis

The sugar units of new compounds **1**–**7** were determined according to the reported method.^16^ Each compound (0.5 mg) was hydrolyzed in 0.5 M HCl at 95 °C for 2 h followed by neutralization with 0.5 M NaOH. After complete drying in vacuo, the residue was reacted with l-cysteine methyl ester in pyridine (0.1 mL; 5 mg/mL) for 1 h at 60 °C. Next, *o*-toryl isothiocyanate dissolved in pyridine (0.1 mL; 5 mg/mL) was added and reacted for 1 h at the same temperature. Derivatization of d-(+)-glucose and l-(−)-glucose was carried out in the same way, while the reaction mixture without glucose was used as a blank control. Direct HPLC analysis of all reaction mixtures was conducted using a VisionHT C18 HL analytical column (250 × 4.6 mm, 5 μm; Grace) at 250 nm, and the mobile phase was 0.1% FA-containing 25% ACN. The flow rate and running time were 0.8 mL/min and 14 min, respectively. The absolute configurations of the sugar units in the new compounds were determined by a comparison of the t_R_ values with those of the authentic standards.

### 2.6 ECD Calculations

The conformational analysis of compound **1** was conducted as follows. First, the 10 lowest energy state conformations were provided by the MMFF94S force field calculation in Sybyl-X 2.0 (5.0 kcal/mol). Second, the possible conformers were calculated and ranked using polarizable conductor calculation model by ORCA5.0.1 software at the B3LYP-D3(BJ)/6-31G* level.^17^ Subsequently, the parameters for the first 60 excited states of the main conformations were calculated at the PBE0/def2-TZVP level using time-dependent density functional theory (TD-DFT).^18^ Finally, the ECD curves of possible isomers were simulated, and the absolute configuration of **1** was determined by a comparison of the experimental ECD curve to the simulated curves.

### 2.7 Cell Culture

To investigate the neuroprotective effects of the isolated compounds, a H_2_O_2_-induced injury model was constructed in PC12 cells (Shanghai Institute of Biochemistry and Cell Biology, China). Cells were cultured in Dulbecco’s modified Eagle’s medium (Gibco, USA) supplemented with 10% fetal bovine serum (Gibco, USA), 5% horse serum (Gibco, USA), and 1% penicillin–streptomycin, then inoculated into 96-well cell culture plates at a density of 1.0 × 10^4^ per well, and incubated at 37 °C with 5% CO_2_ for 24 h. The culture media of the control group and model group were replaced with 100 μL of a new culture media. Afterward, 100 μL of the test compounds (5 μM, 10 μM, and 20 μM) was added to the experimental group and incubated at the same temperature and CO_2_ conditions for 4 h. After that, H_2_O_2_ (10 μL, final concentration of 600 μM) was added to the model group, experimental group, and positive control (Trolox), and the culture was continued for 24 h. Finally, Cell Counting Kit-8 solution (Dojindo, Japan) was added to each well, and incubation was continued at 37 °C for 1 h. Immediately afterward, the optical density (OD) values were recorded with an enzyme labeling instrument at 450 nm.

### 2.8 Molecular Docking

To predict the potential of compounds discovered from *G. elata*, their binding energies with kelch-like ECH-associated protein 1 (Keap1)-nuclear factor erythroid 2-related factor 2 (Nrf2), beta-secretase 1 (BACE1), and apolipoprotein E4 (APOE4) were investigated. The 3D structures of Keap1-Nrf2 (PDB entry 4L7B), BACE1 (PDB entry 1M4H), and APOE4 (PDB entry 1B68) were obtained from the Protein Data Bank Database. Polar hydrogens were added, and all water molecules were removed from the protein. The structures of all of the compounds were manually constructed, and the predicted binding mode was defined as the conformation with the lowest free energy. AutoDock vina docking 1.1.2 software was used to dock the 3D minimum-energy ligands and proteins.

## 3 Results and Discussion

### 3.1 Structure Elucidation

**Figure 1:**
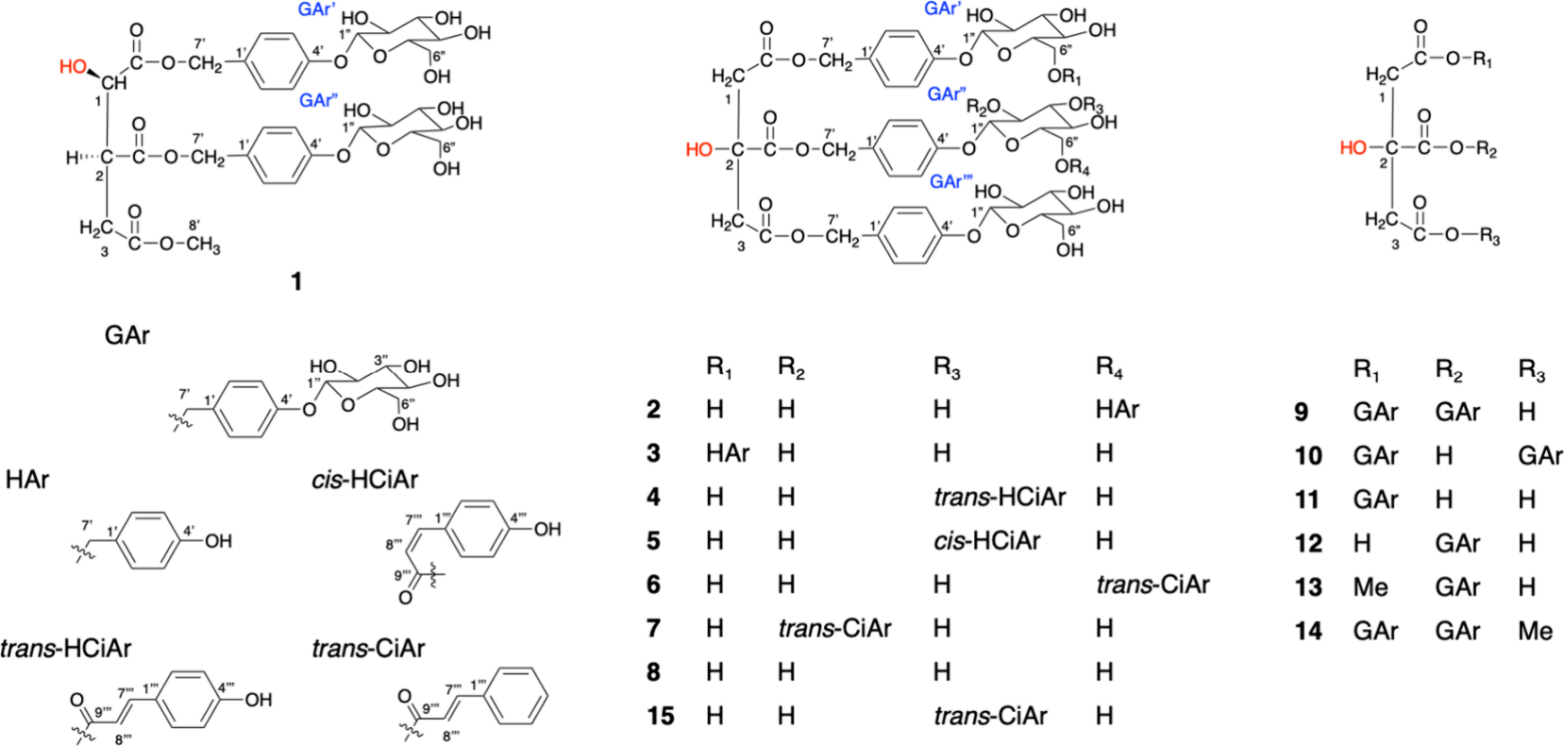
Chemical structures of compounds **1**–**15**.

Seven novel gastrodin derivatives (**1**–**7**) were successfully isolated (Figure 1), especially the rare gastrodin isocitrate (**1**), which has an isocitric acid (ICA) backbone and was found in nature for the first time. New parishin etherified derivatives (**2**–**3**), esterified derivatives (**4**–**7**), and known parishins (**8**–**15**) with citric acid (CA) as the backbone were also identified.

#### 3.1.1 Gastrodin Isocitrate

**Figure 2:**
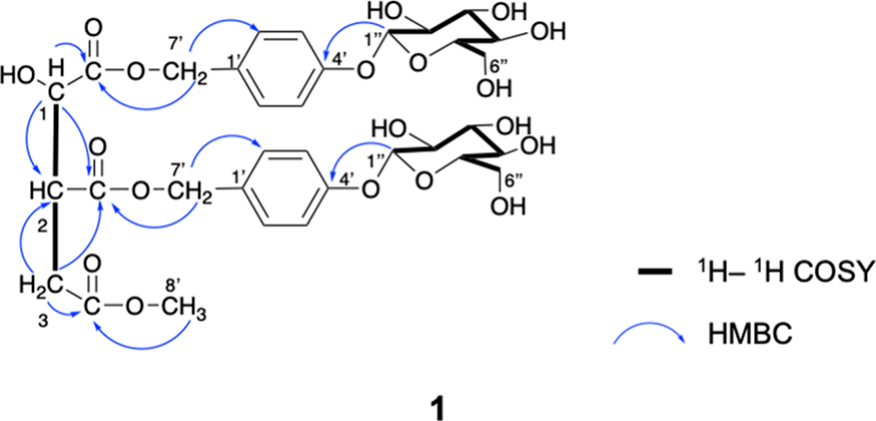
Key ^1^H–^1^H COSY and HMBC correlations of compound **1**.

**Figure 3:**
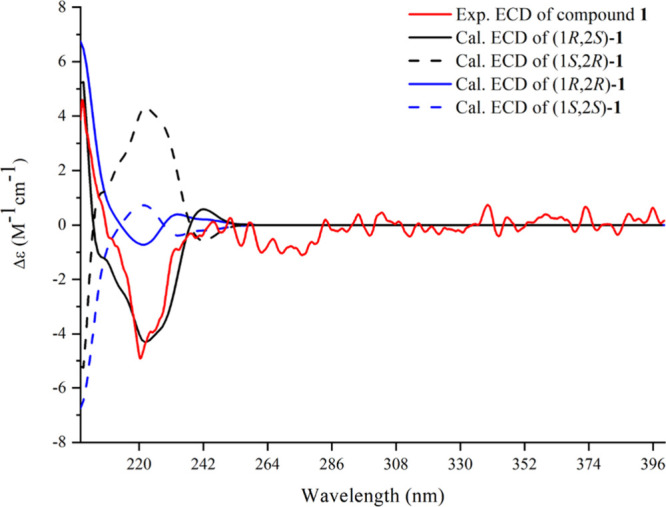
Experimental and calculated ECD spectra of compound **1**.

Compound **1** was isolated as a light yellow, amorphous powder. The molecular formula was assigned as C_33_H_42_O_19_ from HR-ESI-MS (Figure S1) at *m*/*z* 741.2274 (calcd 741.2248). Its IR spectrum showed the presence of hydroxyl groups (3356 cm^–1^), carboxylic groups (1736 cm^–1^), and aromatic rings (1612 and 1512 cm^–1^). As shown in Table 1, the ICA was deduced from the ^1^H and ^13^C chemical shifts, coupling constants (*J*), HSQC, and HMBC associations. The connection sequences were determined by ^1^H–^1^H COSY correlations among H-1 (δ 4.44), H-2 (δ 3.44), and H-3 (δ 2.60/2.80), the HMBC correlations from H-1 to δ_C_ 173.6 and δ_C_ 172.2 and from H-3 to δ_C_ 46.6 (C-2) and δ_C_ 174.0 (Figures S5–S7). There were two similar sets of signals detected at δ_H_ 7.23 (H-2′, 6′), 7.07 (H-3′, 5′), and 4.95 (H-7′) as well as δ_H_ 7.21 (H-2′, 6′), 7.06 (H-3′, 5′), and 4.95 (H-7′), which corresponded to AA’BB’-type aromatic and methylene groups belonging to two *p*-hydroxybenzyl alcohol residues (HArs). Furthermore, two sugar anomeric carbons were detected at δ_C_ 102.3 (two carbons) in the ^13^C NMR spectrum, corresponding to proton signals at δ_H_ 4.88 (*J* = 7.5 Hz) and 4.88 (*J* = 7.4 Hz) in HSQC, respectively. Two glucose moieties were identified by ^1^H–^1^H COSY, TOCSY, HSQC, and HMBC correlations with anomeric protons (Figure S5–S8), and the β-configuration was revealed according to the large coupling constants (*J* ∼ 7.5 Hz) of two anomeric protons.^19,20^ The absolute configurations of the glucoses were further determined by using HPLC analysis after thiocarbamoyl-thiazolidine derivatization. Only one peak was observed at 10.954 min in compound **1**, which was close to that of authentic d-(+)-glucose (10.839 min) (Figure S13); thus, the absolute configuration of glucose should be the d-form. Moreover, each anomeric proton (H-1″) was related to C-4’ of each HAr in the HMBC spectrum to form the gastrodin residue (GAr) (Figure 2). H-7′ of each GAr was found to be severally linked to the ICA unit via the HMBC correlations from δ_H_ 4.95 (GAr′) to δ_C_ 173.6 and from δ_H_ 4.95 (GAr″) to δ_C_ 172.2. Additionally, the carbon at δ_C_ 52.3 (C-8’) was assigned to a methoxy group in the ICA unit according to chemical shifts and HMBC correlations. To further determine the absolute configuration of **1**, the theoretical ECD data were calculated by using TD-DFT (Tables S1–S11). As shown in Figure 3, the experimental ECD curve of **1** exhibited a positive Cotton effect (CE) at 200.5 nm and a strong negative CE at 221.4 nm, which was similar to the calculated ECD curve of (1*R*,2*S*)-**1**. Ultimately, **1** was identified as (1*R*,2*S*)-di-[4-*O*-(β-d-glucopyranosyl)benzyl]-3-methyl isocitrate.

**Table 1:** ^1^H (600 MHz) and ^13^C NMR (150 MHz) Data of Compound **1** in MeOD (δ in ppm, *J* in Hz)

	**1**	
position	δ_H_, mult (*J*)	δ_C_
ICA		
1	4.44, 1H, d (3.4)	71.9
2	3.44, 1H, overlap	46.6
3	2.60, 1H, dd (17.0, 4.9)	32.9
	2.80, 1H, dd (17.0, 9.9)	
1-COO		173.6
2-COO		172.2
3-COO		174.0
GAr′		
1’		130.9
2′, 6′	7.23, 2H, d (8.7)	131.2
3′, 5′	7.07, 2H, d (8.7)	117.8
4′		159.2
7′	4.95, 2H, overlap	67.7
1″	4.88, 1H, d (7.5)	102.3
2″	3.46, overlap	74.9
3″	3.45, overlap	77.9
4″	3.38, overlap	71.4
5″	3.44, overlap	78.1
6″	3.70, 1H, dd (12.0, 5.4)	62.5
	3.88, 1H, dd (12.0, 2.2)	
GAr″		
1′		130.8
2′, 6′	7.21, 2H, d (8.7)	131.1
3′, 5′	7.06, 2H, d (8.7)	117.8
4′		159.2
7′	4.95, 2H, overlap	67.9
1″	4.88, 1H, d (7.4)	102.3
2″	3.46, overlap	74.9
3″	3.45, overlap	77.9
4″	3.38, overlap	71.4
5″	3.44, overlap	78.1
6″	3.69, 1H, dd (12.0, 5.3)	62.5
	3.87, 1H, dd (12.0, 2.2)	
8′	3.63, 3H, s	52.3

#### 3.1.2 Parishin Derivatives

**Figure 4:**
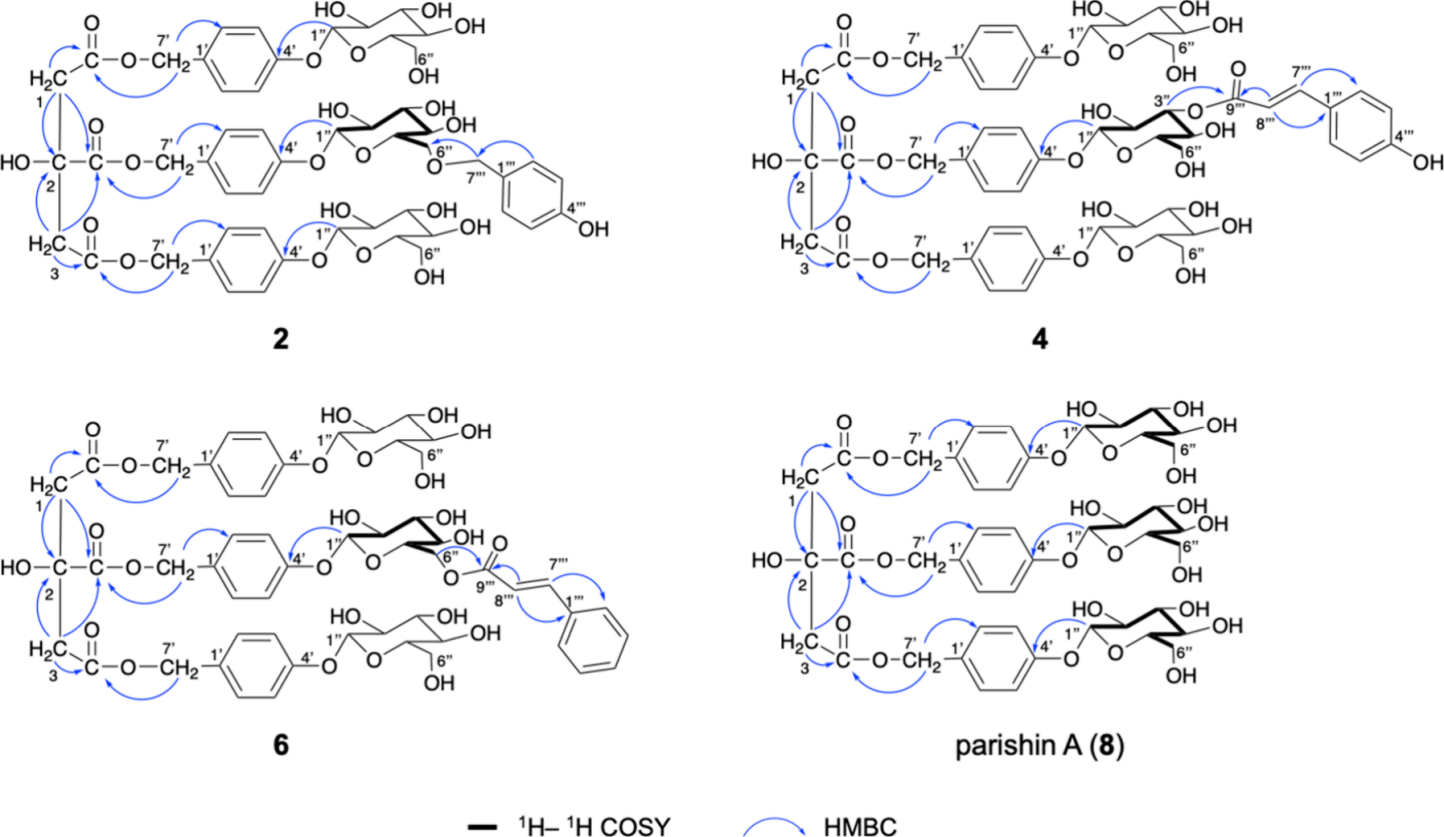
Key ^1^H–^1^H COSY and HMBC correlations of compounds **2**, **4**, **6**, and **8**.

Compound **2**, a yellow amorphous powder with the molecular formula C_52_H_62_O_26_ (*m*/*z* 1101.3504, [M – H]^−^, Figure S14), was found to be a HAr-containing derivative of parishin A (compound **8**) based on the difference in the molecular weight of C_7_H_6_O and the variations in the NMR spectra. As demonstrated in Figure 4 and Tables 2 and 3, similar parishin A unit-related signals were detected for a CA unit connected to three GArs via the HMBC correlations from δ_H_ 4.97 (H-7′, GAr′) to δ_C_ 170.9, from δ_H_ 4.94 (H-7′, GAr″) to δ_C_ 174.3, and from δ_H_ 4.97 (H-7′, GAr‴) to δ_C_ 170.9, respectively. In contrast, the presence of extra HAr was indicated by the presence of AA’BB’-type aromatic signals at δ_H_ 6.72 (*J* = 8.4 Hz) and 7.12 (*J* = 8.4 Hz) and methylenoxy signals at δ_H_ 4.42 (Table 2). The downfield shift of C-6″ (Δδ_C_ + 8.0) in one GAr (Table 3) correlated with H-6″ (δ_H_ 3.59/3.83) in HSQC was observed (Figure S19), and the HMBC correlation of C-6″ with H-7‴ of HAr suggested that this GAr was substituted by HAr at C-6″ (Figure S20). Symmetrical structural information similar to that of parishin A was embodied in two doublets of methylene groups at δ_H_ 2.77 (2H, d, *J* = 15.3 Hz) and 2.94 (2H, d, *J* = 15.3 Hz) and two carbons at δ_C_ 170.9 and 174.3 in the CA unit, which indicated that the etherified GAr was GAr″. The absolute configuration of the sugar was determined to be β-d-glucose following the same method as that used for **1** (Figure S13). Therefore, **2** was determined to be 1,3-di-[4-*O*-(β-d-glucopyranosyl)benzyl]-2-{4-*O*-[6-*O*-(4-hydroxybenzyl)-β-d-glucopyranosyl]benzyl} citrate.

**Table 2:** ^1^H NMR (600 MHz) Data of Compounds **2**–**7** and Parishin A (**8**) in MeOD (δ in ppm, *J* in Hz)

position	**2**	**3**	**4**	**5**	**6**	**7**	parishin A **(8)**
CA							
1	2.78, d (15.3)	2.78, d (15.3)	2.78, d (15.4)	2.78, d (15.4)	2.73, d (15.4)	2.75, d (15.2)	2.78, d (15.5)
	2.94, d (15.3)	2.95, d (15.3)	2.96, d (15.4)	2.96, d (15.4)	2.88, d (15.4)	2.92, d (15.2)	2.95, d (15.5)
3	2.78, d (15.3)	2.79, d (15.3)	2.78, d (15.4)	2.78, d (15.4)	2.73, d (15.4)	2.75, d (15.2)	2.78, d (15.5)
	2.94, d (15.3)	2.96, d (15.3)	2.96, d (15.4)	2.96, d (15.4)	2.88, d (15.4)	2.92, d (15.2)	2.95, d (15.5)
GAr′							
2′, 6′	7.24, d (8.5)	7.25, m	7.26, d (8.6)	7.27, d (8.5)	7.22, d (8.6)	7.22, d (8.7)	7.22, d (8.7)
3′, 5′	7.06, d (8.5)	7.06, d (8.7)	7.08, d (8.6)	7.08, d (8.5)	7.05, d (8.6)	7.06, d (8.7)	7.07, d (8.7)
7′	4.97, m	4.98, overlap	4.98, overlap	4.98, overlap	4.93, overlap	4.94, overlap	4.98, m
1″	4.88, overlap	4.87, d (7.4)	4.89, overlap	4.89, overlap	4.88, overlap	4.89, overlap	4.89, overlap
2″	3.46, overlap	3.46, overlap	3.46, overlap	3.46, overlap	3.46, overlap	3.46, overlap	3.46, overlap
3″	3.46, overlap	3.46, overlap	3.47, overlap	3.46, overlap	3.46, overlap	3.46, overlap	3.46, overlap
4″	3.39, overlap	3.37, overlap	3.39, overlap	3.39, overlap	3.40, overlap	3.40, overlap	3.39, overlap
5″	3.42, overlap	3.58, overlap	3.43, overlap	3.42, overlap	3.44, overlap	3.44, overlap	3.43, overlap
6″	3.69, dd (12.0, 5.2)	3.59, overlap	3.70, dd (12.1, 5.4)	3.70, dd (11.6, 4.8)	3.70, dd (12.1, 4.9)	3.70, dd (12.2, 5.4)	3.70, dd (12.0, 5.3)
	3.87, dd (12.0, 1.6)	3.83, d (9.3)	3.88, dd (12.1, 1.5)	3.88, d (11.6)	3.88, dd (12.1, 1.8)	3.87, dd (12.2, 1.5)	3.88, dd (12.0, 2.0)
GAr″							
2′, 6′	7.16, d (8.7)	7.15, d (8.5)	7.18, d (8.5)	7.17, d (8.4)	7.18, d (8.5)	7.16, d (8.7)	7.16, d (8.7)
3′, 5′	7.05, d (8.7)	7.03, d (8.5)	7.05, d (8.5)	7.05, d (8.4)	7.05, d (8.5)	6.96, d (8.7)	7.04, d (8.7)
7′	4.94, overlap	4.90, overlap	4.92, overlap	4.91, overlap	4.92, overlap	4.90, m	4.91, overlap
1″	4.86, overlap	4.87, d (7.6)	5.00, overlap	4.99, overlap	4.89, overlap	5.12, m	4.89, overlap
2″	3.46, overlap	3.46, overlap	3.66, overlap	3.61, overlap	3.48, overlap	5.10, m	3.46, overlap
3″	3.46, overlap	3.46, overlap	5.16, t (9.4)	5.14, t (9.3)	3.46, overlap	3.73, t (8.6)	3.46, overlap
4″	3.34, overlap	3.39, overlap	3.64, overlap	3.57, overlap	3.41, overlap	3.52, overlap	3.39, overlap
5″	3.58, overlap	3.43, overlap	3.55, overlap	3.55, overlap	3.74, overlap	3.53, overlap	3.43, overlap
6″	3.59, overlap	3.69, dd (12.0, 5.3)	3.74, dd (12.1, 5.3)	3.73, overlap	4.35, dd (11.8, 7.0)	3.75, dd (11.9, 5.1)	3.70, dd (12.0, 5.3)
	3.83, d (9.3)	3.87, d (12.0)	3.90, overlap	3.90, overlap	4.55, dd (11.8, 1.8)	3.93, dd (11.9, 1.5)	3.88, dd (12.0, 2.0)
GAr‴							
2′, 6′	7.24, d (8.5)	7.24. m	7.26, d (8.6)	7.27, d (8.5)	7.22, d (8.6)	7.22, d (8.7)	7.22, d (8.7)
3′, 5′	7.06, d (8.5)	7.08, d (8.53)	7.08, d (8.6)	7.08, d (8.5)	7.05, d (8.6)	7.06, d (8.7)	7.07, d (8.7)
7′	4.97, overlap	4.98, overlap	4.98, overlap	4.98, overlap	4.93, overlap	4.94, overlap	4.98, m
1″	4.89, overlap	4.87, d (7.4)	4.89, overlap	4.89, overlap	4.89 overlap	4.89, overlap	4.89, overlap
2″	3.46, overlap	3.46, overlap	3.46, overlap	3.46, overlap	3.46, overlap	3.46, overlap	3.46, overlap
3″	3.46, overlap	3.46, overlap	3.47, overlap	3.46, overlap	3.46, overlap	3.46, overlap	3.46, overlap
4″	3.39, overlap	3.39, overlap	3.39, overlap	3.39, overlap	3.40, overlap	3.40, overlap	3.39, overlap
5″	3.42, overlap	3.43, overlap	3.43, overlap	3.42, overlap	3.44, overlap	3.44, overlap	3.43, overlap
6″	3.69, dd (12.0, 5.2)	3.69, dd (12.2, 5.3)	3.70, dd (12.1, 5.4)	3.70, dd (11.6, 4.8)	3.70, dd (12.1, 4.9)	3.70, dd (12.2, 5.4)	3.70, dd (12.0, 5.3)
	3.87, dd (12.0, 1.6)	3.87, d (12.2)	3.88, dd (12.1, 1.5)	3.88, d (11.6)	3.88, dd (12.1, 1.8)	3.87, dd (12.2, 1.5)	3.88, dd (12.0, 2.0)
2‴, 6‴	7.12, d (8.4)	7.12, d (8.4)	7.49, d (8.5)	7.68, d (8.4)	7.59, d (8.5)	7.57, m	
3‴, 5‴	6.72, d (8.4)	6.73, d (8.4)	6.82, d (8.5)	6.75, d (8.4)	7.41, d (8.5)	7.39, overlap	
4‴					7.40, overlap	7.39, overlap	
7‴	4.42, m	4.43, m	7.69, d (16.1)	6.90, d (12.8)	7.68, d (16.2)	7.73, d (15.9)	
8‴			6.43, d (16.1)	5.88, d (12.8)	6.53, d (16.2)	6.55, d (15.9)	

**Table 3:** ^13^C NMR (150 MHz) Data of Compounds **2**–**7** and Parishin A (**8**) in MeOD (δ in ppm)

position	**2**	**3**	**4**	**5**	**6**	**7**	**8**
CA							
1	44.7	44.7	44.8	44.8	44.6	44.7	44.7
2	74.7	74.7	74.7	74.7	74.6	74.7	74.6
3	44.7	44.8	44.8	44.8	44.6	44.7	44.7
1-COO	170.9	170.9	170.9	170.9	170.9	170.9	170.9
2-COO	174.3	174.3	174.3	174.3	174.2	174.2	174.3
3-COO	170.9	170.9	170.9	170.9	170.9	170.9	170.9
GAr′							
1′	131.0	131.1	131.0	130.8	131.1	131.1	130.6
2′, 6′	131.1	131.1	131.1	131.1	131.0	131.1	131.0
3′, 5′	117.8	117.8	117.8	117.8	117.8	117.8	117.7
4′	159.1	159.1	159.2	159.2	159.1	159.2	159.1
7′	67.3	67.3	67.3	67.3	67.3	67.3	67.3
1″	102.3	102.3	102.3	102.3	102.3	102.3	102.3
2″	74.9	74.9	74.9	74.9	74.9	74.9	74.8
3″	77.9	78.0	77.9	77.9	77.9	78.0	77.9
4″	71.3	71.8	71.4	71.3	71.3	71.4	71.3
5″	78.1	77.1	78.2	78.1	78.1	78.1	78.1
6″	62.5	70.4	62.5	62.5	62.5	62.5	62.4
GAr″							
1′	130.7	130.7	130.8	130.8	131.8	131.1	130.6
2′, 6′	131.0	131.1	131.1	131.1	131.1	131.2	131.2
3′, 5′	117.9	117.9	117.7	117.8	117.7	117.7	117.8
4′	159.1	159.0	159.1	159.1	159.1	158.9	159.1
7′	68.2	68.2	68.2	68.2	68.2	68.1	68.1
1″	102.1	102.3	102.2	102.2	102.0	100.7	102.3
2″	74.9	74.9	73.3	73.2	74.8	75.3	74.8
3″	78.0	77.9	78.8	78.3	77.8	76.0	77.9
4″	71.8	71.4	69.6	69.5	71.8	71.5	71.3
5″	77.1	78.1	78.0	78.0	75.4	78.4	78.1
6″	70.4	62.5	62.2	62.2	64.9	62.5	62.4
GAr‴							
1′	131.0	131.1	131.1	130.8	131.1	131.1	130.6
2′, 6′	131.1	131.0	131.1	131.1	131.0	131.1	131.0
3′, 5′	117.8	117.9	117.9	117.8	117.8	117.8	117.7
4′	159.1	159.1	159.2	159.2	159.1	159.2	159.1
7′	67.3	67.3	67.3	67.3	67.3	67.3	67.3
1″	102.3	102.3	102.3	102.3	102.3	102.3	102.3
2″	74.9	74.9	74.9	74.9	74.9	74.9	74.8
3″	77.9	77.9	77.9	77.9	77.9	78.0	77.9
4″	71.3	71.3	71.4	71.3	71.3	71.3	71.3
5″	78.1	78.1	78.2	78.1	78.1	78.1	78.1
6″	62.5	62.5	62.5	62.5	62.5	62.5	62.4
1‴	130.4	130.4	127.2	127.6	135.7	135.7	
2‴, 6‴	130.7	130.7	131.2	133.7	129.4	129.3	
3‴, 5‴	116.1	116.1	116.1	115.8	130.2	130.1	
4‴	158.2	158.2	161.5	160.0	131.7	131.6	
7‴	74.3	74.3	146.7	144.9	146.6	146.9	
8‴			115.4	116.9	118.7	118.7	
9‴			169.1	168.1	168.3	167.8	

Compound **3** was obtained as a yellow amorphous powder and possessed the same molecular formula as **2** according to HR-ESI-MS (Figure S25). Its NMR spectra showed similar HAr-etherified GAr signals to those of **2**, as evidenced by one downfield shift of C-6″ (Δδ_C_ + 8.0) in one GAr (Table 3) and the association of ^1^H–^1^H COSY and HMBC (Figure S29–S31). However, in contrast to the CA unit signals of **2**, four doublets of methylene groups at δ_H_ 2.78 (1H, d, *J* = 15.3 Hz), 2.95 (1H, d, *J* = 15.3 Hz), 2.79 (1H, d, *J* = 15.3 Hz), and 2.96 (1H, d, *J* = 15.3 Hz) were observed, indicating structural asymmetry, which suggested that the etherified GAr was GAr′. Thus, **3** was identified as 2,3-di-[4-*O*-(β-d-glucopyranosyl)benzyl]-1-{4-*O*-[6-*O*-(4-hydroxybenzyl)-β-d-glucopyranosyl]benzyl} citrate.

Compound **4**, a light-yellow amorphous powder, had the molecular formula C_54_H_62_O_27_ (*m*/*z* 1141.3435, [M – H]^−^, Figure S36). As displayed in Table 3, in addition to the signals of the parishin A unit, **4** exhibited another *trans*-*p*-hydroxycinnamic acid residue (*trans*-HCiAr) according to the AA’BB’-type aromatic signals at δ_H_ 6.82 (*J* = 8.5 Hz, H-3‴, 5‴) and 7.49 (*J* = 8.5 Hz, H-2‴, 6‴), a pair of *trans* double bond signals at δ_H_ 7.69 (*J* = 16.1 Hz, H-7‴) and 6.43 (*J* = 16.1 Hz, H-8‴), and an ester carbonyl at δ_C_ 169.1 (C-9‴). Thus, **4** was found to be a *trans*-HCiAr-containing derivative of parishin A. In addition, two doublets of two sets of methylene groups at δ_H_ 2.78 (2H, d, *J* = 15.4 Hz) and 2.96 (2H, d, *J* = 15.4 Hz) in the CA unit were observed, which indicated symmetry, i.e., the *trans*-HCiAr was connected to GAr″. The HMBC correlation between H-3″ (δ_H_ 5.16) of GAr″ and C-9‴ of *trans*-HCiAr further suggested their connection (Figure 4). Consequently, **4** was determined to be 1,3-di-[4-*O*-(β-d-glucopyranosyl)benzyl]-2-{4-*O*-[3-*O*-(4-hydroxy-*trans*-cinnamoyl)-β-d-glucopyranosyl]benzyl} citrate.

Compound **5** had the same molecular formula (C_54_H_62_O_27_) as **4** and exhibited similar spectroscopic data (Figure S47). The significant difference in their NMR data lay in the HCiAr, which has a coupling constant of 12.8 Hz between H-7‴ (δ_H_ 6.90) and H-8‴ (δ_H_ 5.88), suggesting that it was a *cis*-HCiAr, whereas the signals of the parishin A unit were similar (Tables 2 and 3). The clear HMBC correlation between H-3″ (δ_H_ 5.14) of GAr″ and C-9‴ (δ 168.1) of *cis*-HCiAr was sufficient to help determine the linkage sequence (Figure S49). Thus, **5** was elucidated as 1,3-di-[4-*O*-(β-d-glucopyranosyl)benzyl]-2-{4-*O*-[3-*O*-(4-hydroxy-*cis*-cinnamoyl)-β-d-glucopyranosyl]benzyl}citrate.

Compound **6**, with a molecular formula of C_54_H_62_O_26_, has one oxygen less than **4** (Figure S55). A comparison of their NMR data suggested that they had similar parishin A unit signals, and two doublets were observed at δ_H_ 2.73 (2H, d, *J* = 15.4 Hz) and 2.88 (2H, d, *J* = 15.4 Hz), indicating that **6** had a symmetric structure (Tables 2 and 3). Differently, **6** had an additional *trans*-cinnamic acid residue (*trans*-CiAr) instead of *trans*-HCiAr according to the monosubstituted benzene ring signals at δ_H_ 7.59 (2H, d, *J* = 8.5 Hz, H-2‴, 6‴), 7.41 (2H, d, *J* = 8.5 Hz, H-3‴, 5‴), and 7.40 (1H, overlap, H-4‴), a pair of *trans* double bond signals at δ_H_ 7.68 (*J* = 16.2 Hz, H-7‴) and 6.53 (*J* = 16.2 Hz, H-8‴), and an ester carbonyl at δ_C_ 168.3 (C-9‴). In addition, a downfield shift at C-6″ (Δδ_C_ + 2.5) of GAr″ was also observed. These differences suggested that *trans*-CiAr was attached to the 6″-hydroxyl group of GAr″, which was further confirmed by the HMBC correlation between H-6″ (δ_H_ 4.35/4.55) of GAr″ and C-9‴ of *trans*-CiAr (Figure 4 and Figure S61). Accordingly, **6** was established as 1,3-di-[4-*O*-(β-d-glucopyranosyl)benzyl]-2-{4-*O*-[6-*O*-(*trans*-cinnamoyl)-β-d-glucopyranosyl]benzyl} citrate.

Compound **7** had the same molecular formula as **6** with similar spectral data (Figure S66). By comparison with the NMR data of **6** (Table 2 and Table 3), two doublets for two methylene groups in the CA unit showed that **7** was also a symmetrical molecule. The downfield shift of H-2″ (Δδ_H_ + 1.64) correlated with C-2″ (δ 75.3) suggested that *trans*-CiAr was linked to the 2″-hydroxyl group of GAr″, which was confirmed by the HMBC correlation of C-9‴ (δ 167.8) with H-2″ of GAr″ (Figure S72). Therefore, the structure of **7** was determined to be 1,3-di-[4-*O*-(β-d-glucopyranosyl)benzyl]-2-{4-*O*-[2-*O*-(*trans*-cinnamoyl)-β-d-glucopyranosyl]benzyl}citrate.

Additionally, eight known parishins **8**–**15**, parishin A (**8**),^19^ B (**9**),^21^ C (**10**),^21^ E (**11**),^22^ G (**12**),^23^ J (**13**),^24^ K (**14**),^24^ and Y (**15**),^25^ were assigned by comparison with NMR data from the literature. It should be noted that parishin K was initially reported and named in 2015,^24^ while other publications referred to a different structure without providing concrete evidence to support it.^26^

### 3.2 Neuroprotective Effects of Gastrodin Derivatives

**Figure 5:**
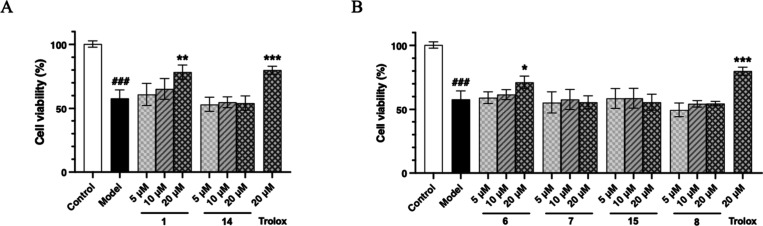
Effects of representative gastrodin derivatives **1** and **14** (A), and **6**, **7**, **8**, and **15** (B) on the H_2_O_2_-induced injury in PC12 cells. ^###^*P* < 0.001 vs control, ***P < 0.001 vs model, **P < 0.01 vs model, and *P < 0.05 vs model (*n* = 3; mean ± SD).

Next, a H_2_O_2_-induced neuronal injury model in PC12 cells was applied for neuroprotective activity evaluation *in vitro* (Table S12).^15^ Here, six representative gastrodin derivatives (**1**, **6**, **7**, **8**, **14**, and **15**) were chosen for evaluation and further structure–activity relationship analysis using Trolox as a positive control (Figure 5). The cell viability was significantly reduced to 57.93% within 24 h of exposure to 600 μM H_2_O_2_ compared to that of the control group. Remarkably, compound **1** demonstrated a promising neuroprotective effect by significantly reversing H_2_O_2_-induced PC12 cell injury in a concentration-dependent manner, increasing the cell viability to 78.44% at 20 μM (Figure 5A). However, compound **14** (parishin K), an isomer of **1** with a CA backbone, did not confer any protective effect when administered at the same treatment dose. Structural differences between compounds may have an effect on their biological activities. This outcome suggested that the ICA backbone was crucial in gastrodin derivatives, leading to a 20.51% increase in activity. The main difference between ICA and CA is the position of the hydroxyl group linkage on the backbone. In the ICA backbone, the hydroxyl group is located at C-1, while in the CA backbone, it is located at C-2. Thus, differences in the position of the hydroxyl group substituents on the backbone might influence the potential neuroprotective effects of these compounds. Additionally, it was important to mention that ICA, as an active unit, has evidenced its effectiveness in treating AD,^27^ countering oxidative stress,^28^ and mitigating heavy metal-induced neurotoxicity.^29^ Moreover, compound **6** also showed a concentration-dependent neuroprotective trend (Figure 5B). Given that the isomers (**7** and **15**) and the prototype parishin (**8**) of **6** had little neuroprotective activity, the activities of these compounds seemed to be affected by the esterification position (C-6″ of glucose in GAr).

### 3.3 Molecular Docking Simulation

**Figure 6:**
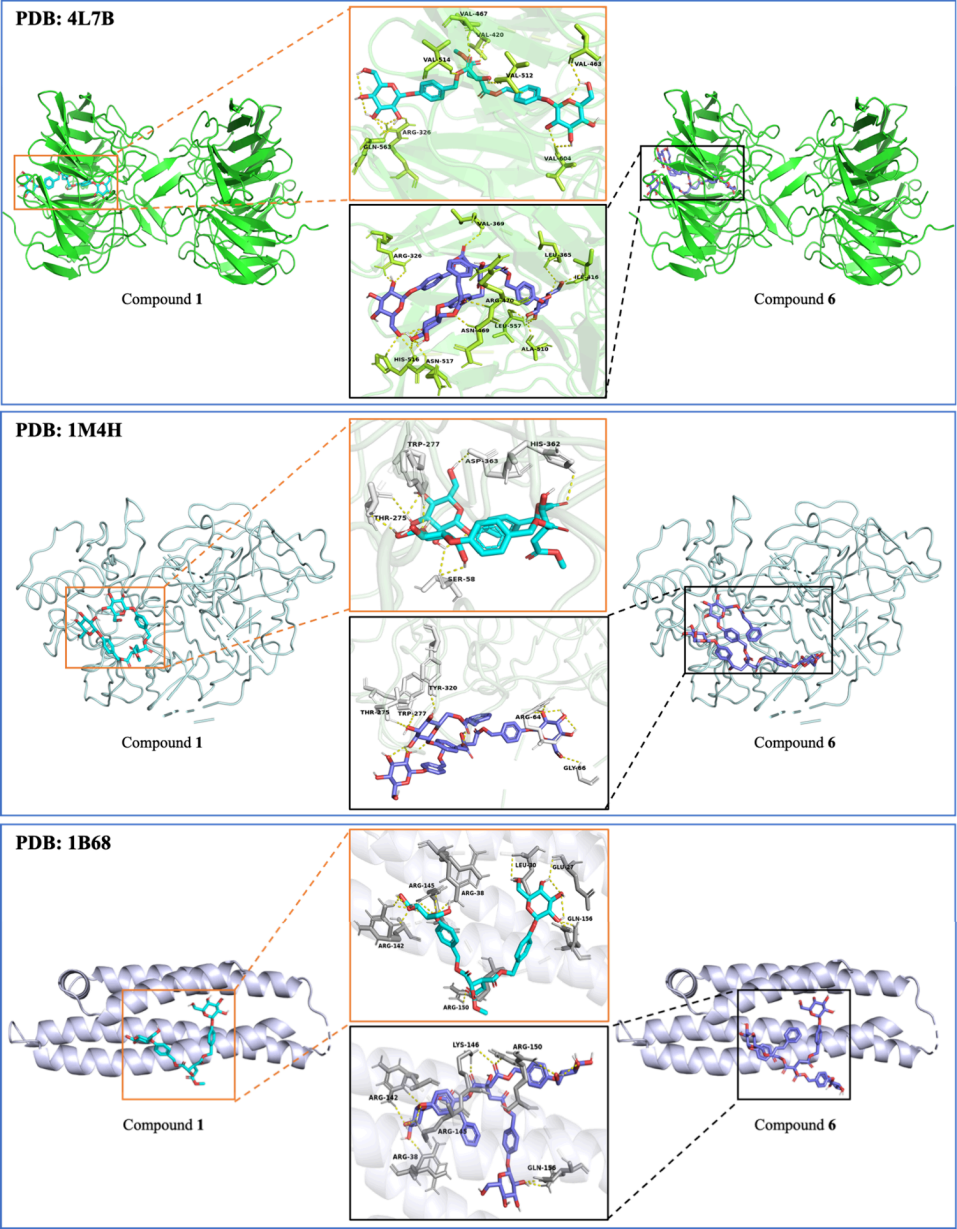
Molecular docking results of compounds **1** and **6** with Keap1-Nrf2 (PDB entry 4L7B), BACE1 (PDB entry 1M4H), and APOE4 (PDB entry 1B68).

AD is a multifactorial condition disease with an unclear etiology and complex pathogenesis.^30,31^ Molecular docking simulations will benefit the prediction of the action of drug molecules at the molecular level and provide a theoretical basis for subsequent target identification. Thus, three AD-related target proteins were selected for predicting the activities of compounds **1** and **6** by molecular docking based on the reference reviews.^31−36^ Keap1-Nrf2 is a crucial element in regulating oxidative stress and a promising therapeutic target for AD.^37^ Molecular docking studies provided detailed information on the interactions of **1** and **6** with the Keap1-Nrf2 protein (PDB entry 4L7B), which helped to predict their binding modes, recognize interacting residues, and calculate binding energies (Table S13). **1** and **6** exhibited potential inhibitory effects, with binding energies of −8.9 and −9.2 kcal/mol, respectively (Trolox: −7.5 kcal/mol). The 3D diagrams shown in Figure 6 displayed the interactions of two new active ingredients, **1** and **6**, with the Keap1-Nrf2 protein. Additionally, the complexity of AD has prompted a search for more potential targets. Currently, studies on AD are mainly targeting Aβ,^32^ and BACE1 has been associated with the production of Aβ.^38^ Additionally, APOE is a major genetic risk factor for AD, and its allele APOE4 further increases the risk of AD.^34^ Therefore, these two currently popular target proteins, BACE1 (PDB entry 1M4H) and APOE4 (PDB entry 1B68), were also selected for prediction (Figure 6). **1** and **6** exhibited better binding energies than did the positive control (Trolox), with binding energies of −10.0 and −10.3 kcal/mol for BACE1 (Trolox: −7.3 kcal/mol) and −7.7 and −8.5 kcal/mol for APOE4 (Trolox: −5.7 kcal/mol), respectively (Table S13).

## 4 Conclusions

In this study, we performed a systematic investigation of gastrodin derivatives in *G. elata*. A rare gastrodin isocitrate (**1**), six new parishin derivatives (**2**–**7**), and eight known parishins (**8**–**15**) were isolated from *G. elata* by various chromatographic separation techniques. Their structures were elucidated by a comprehensive analysis of HR-ESI-MS, NMR, and HPLC data, and the stereochemistry of compound **1** was further determined via ECD calculations. Remarkably, compound **1** was the first gastrodin isocitrate with a (1*R*,2*S*)-ICA backbone found in nature. The other six new derivatives were parishins with different substitutions, including two etherified parishins (**2**–**3**) and four esterified parishins (**4**–**7**). Subsequent bioactivity studies indicated that **1** and **6** could reverse H_2_O_2_-induced PC12 cell injury and had potential neuroprotective effects. Molecular docking further predicted their affinities with the target proteins. Our study uncovered potential novel bioactive ingredients in *G. elata* that deserve further exploration.

## Supporting Information Available

Spectroscopic data of compounds **1**–**7**; HPLC analysis after thiocarbamoyl-thiazolidine derivatization; ECD calculations of compound **1**; effects of gastrodin derivatives on the H_2_O_2_-induced PC12 cells injury model; and molecular docking results of representative compounds (PDF)
