# Supplementary material for: Isolation, Bioactivity, and Molecular Docking of a Rare Gastrodin Isocitrate and Diverse Parishin Derivatives from Gastrodia elata Blume
Source: ACS Omega. 2024 Mar 14;9(12):14520–9. doi: 10.1021/acsomega.4c00436 (PMC10976414; doi:10.1021/acsomega.4c00436)
Supplement: Supplementary file 1 — ao4c00436_si_001.pdf [file ao4c00436_si_001.pdf]

SUPPORTING INFORMATION

Isolation, Bioactivity, and Molecular Docking of a Rare Gastrodin Isocitrate and Diverse Parishin Derivatives from *Gastrodia elata* Blume

Jie Zhou[a], Jia-Qian Chen[a], Shilin Gong[a], Yu-Juan Ban[a], Li Zhang[a], Ying Liu[b], Jian-Lin Wu[a,*], Na Li[a,*]

[a] *State Key Laboratory of Quality Research in Chinese Medicine, Macau Institute for Applied Research in Medicine and Health, Macau University of Science and Technology, Taipa 999078 SAR, China*

[b] *School of Basic Medicinal Sciences and Nursing, Chengdu University, Chengdu, 610106, PR China*

Corresponding authors.

E-mail addresses: jlwu@must.edu.mo (J.L. Wu), nli@must.edu.mo (N. Li).

CONTENTS

Figure S

Figure S 1 HR-ESI-MS spectrum of compound **1**. 5
Figure S 2 ^{1}H NMR spectrum of compound **1** in MeOD. 5
Figure S 3 ^{13}C NMR spectrum of compound **1** in MeOD. 6
Figure S 4 DEPT135 NMR spectrum of compound **1** in MeOD. 6
Figure S 5 ^{1}H–^{1}H COSY NMR spectrum of compound **1** in MeOD. 7
Figure S 6 HSQC NMR spectrum of compound **1** in MeOD. 7
Figure S 7 HMBC NMR spectrum of compound **1** in MeOD. 8
Figure S 8 TOCSY NMR spectrum of compound **1** in MeOD. 8
Figure S 9 NOESY NMR spectrum of compound **1** in MeOD. 9
Figure S 10 IR spectrum of compound **1** (KBr). 9
Figure S 11 UV spectrum of compound **1** in MeOH. 10
Figure S 12 CD spectrum of compound **1** in MeOH. 10
Figure S 13 Derivatization-HPLC analysis for the determination of glucose configuration. 11
Figure S 14 HR-ESI-MS spectrum of compound **2**. 11
Figure S 15 ^{1}H NMR spectrum of compound **2** in MeOD. 12
Figure S 16 ^{13}C NMR spectrum of compound **2** in MeOD. 12
Figure S 17 DEPT135 NMR spectrum of compound **2** in MeOD. 13
Figure S 18 ^{1}H–^{1}H COSY NMR spectrum of compound **2** in MeOD. 13
Figure S 19 HSQC NMR spectrum of compound **2** in MeOD. 14
Figure S 20 HMBC NMR spectrum of compound **2** in MeOD. 14
Figure S 21 TOCSY NMR spectrum of compound **2** in MeOD. 15
Figure S 22 IR spectrum of compound **2** (KBr). 15
Figure S 23 UV spectrum of compound **2** in MeOH. 16
Figure S 24 CD spectrum of compound **2** in MeOH. 16
Figure S 25 HR-ESI-MS spectrum of compound **3**. 17
Figure S 26 ^{1}H NMR spectrum of compound **3** in MeOD. 17
Figure S 27 ^{13}C NMR spectrum of compound **3** in MeOD. 18
Figure S 28 DEPT135 NMR spectrum of compound **3** in MeOD. 18
Figure S 29 ^{1}H–^{1}H COSY NMR spectrum of compound **3** in MeOD. 19
Figure S 30 HSQC NMR spectrum of compound **3** in MeOD. 19
Figure S 31 HMBC NMR spectrum of compound **3** in MeOD. 20
Figure S 32 TOCSY NMR spectrum of compound **3** in MeOD. 20
Figure S 33 IR spectrum of compound **3** (KBr). 21
Figure S 34 UV spectrum of compound **3** in MeOH. 21
Figure S 35 CD spectrum of compound **3** in MeOH. 22
Figure S 36 HR-ESI-MS spectrum of compound **4**. 22
Figure S 37 ^{1}H NMR spectrum of compound **4** in MeOD. 23
Figure S 38 ^{13}C NMR spectrum of compound **4** in MeOD. 23
Figure S 39 DEPT135 NMR spectrum of compound **4** in MeOD. 24
Figure S 40 ^{1}H–^{1}H COSY NMR spectrum of compound **4** in MeOD. 24
Figure S 41 HSQC NMR spectrum of compound **4** in MeOD. 25
Figure S 42 HMBC NMR spectrum of compound **4** in MeOD. 25

Figure S 43 TOCSY NMR spectrum of compound **4** in MeOD....................................26
Figure S 44 IR spectrum of compound **4** (KBr)...26
Figure S 45 UV spectrum of compound **4** in MeOH. 27
Figure S 46 CD spectrum of compound **4** in MeOH. 27
Figure S 47 HR-ESI-MS spectrum of compound **5**. 28
Figure S 48 ^{1}H NMR spectrum of compound **5** in MeOD...28
Figure S 49 Comparison of ^{1}H NMR spectra between compound **4** and compound **5**...29
Figure S 50 ^{13}C NMR spectrum of compound **5** in MeOD..29
Figure S 51 DEPT135 NMR spectrum of compound **5** in MeOD.................................30
Figure S 52 ^{1}H–^{1}H COSY NMR spectrum of compound **5** in MeOD...........................30
Figure S 53 HSQC NMR spectrum of compound **5** in MeOD. 31
Figure S 54 HMBC NMR spectrum of compound **5** in MeOD. 31
Figure S 55 HR-ESI-MS spectrum of compound **6**. 32
Figure S 56 ^{1}H NMR spectrum of compound **6** in MeOD...32
Figure S 57 ^{13}C NMR spectrum of compound **6** in MeOD..33
Figure S 58 DEPT135 NMR spectrum of compound **6** in MeOD.................................33
Figure S 59 ^{1}H–^{1}H COSY NMR spectrum of compound **6** in MeOD...........................34
Figure S 60 HSQC NMR spectrum of compound **6** in MeOD. 34
Figure S 61 HMBC NMR spectrum of compound **6** in MeOD. 35
Figure S 62 TOCSY NMR spectrum of compound **6** in MeOD....................................35
Figure S 63 IR spectrum of compound **6** (KBr)...36
Figure S 64 UV spectrum of compound **6** in MeOH. 36
Figure S 65 CD spectrum of compound **6** in MeOH. 37
Figure S 66 HR-ESI-MS spectrum of compound **7**. 37
Figure S 67 ^{1}H NMR spectrum of compound **7** in MeOD...38
Figure S 68 ^{13}C NMR spectrum of compound **7** in MeOD..38
Figure S 69 DEPT135 NMR spectrum of compound **7** in MeOD.................................39
Figure S 70 ^{1}H–^{1}H COSY NMR spectrum of compound **7** in MeOD...........................39
Figure S 71 HSQC NMR spectrum of compound **7** in MeOD. 40
Figure S 72 HMBC NMR spectrum of compound **7** in MeOD. 40
Figure S 73 TOCSY NMR spectrum of compound **7** in MeOD....................................41
Figure S 74 IR spectrum of compound **7** (KBr)...41
Figure S 75 UV spectrum of compound **7** in MeOH. 42
Figure S 76 CD spectrum of compound **7** in MeOH. 42

Table S

Table S 1 Gibbs free energies[a] and equilibrium populations[b] of low-energy conformers of compound **1**. 43
Table S 2 Cartesian coordinates for the low-energy reoptimized random search conformers of **1**-1 at B3LYP-D3(BJ)/6-31G* level of theory in methanol. 44
Table S 3 Cartesian coordinates for the low-energy reoptimized random search conformers of **1**-2 at B3LYP-D3(BJ)/6-31G* level of theory in methanol. 47
Table S 4 Cartesian coordinates for the low-energy reoptimized random search conformers of **1**-3 at B3LYP-D3(BJ)/6-31G* level of theory in methanol. 50
Table S 5 Cartesian coordinates for the low-energy reoptimized random search conformers of **1**-4 at B3LYP-D3(BJ)/6-31G* level of theory in methanol. 53
Table S 6 Cartesian coordinates for the low-energy reoptimized random search conformers of **1**-5 at B3LYP-D3(BJ)/6-31G* level of theory in methanol. 56
Table S 7 Cartesian coordinates for the low-energy reoptimized random search conformers of **1**-6 at B3LYP-D3(BJ)/6-31G* level of theory in methanol. 59
Table S 8 Cartesian coordinates for the low-energy reoptimized random search conformers of **1**-7 at B3LYP-D3(BJ)/6-31G* level of theory in methanol. 62
Table S 9 Cartesian coordinates for the low-energy reoptimized random search conformers of **1**-8 at B3LYP-D3(BJ)/6-31G* level of theory in methanol. 65
Table S 10 Cartesian coordinates for the low-energy reoptimized random search conformers of **1**-9 at B3LYP-D3(BJ)/6-31G* level of theory in methanol. 68
Table S 11 Cartesian coordinates for the low-energy reoptimized random search conformers of **1**-10 at B3LYP-D3(BJ)/6-31G* level of theory in methanol. 71
Table S 12 Effects of gastrodin derivatives in the H_2O_2-induced PC12 cells injury model 74
Table S 13 Molecular docking results of representative compounds with Keap1-Nrf2 (PDB: 4L7B), BACE1 (PDB: 1M4H), and APOE4 (PDB: 1B68). 75

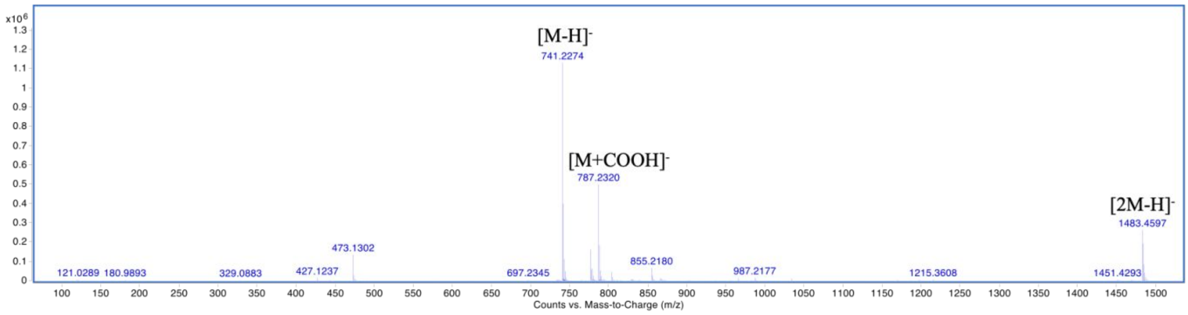

Formula (M)	Score (MFG)	*m/z*	*m/z* (calcd.)	Error (ppm)	DBE
$C_{33}H_{42}O_{19}$	91.37	741.2274	741.2248	-3.17	13

Figure S 1 HR-ESI-MS spectrum of compound **1**.

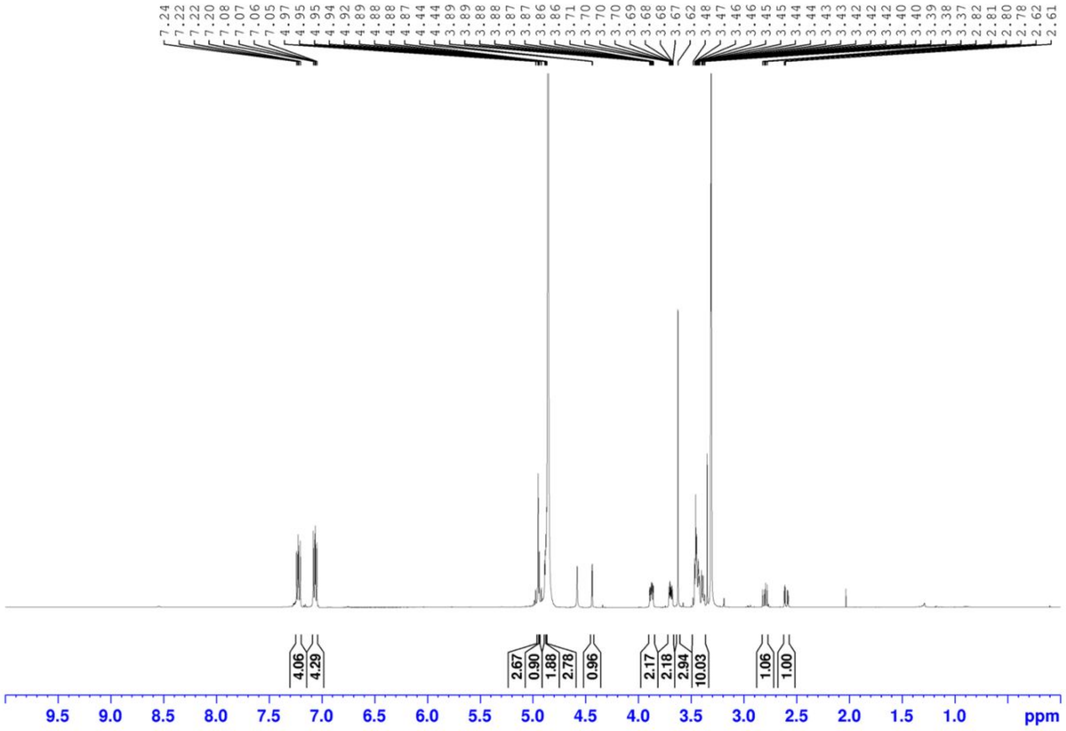

Figure S 2 ^{1}H NMR spectrum of compound **1** in MeOD.

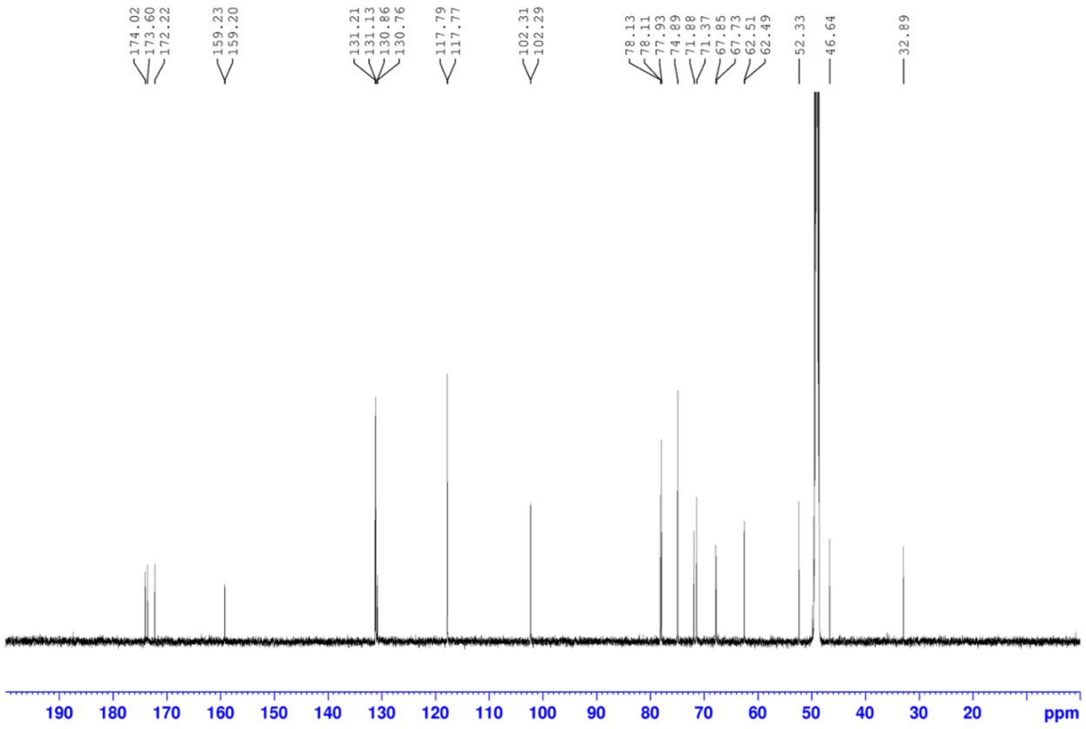

Figure S 3 ^{13}C NMR spectrum of compound **1** in MeOD.

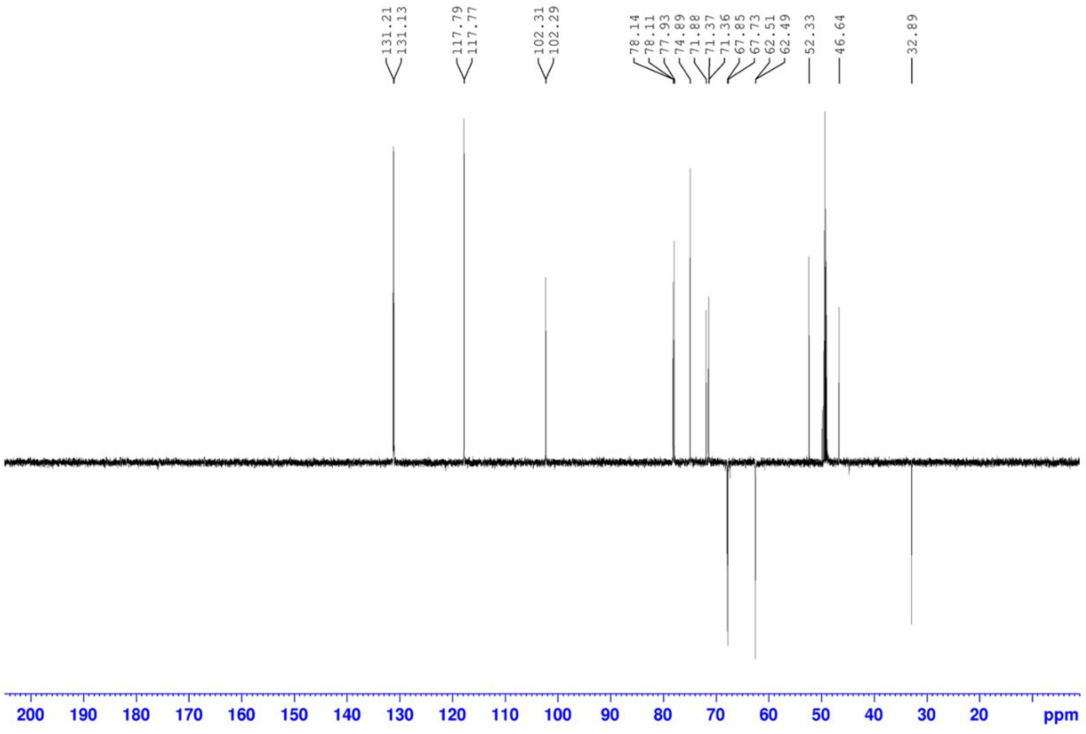

Figure S 4 DEPT135 NMR spectrum of compound **1** in MeOD.

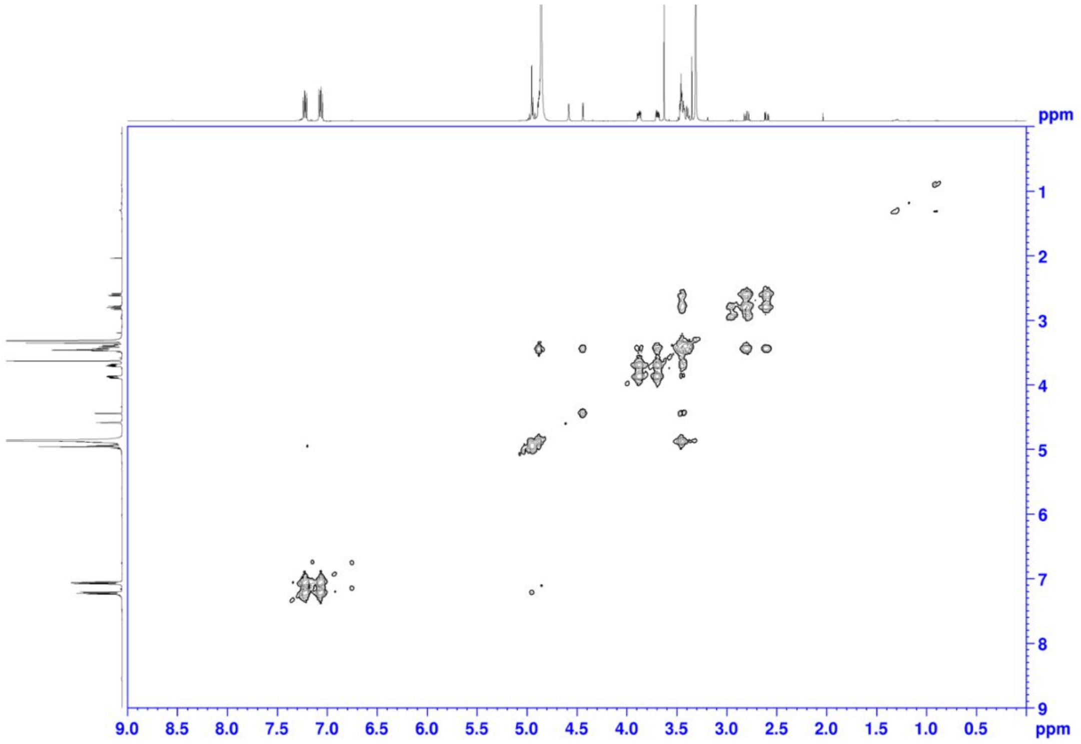

Figure S 5 ^{1}H–^{1}H COSY NMR spectrum of compound **1** in MeOD.

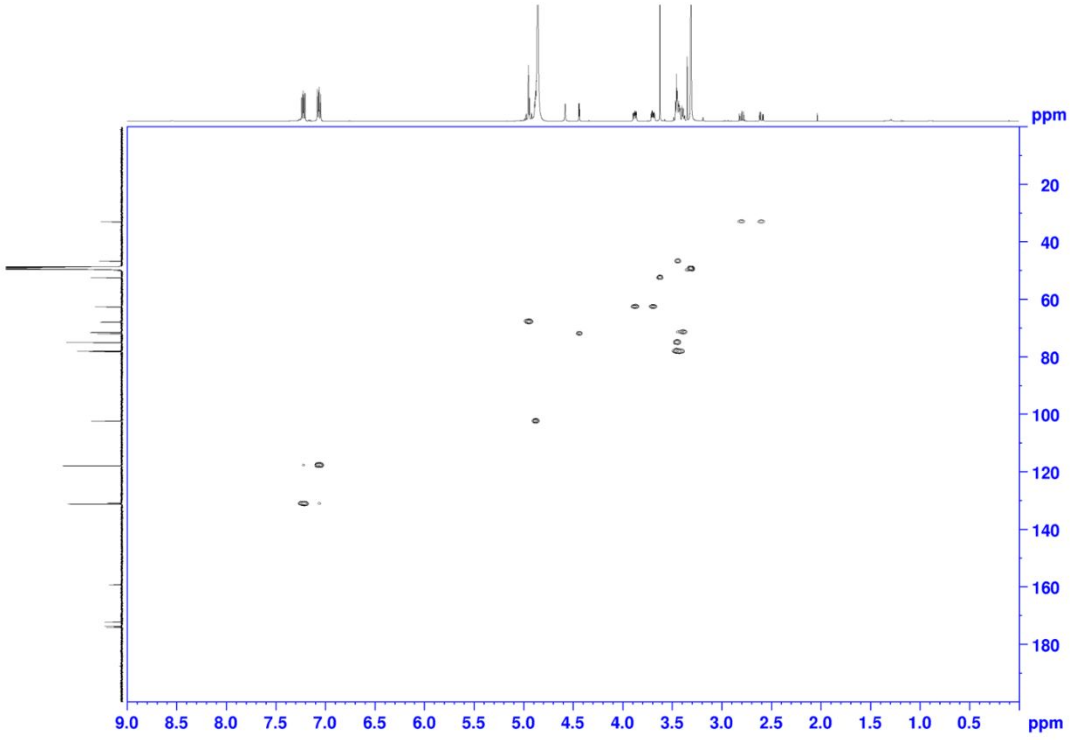

Figure S 6 HSQC NMR spectrum of compound **1** in MeOD.

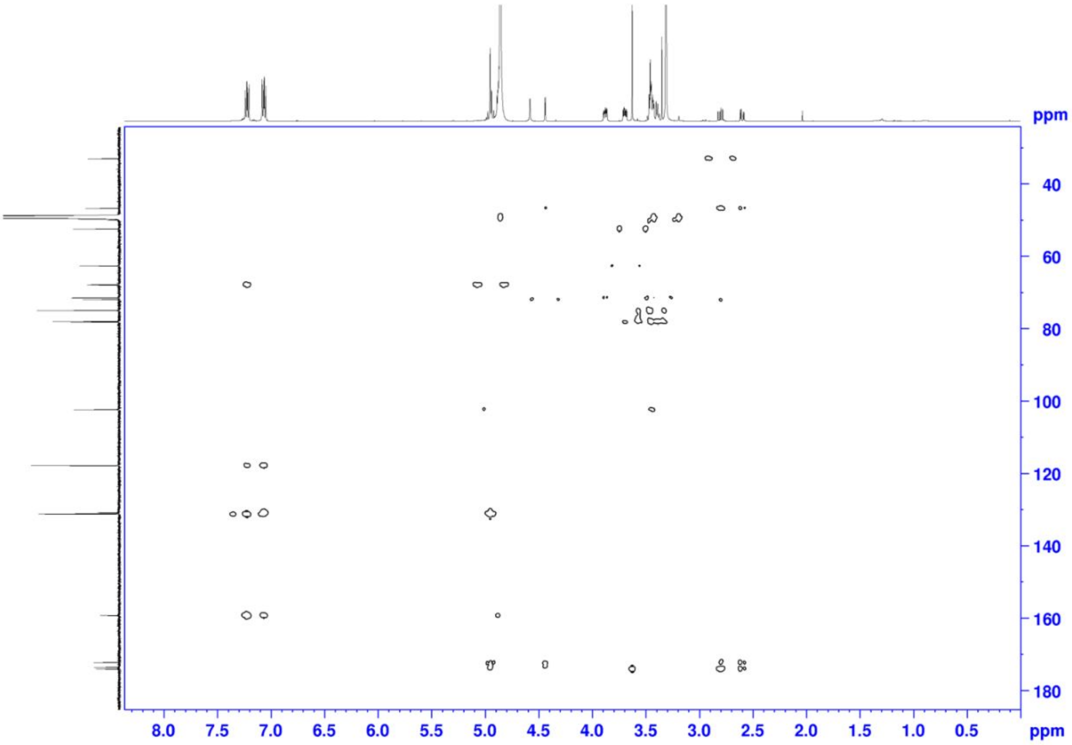

Figure S 7 HMBC NMR spectrum of compound **1** in MeOD.

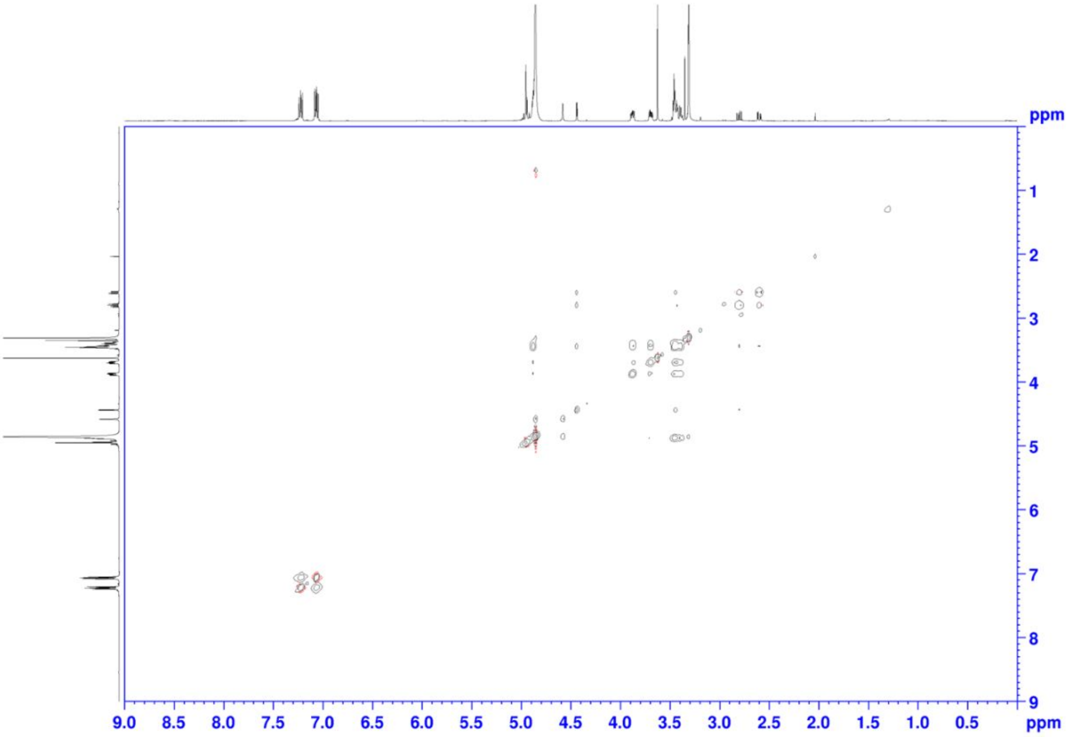

Figure S 8 TOCSY NMR spectrum of compound **1** in MeOD.

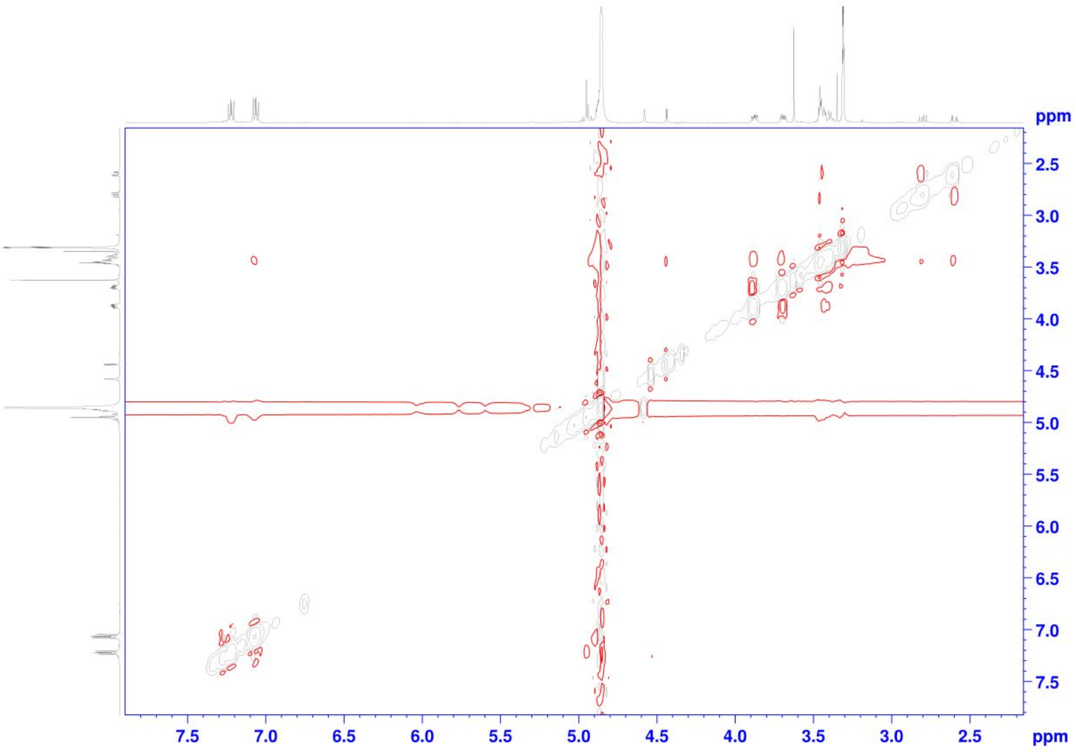

Figure S 9 NOESY NMR spectrum of compound **1** in MeOD.

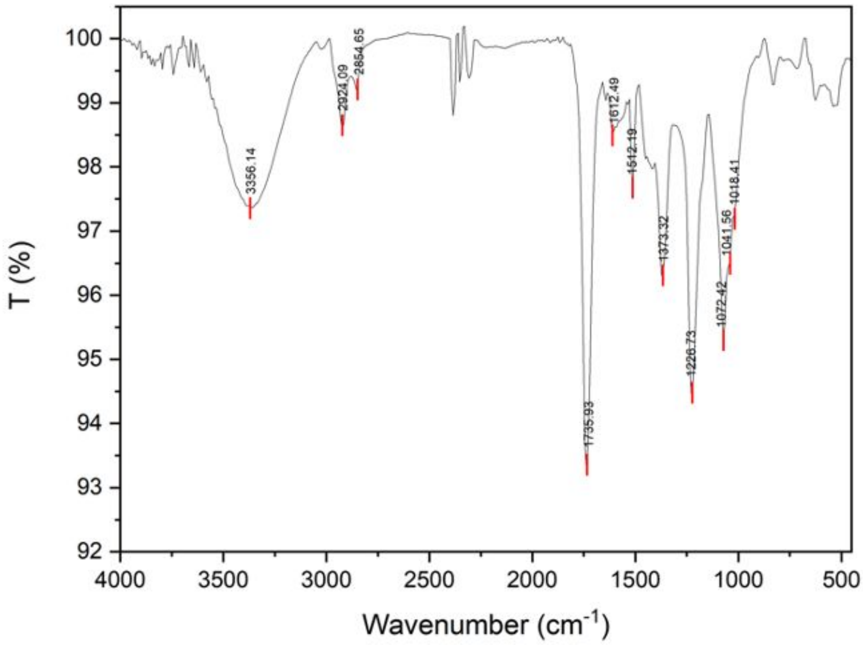

Figure S 10 IR spectrum of compound **1** (KBr).

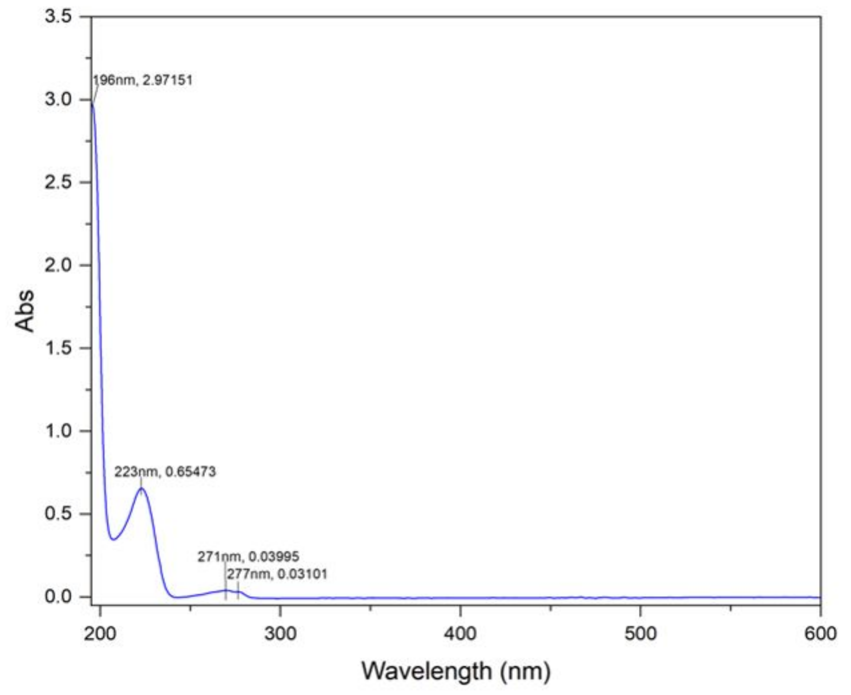

Figure S 11 UV spectrum of compound **1** in MeOH.

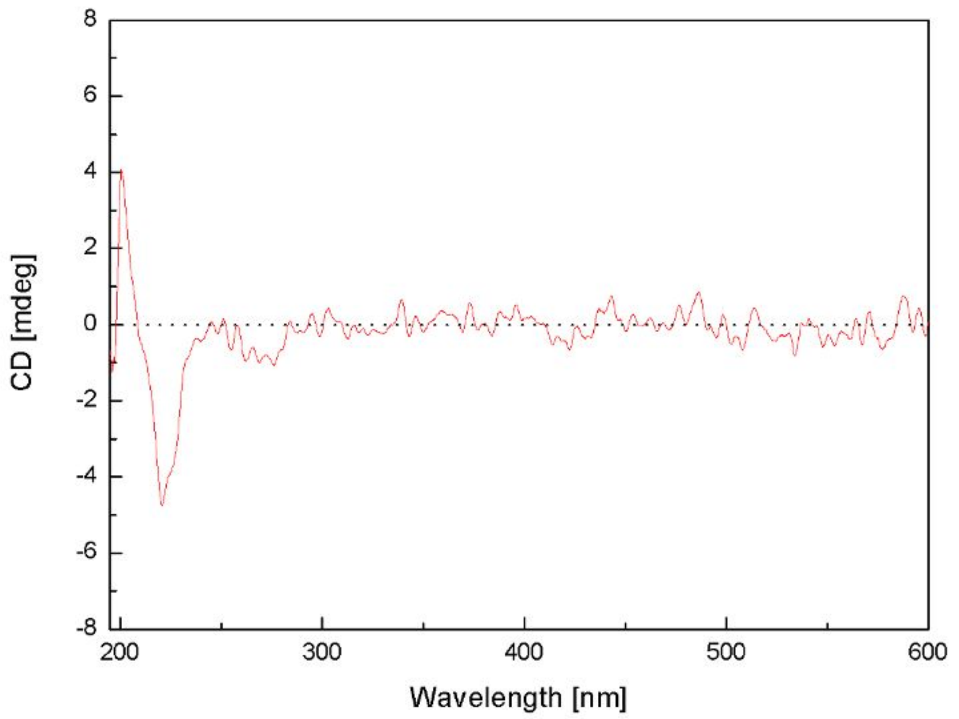

Figure S 12 CD spectrum of compound **1** in MeOH.

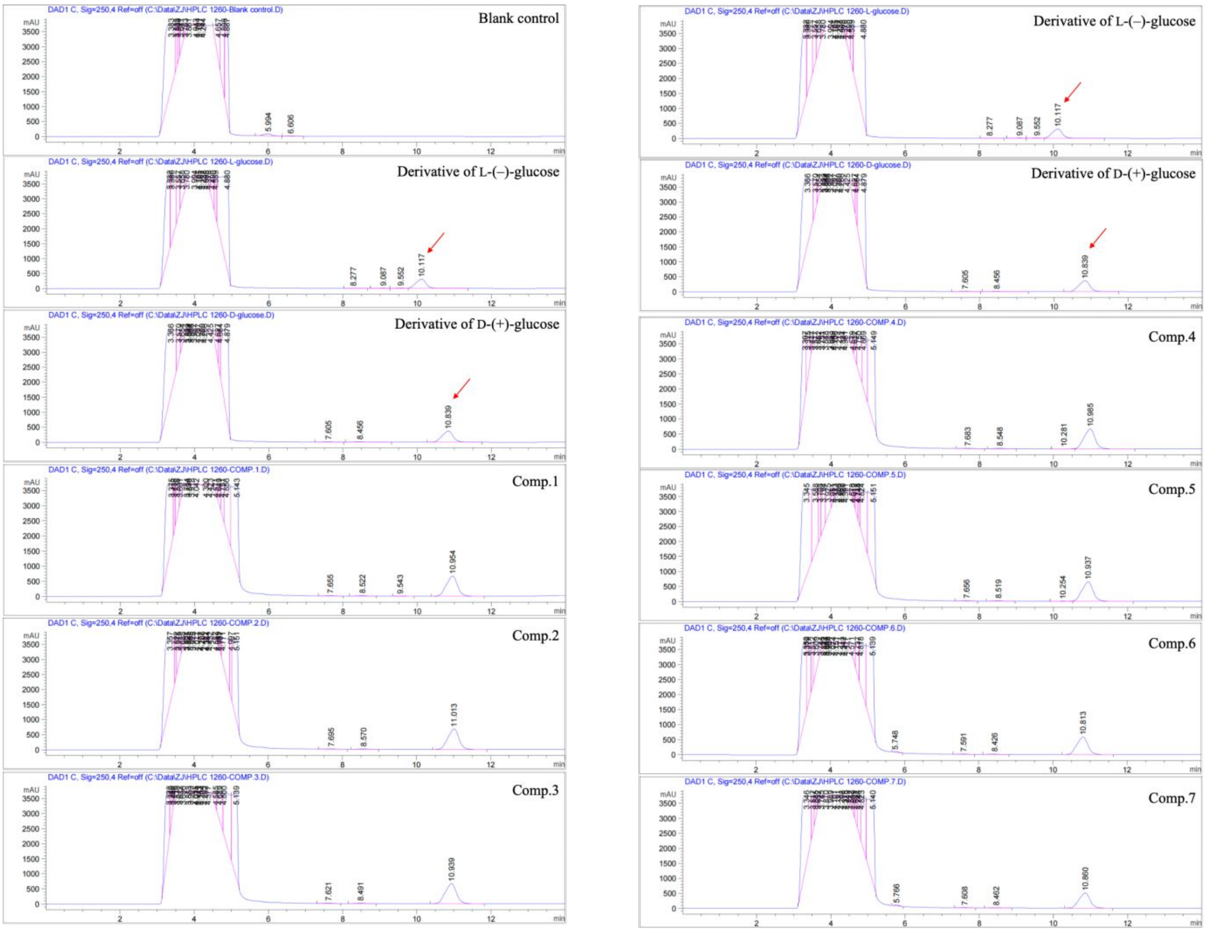

Figure S 13 Derivatization-HPLC analysis for the determination of glucose configuration.

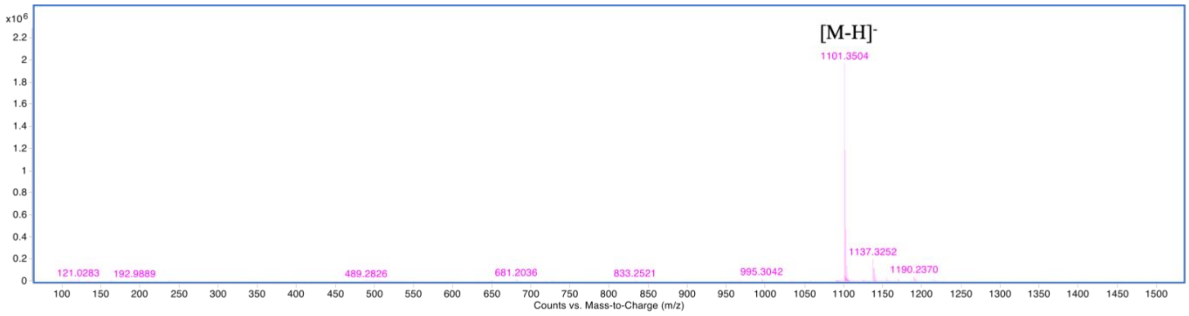

Formula (M)	Score (MFG)	*m/z*	*m/z* (calcd.)	Error (ppm)	DBE
$C_{52}H_{62}O_{26}$	94.81	1101.3504	1101.3457	-4.25	22

Figure S 14 HR-ESI-MS spectrum of compound **2**.

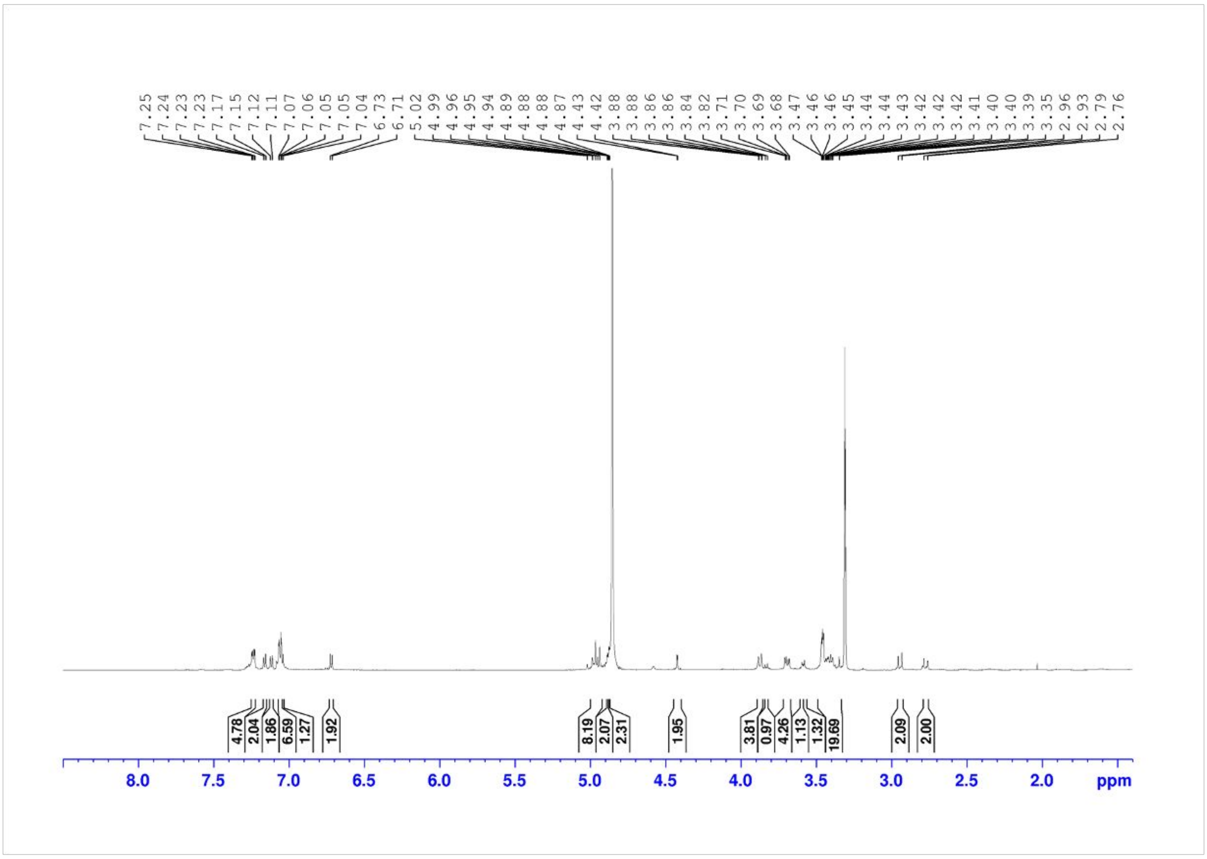

Figure S 15 ^{1}H NMR spectrum of compound **2** in MeOD.

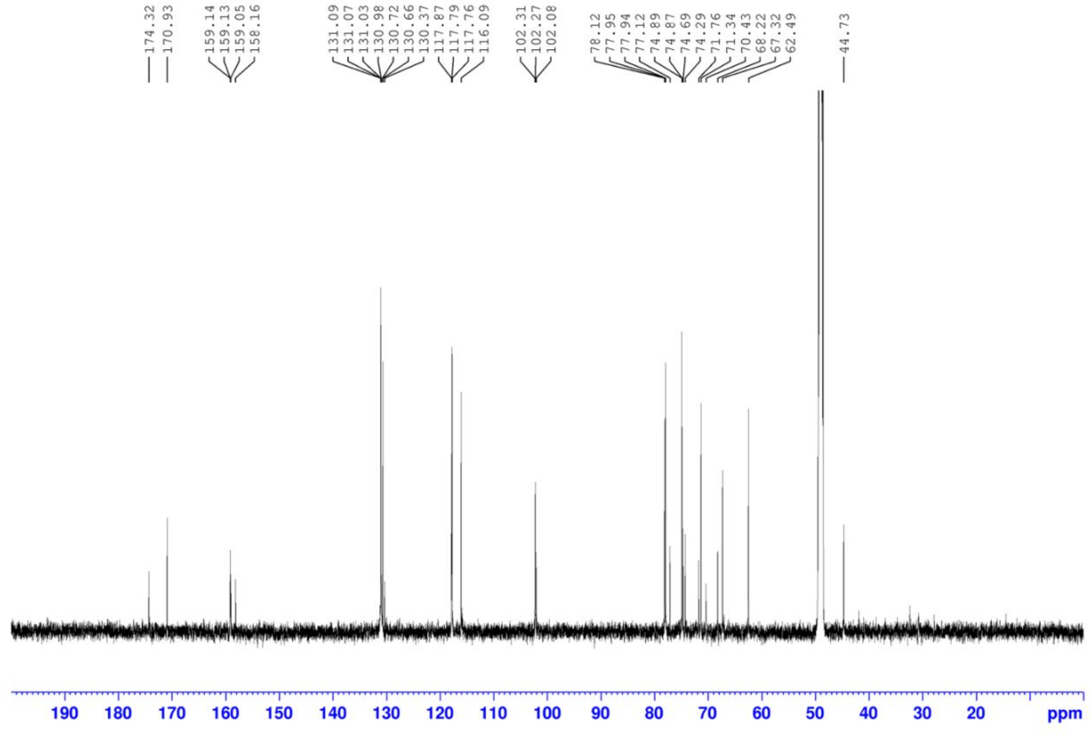

Figure S 16 ^{13}C NMR spectrum of compound **2** in MeOD.

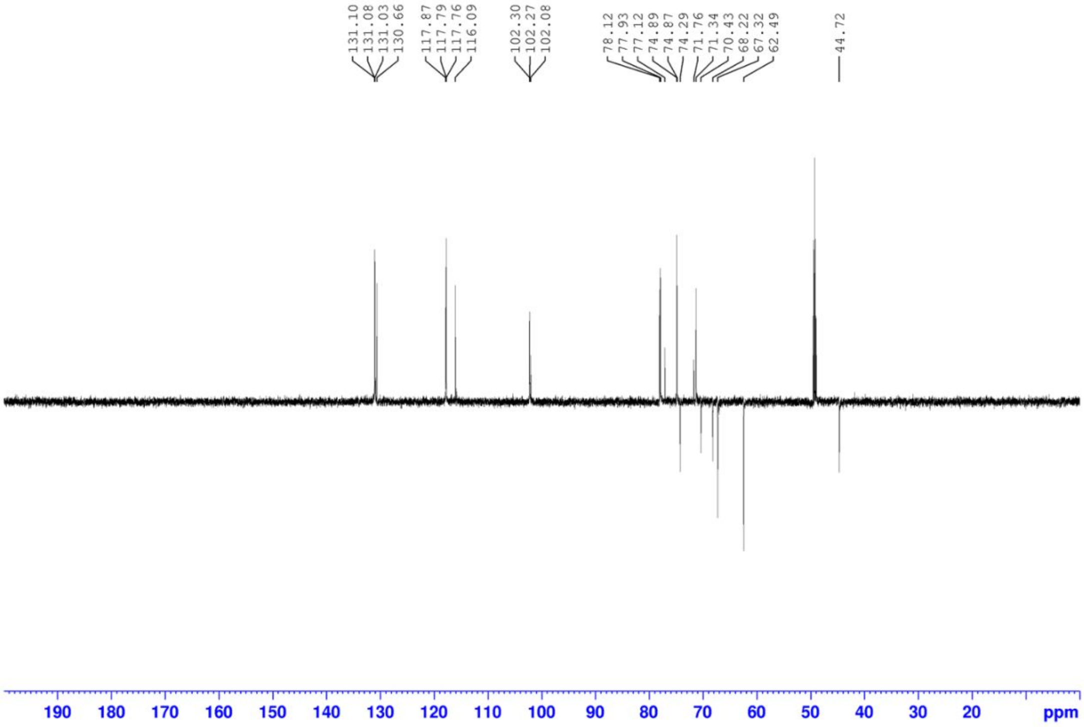

Figure S 17 DEPT135 NMR spectrum of compound **2** in MeOD.

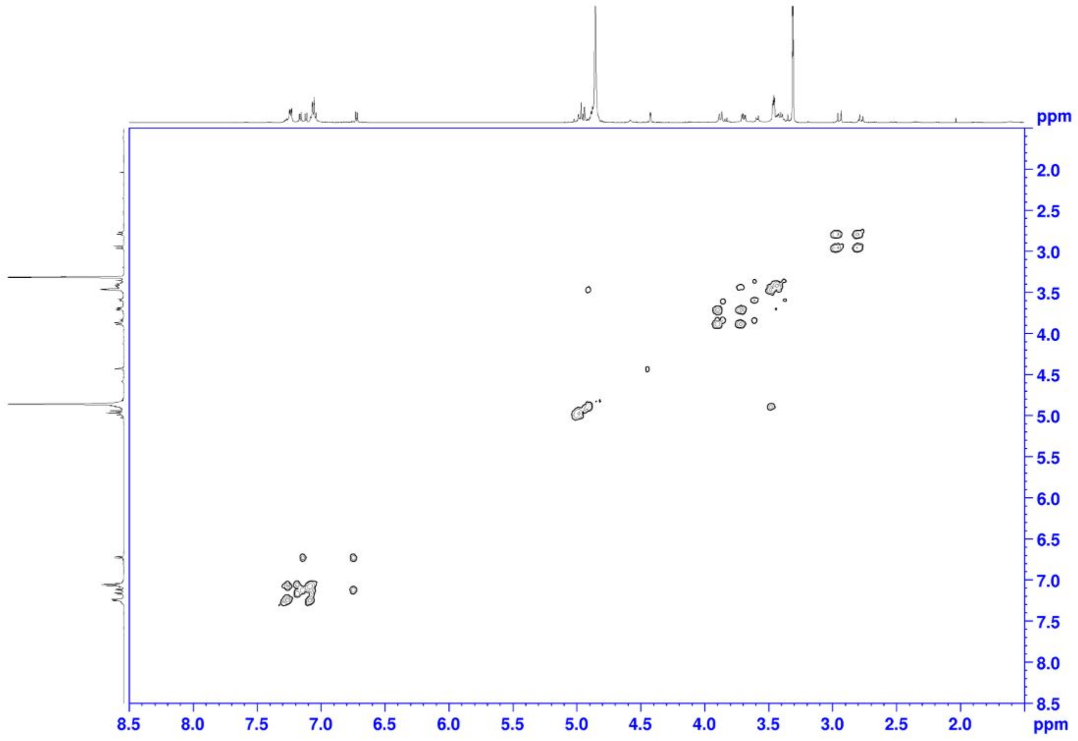

Figure S 18 ^{1}H–^{1}H COSY NMR spectrum of compound **2** in MeOD.

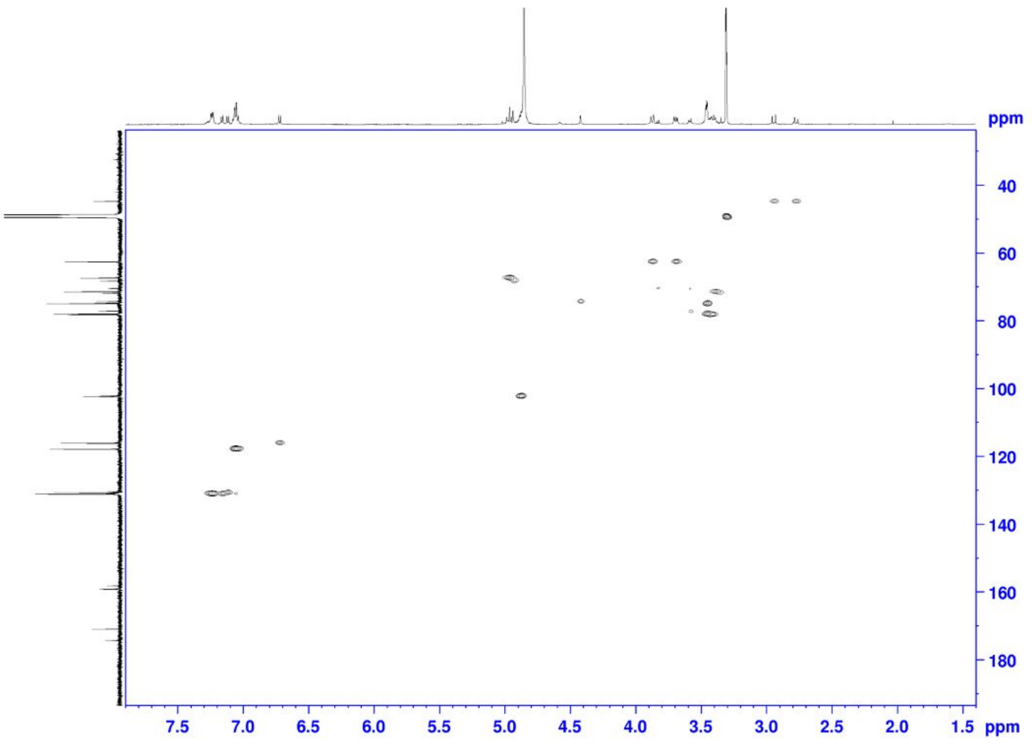

Figure S 19 HSQC NMR spectrum of compound **2** in MeOD.

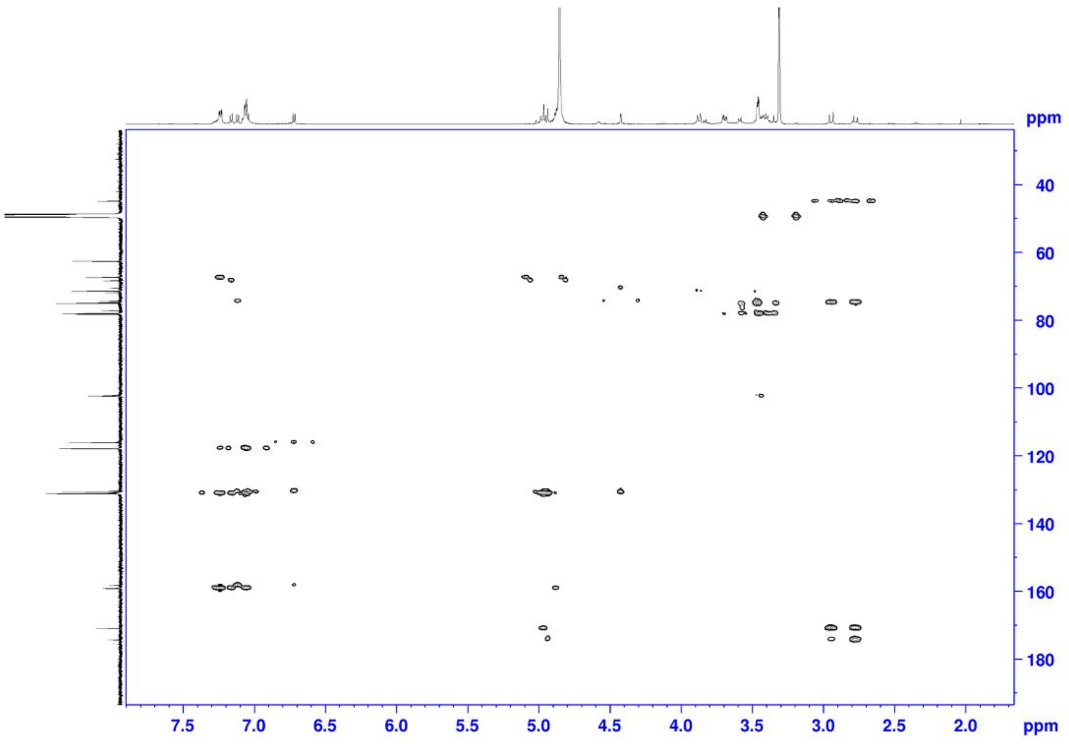

Figure S 20 HMBC NMR spectrum of compound **2** in MeOD.

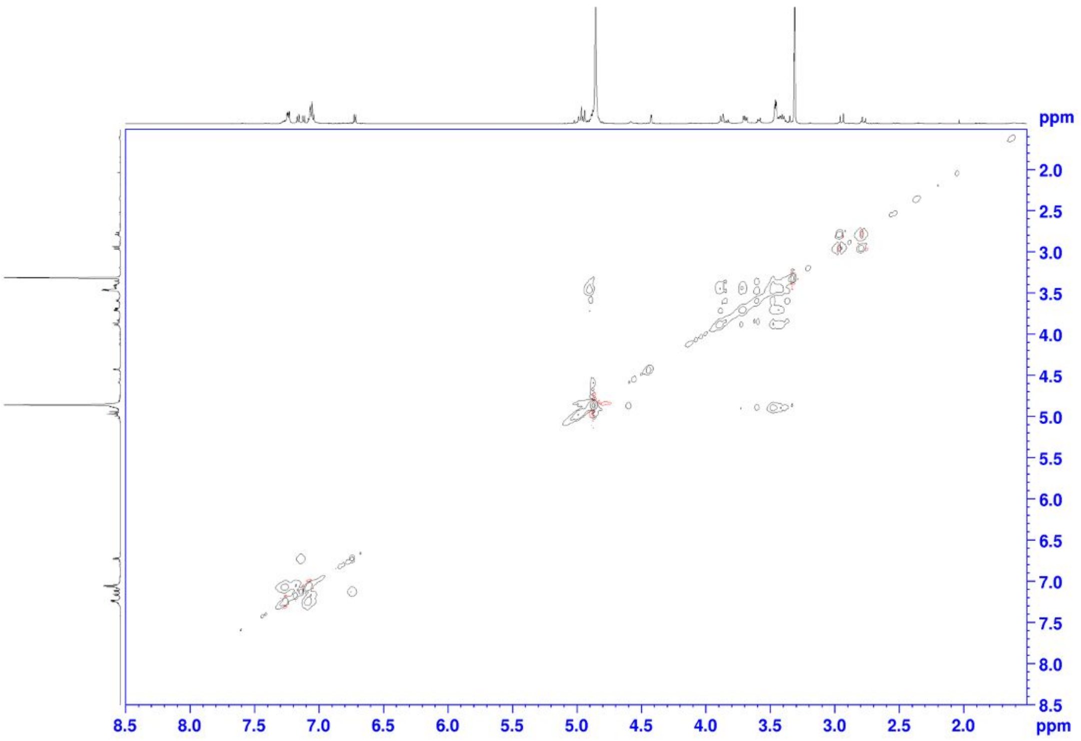

Figure S 21 TOCSY NMR spectrum of compound **2** in MeOD.

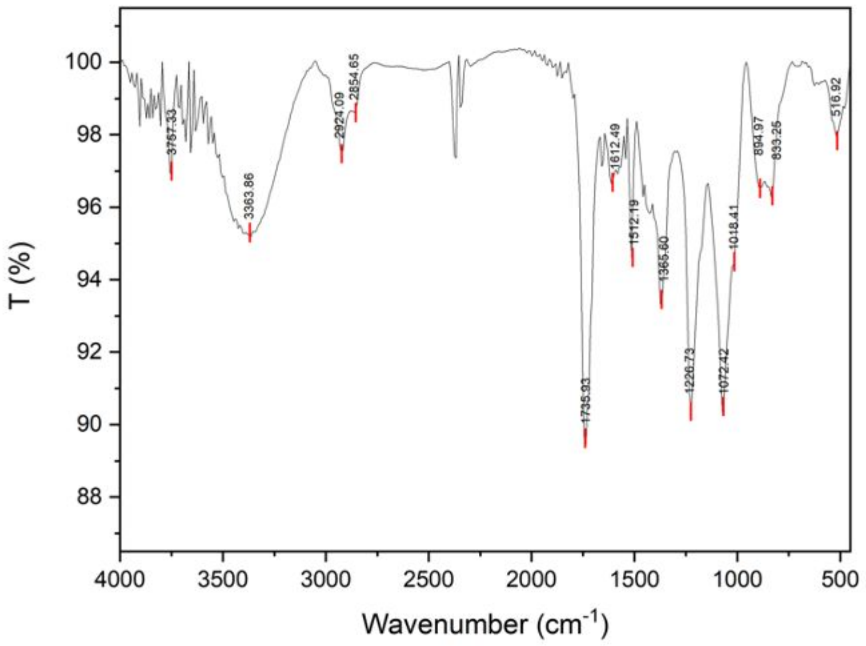

Figure S 22 IR spectrum of compound **2** (KBr).

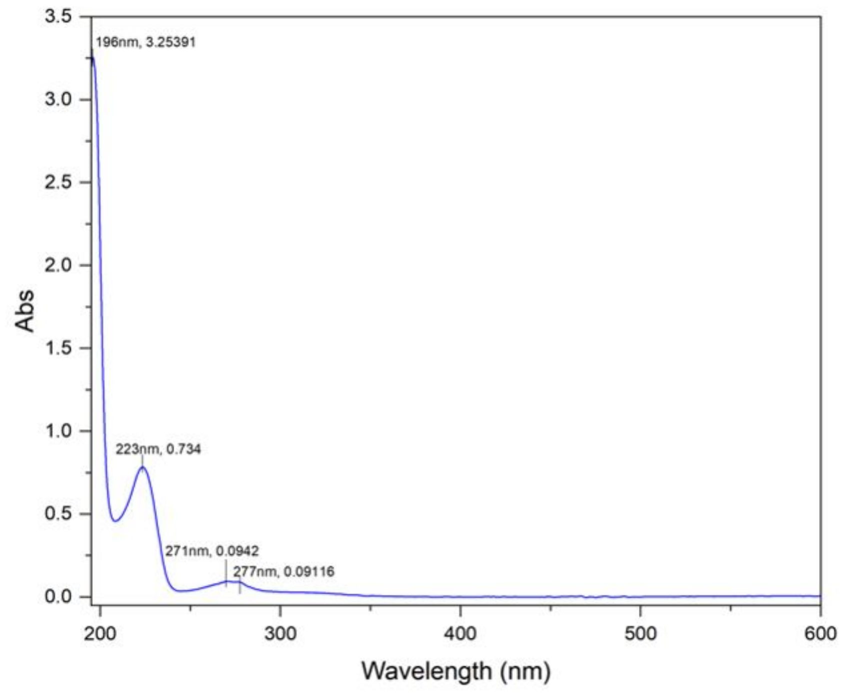

Figure S 23 UV spectrum of compound **2** in MeOH.

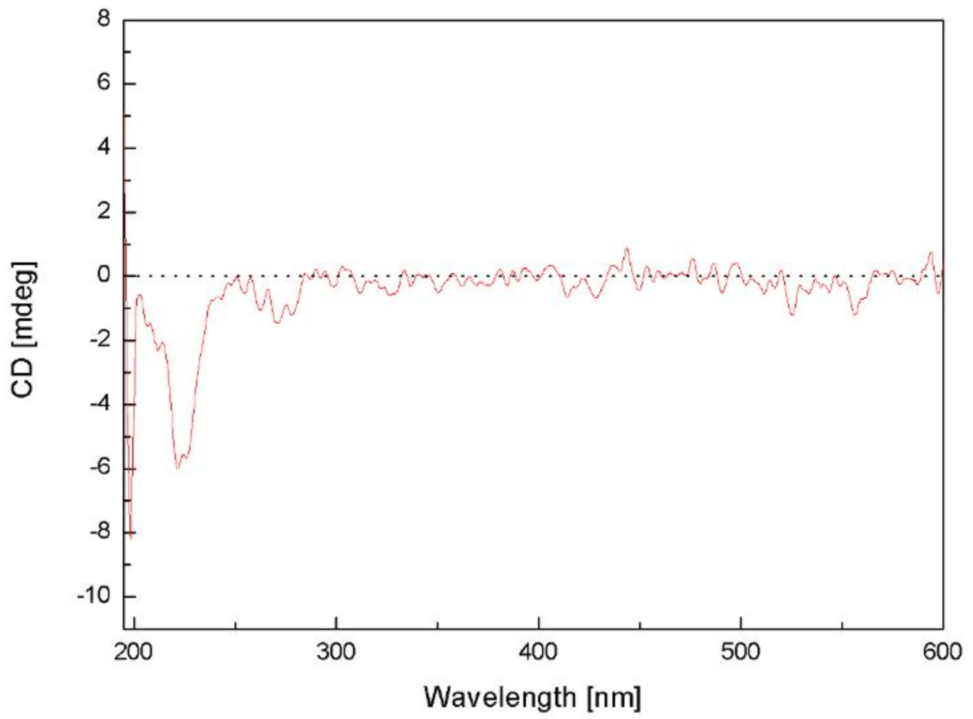

Figure S 24 CD spectrum of compound **2** in MeOH.

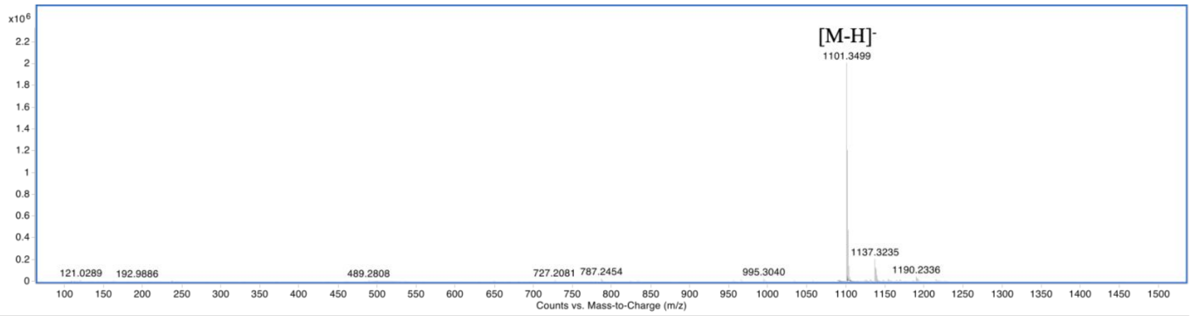

Formula (M)	Score (MFG)	*m/z*	*m/z* (calcd.)	Error (ppm)	DBE
$C_{52}H_{62}O_{26}$	93.67	1101.3499	1101.3457	-3.75	22

Figure S 25 HR-ESI-MS spectrum of compound **3**.

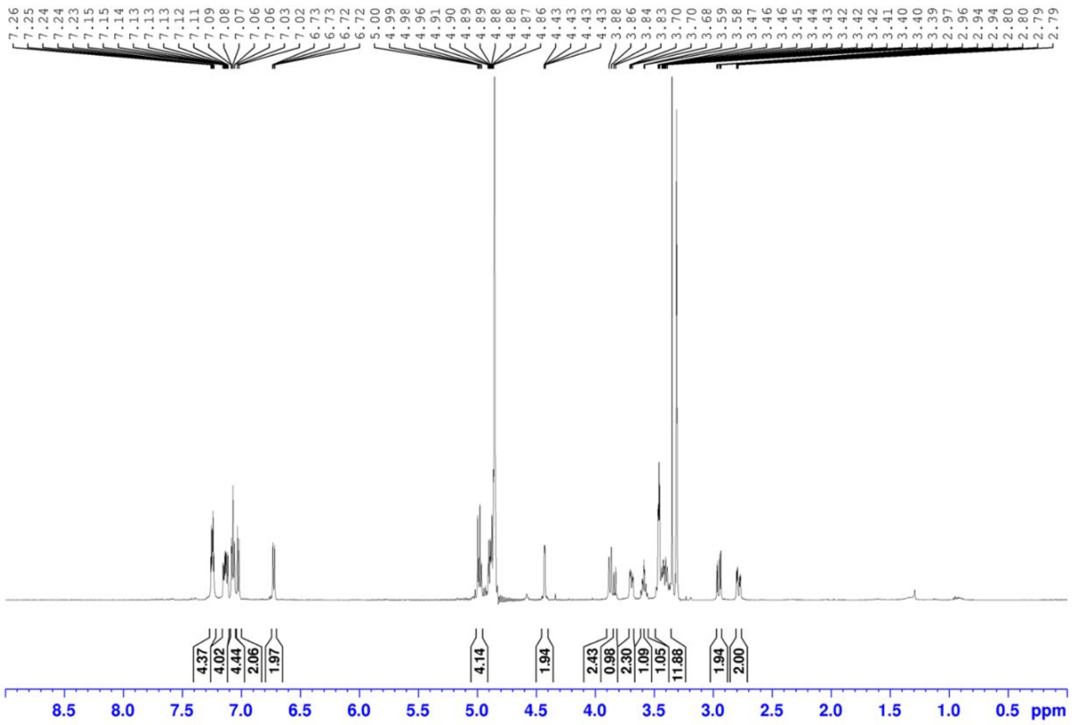

Figure S 26 ^{1}H NMR spectrum of compound **3** in MeOD.

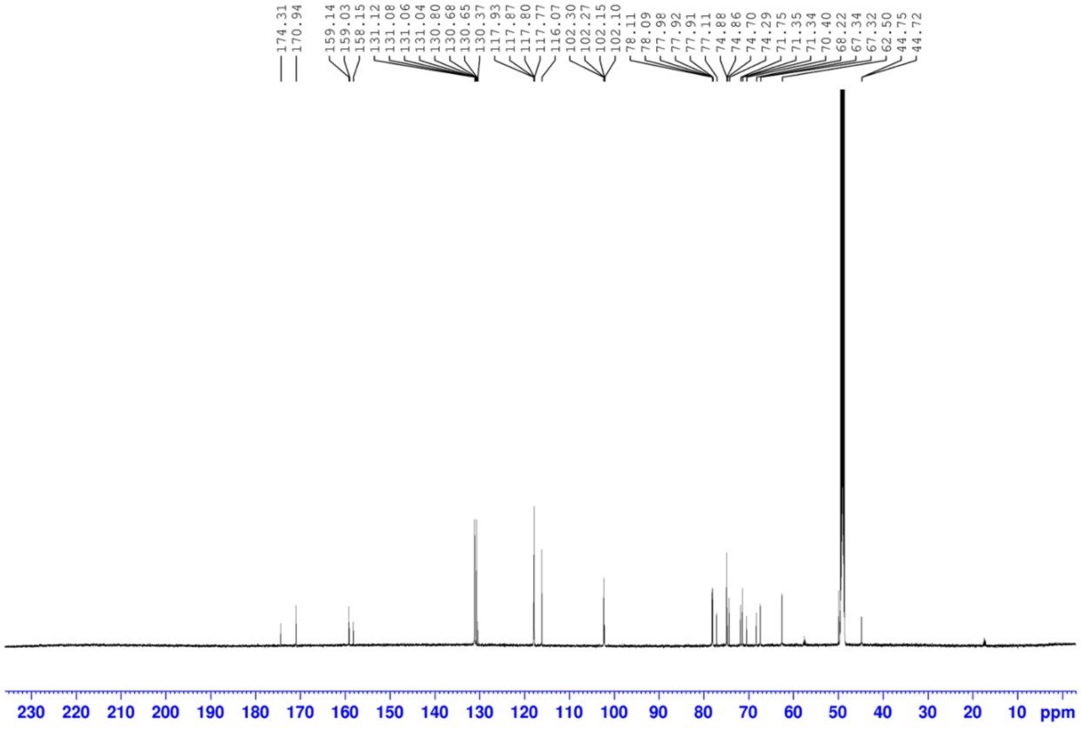

Figure S 27 ^{13}C NMR spectrum of compound **3** in MeOD.

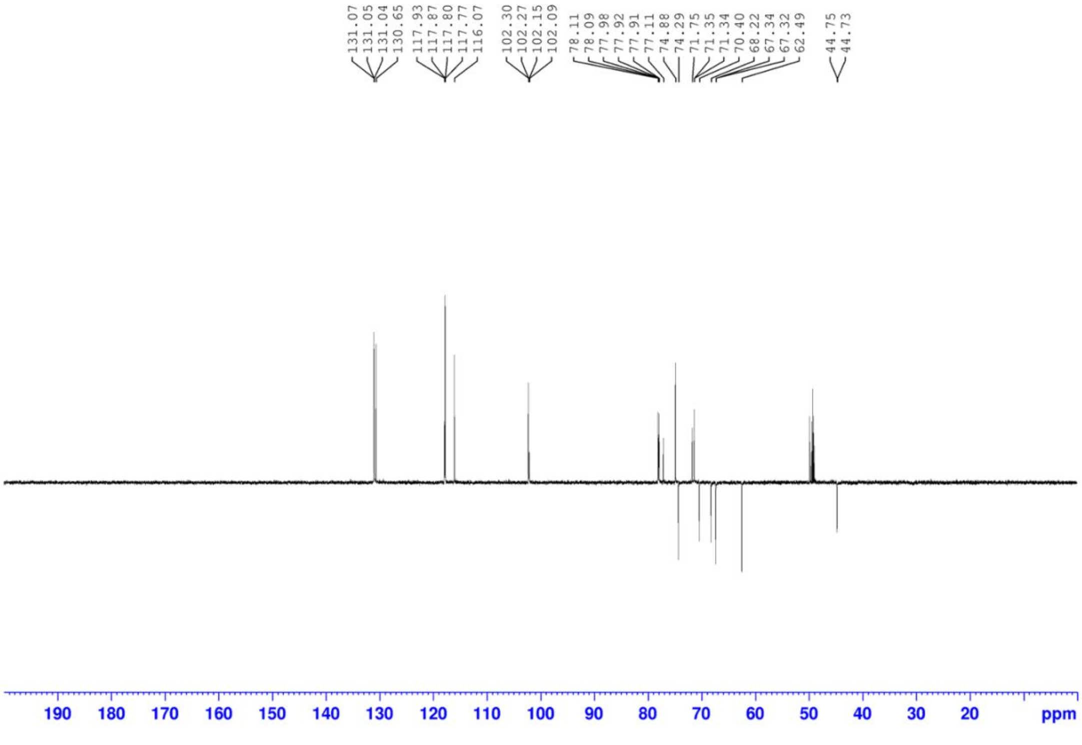

Figure S 28 DEPT135 NMR spectrum of compound **3** in MeOD.

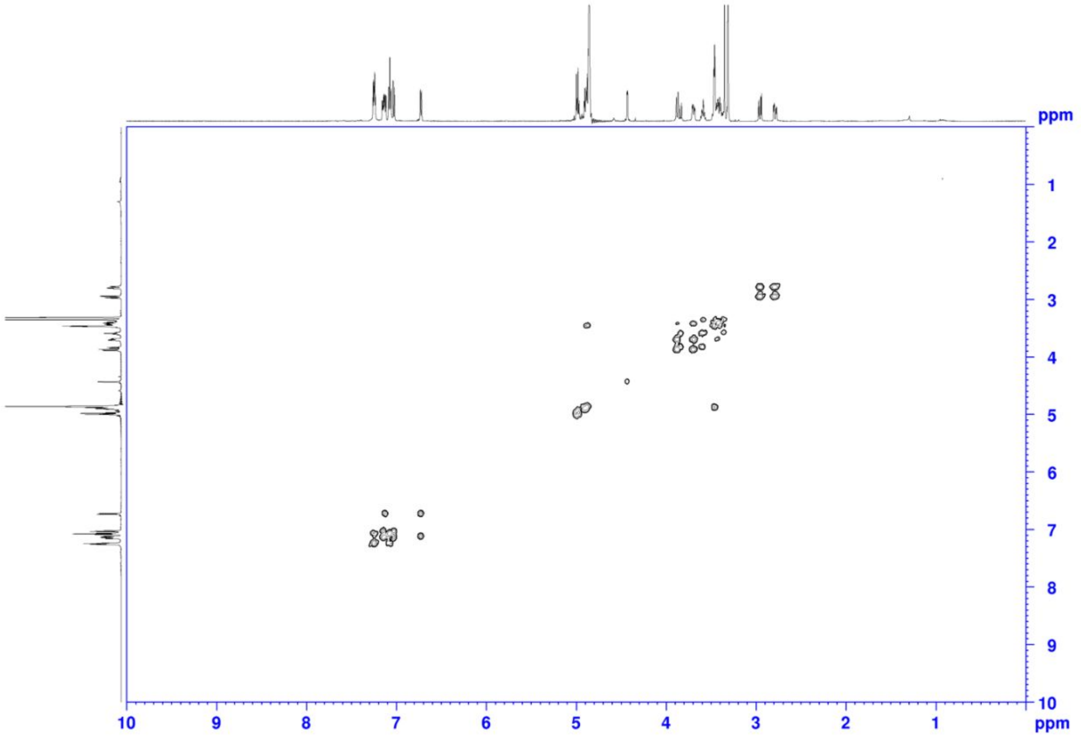

Figure S 29 ^{1}H–^{1}H COSY NMR spectrum of compound **3** in MeOD.

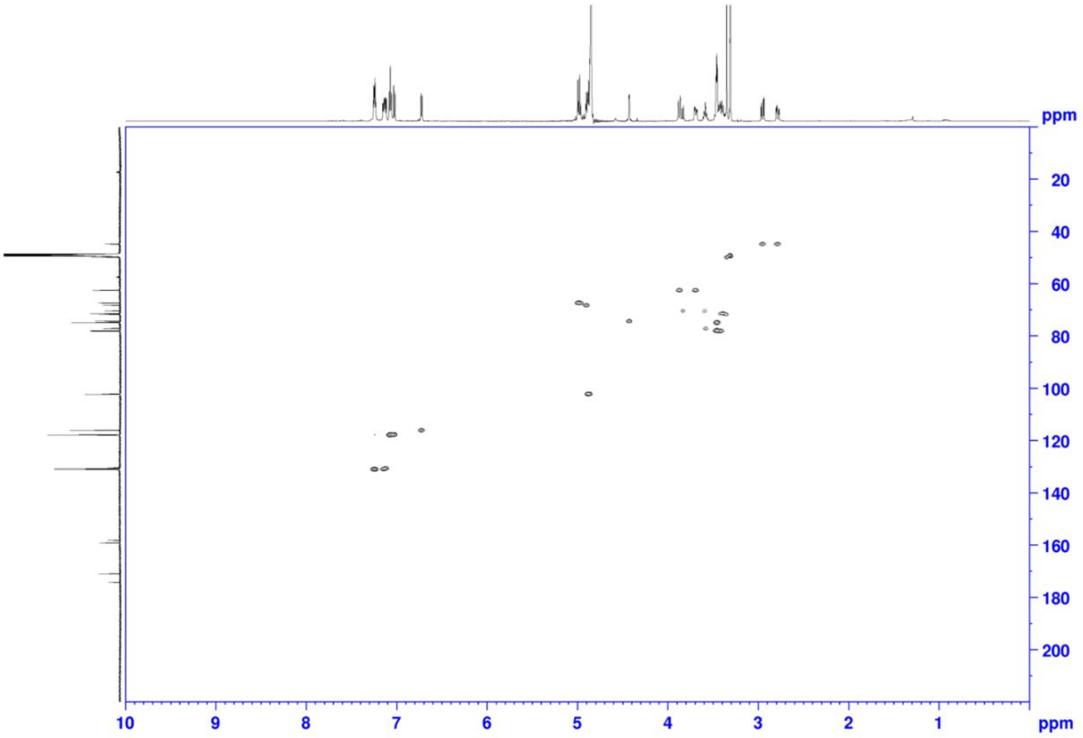

Figure S 30 HSQC NMR spectrum of compound **3** in MeOD.

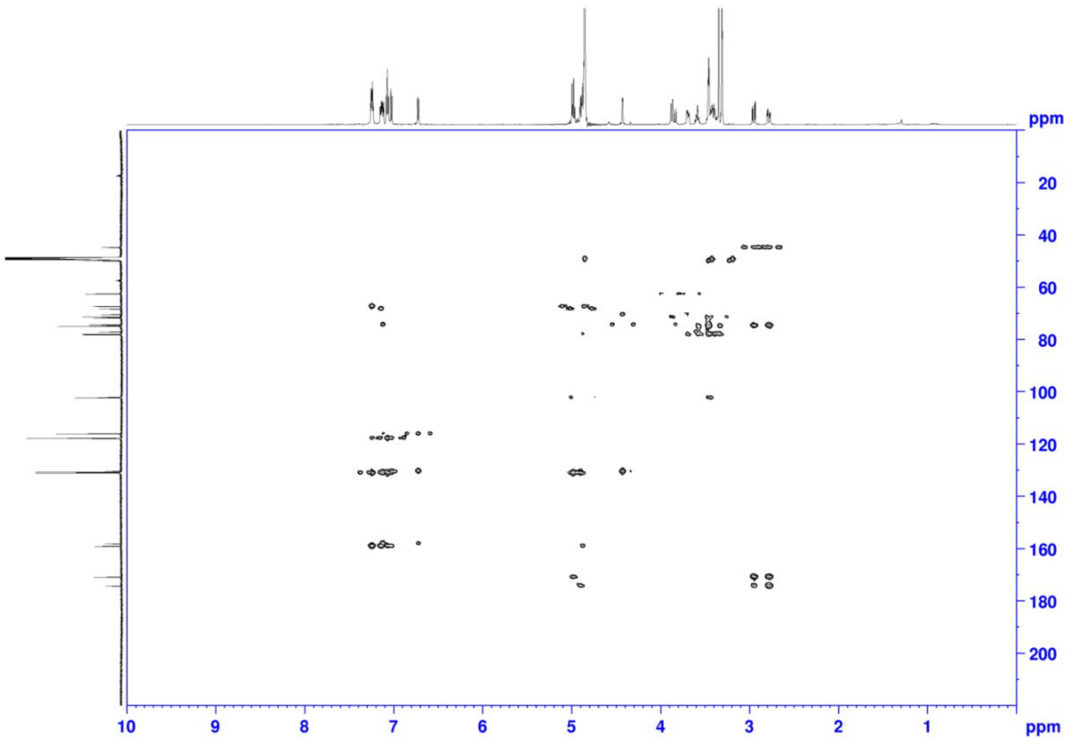

Figure S 31 HMBC NMR spectrum of compound **3** in MeOD.

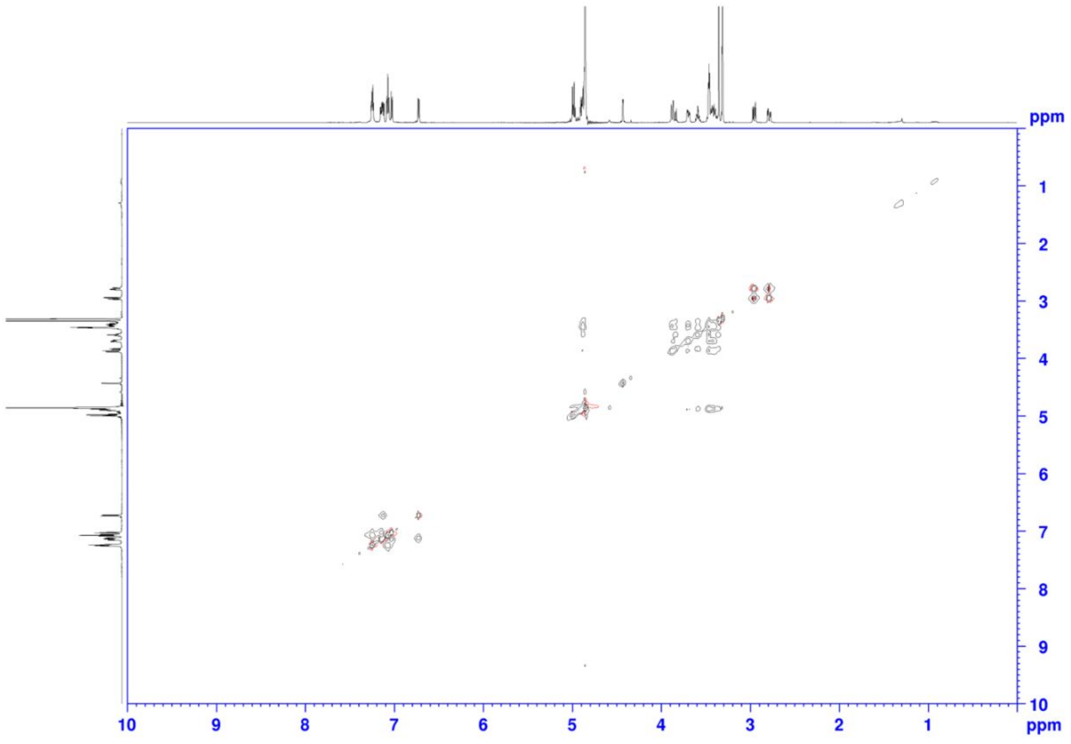

Figure S 32 TOCSY NMR spectrum of compound **3** in MeOD.

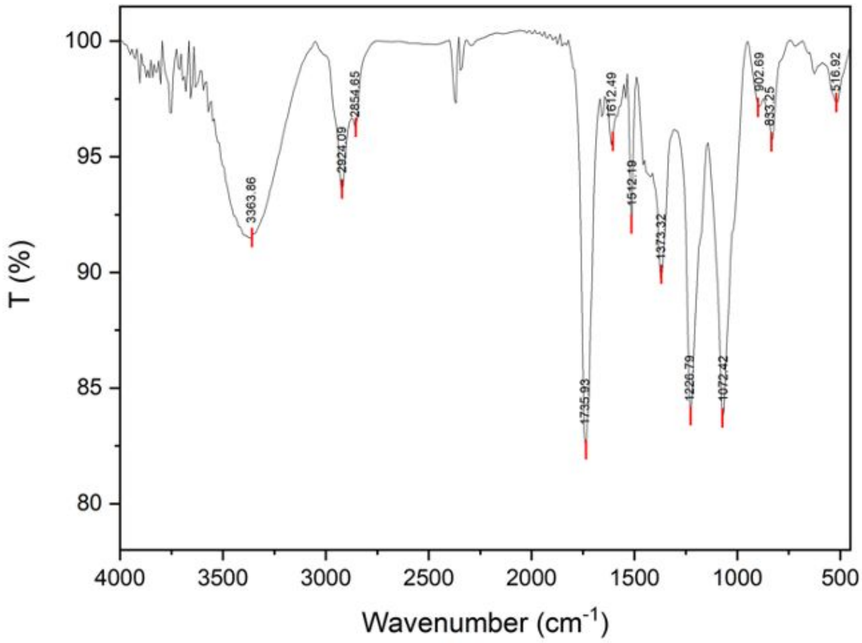

Figure S 33 IR spectrum of compound **3** (KBr).

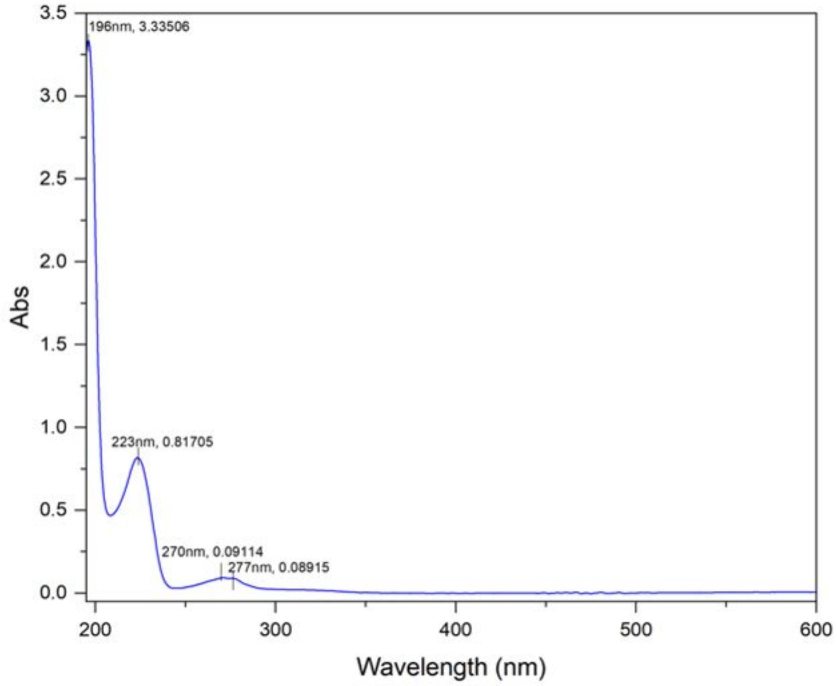

Figure S 34 UV spectrum of compound **3** in MeOH.

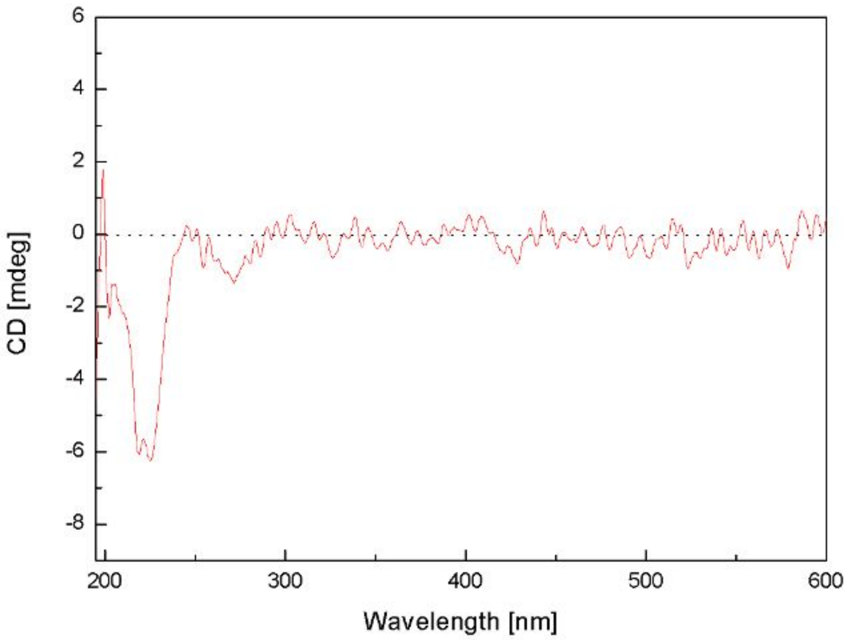

Figure S 35 CD spectrum of compound **3** in MeOH.

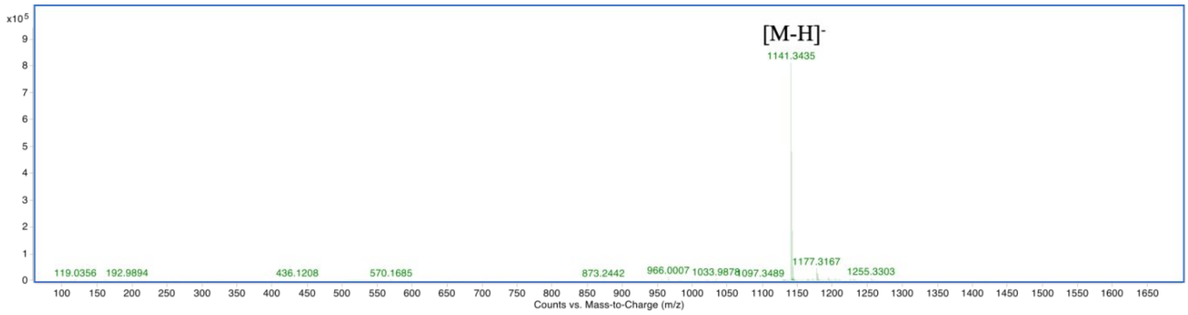

Formula (M)	Score (MFG)	m/z	m/z (calcd.)	Error (ppm)	DBE
$C_{54}H_{62}O_{27}$	95.71	1141.3435	1141.3406	-2.18	24

Figure S 36 HR-ESI-MS spectrum of compound **4**.

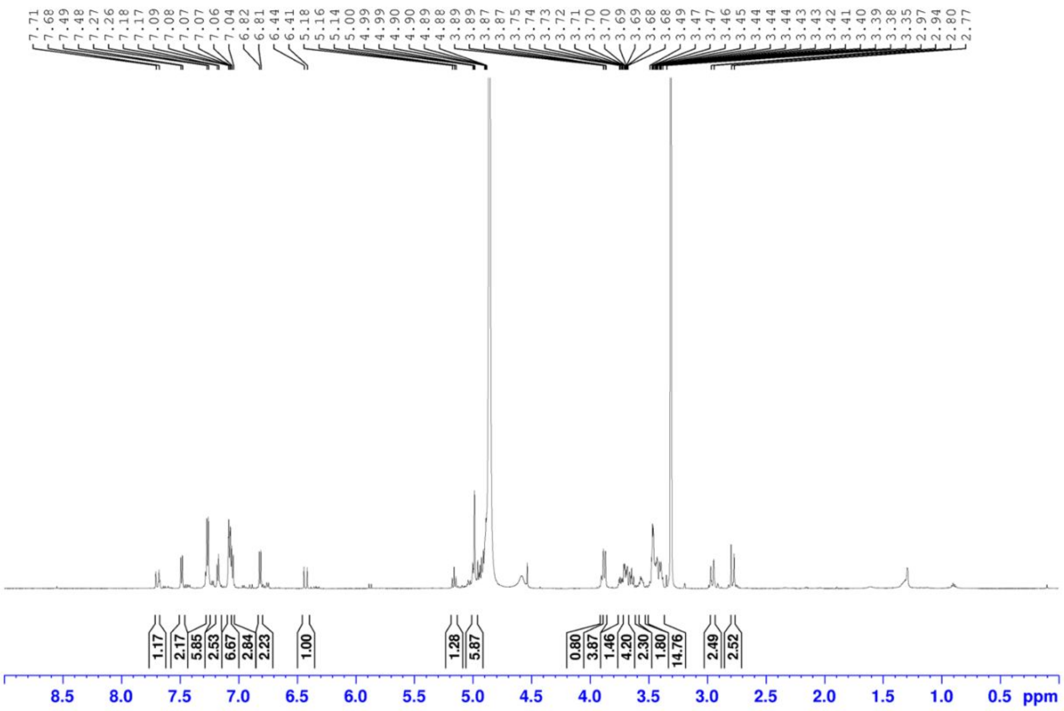

Figure S 37 ^{1}H NMR spectrum of compound **4** in MeOD.

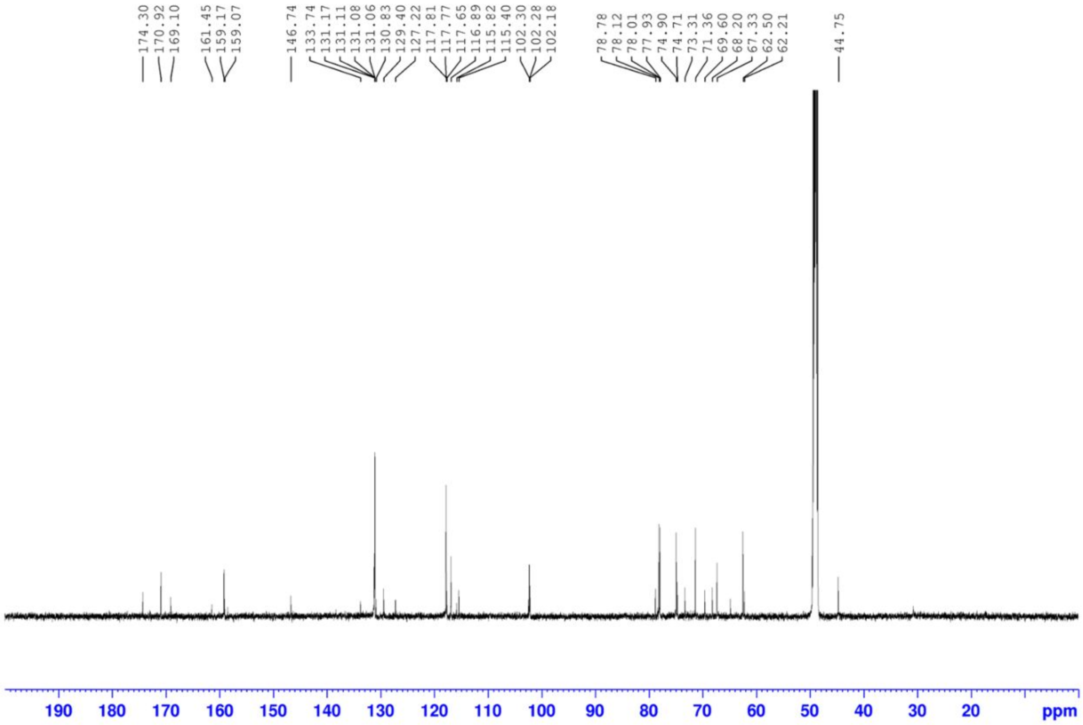

Figure S 38 ^{13}C NMR spectrum of compound **4** in MeOD.

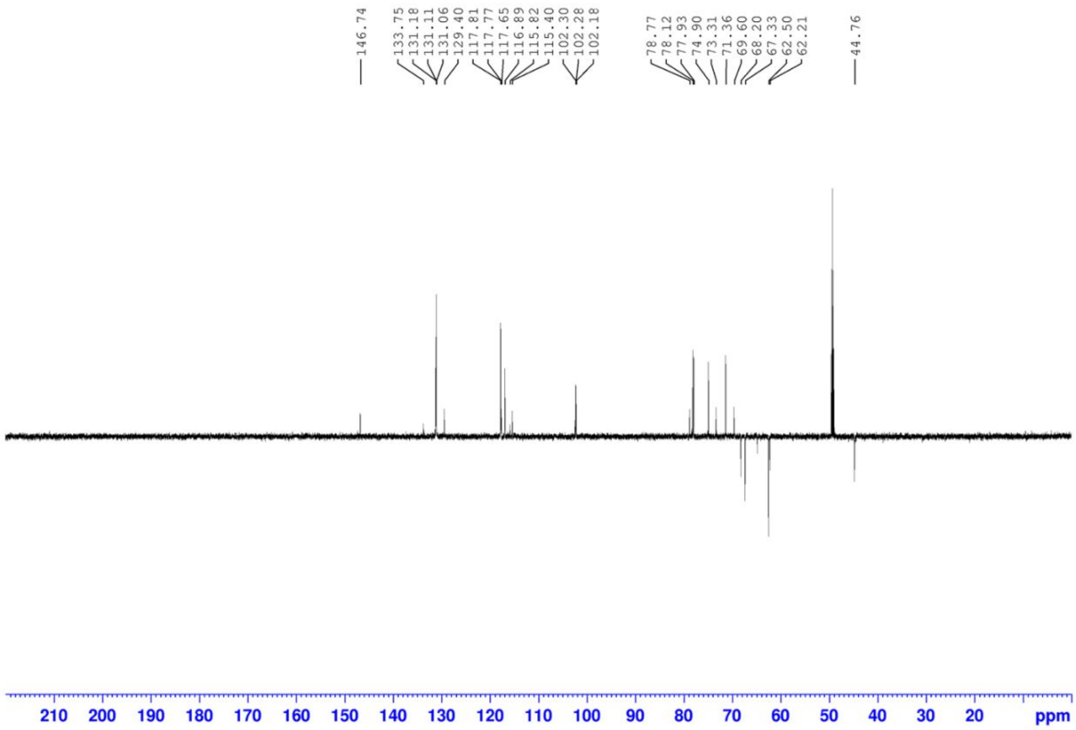

Figure S 39 DEPT135 NMR spectrum of compound **4** in MeOD.

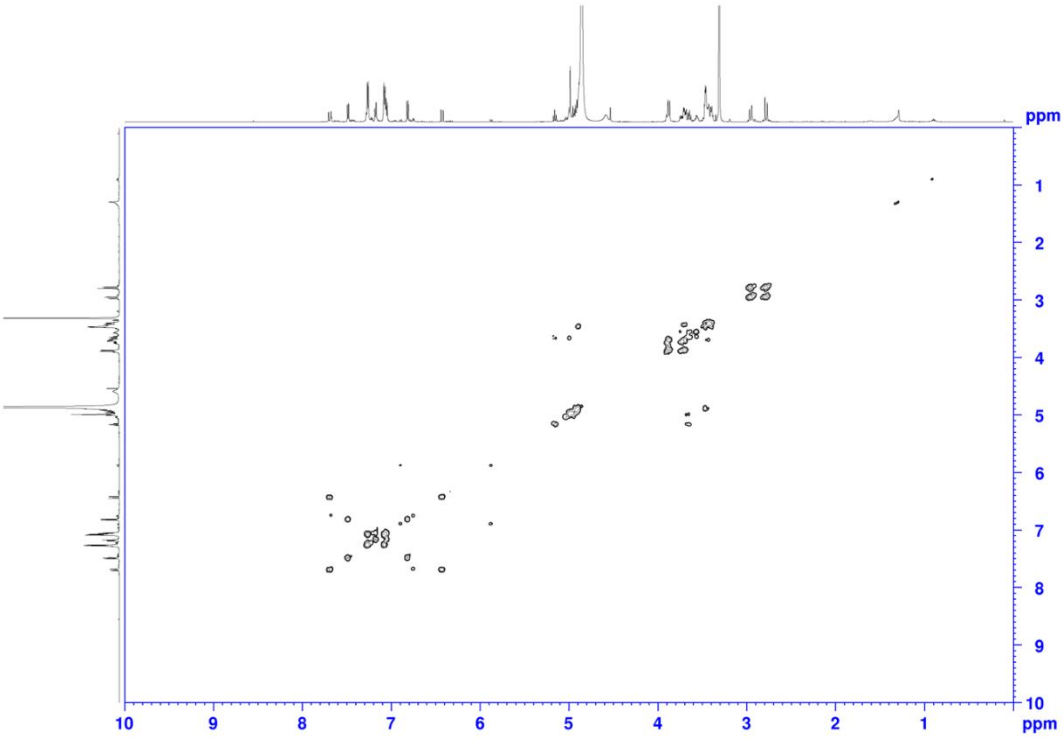

Figure S 40 ^{1}H–^{1}H COSY NMR spectrum of compound **4** in MeOD.

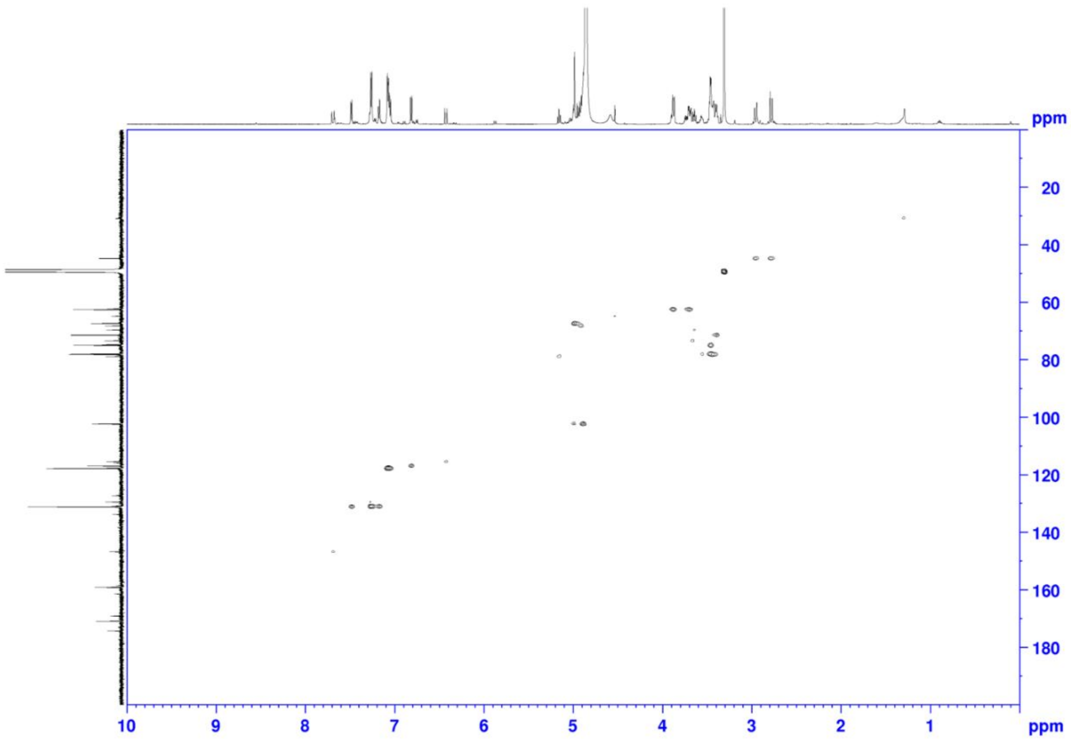

Figure S 41 HSQC NMR spectrum of compound **4** in MeOD.

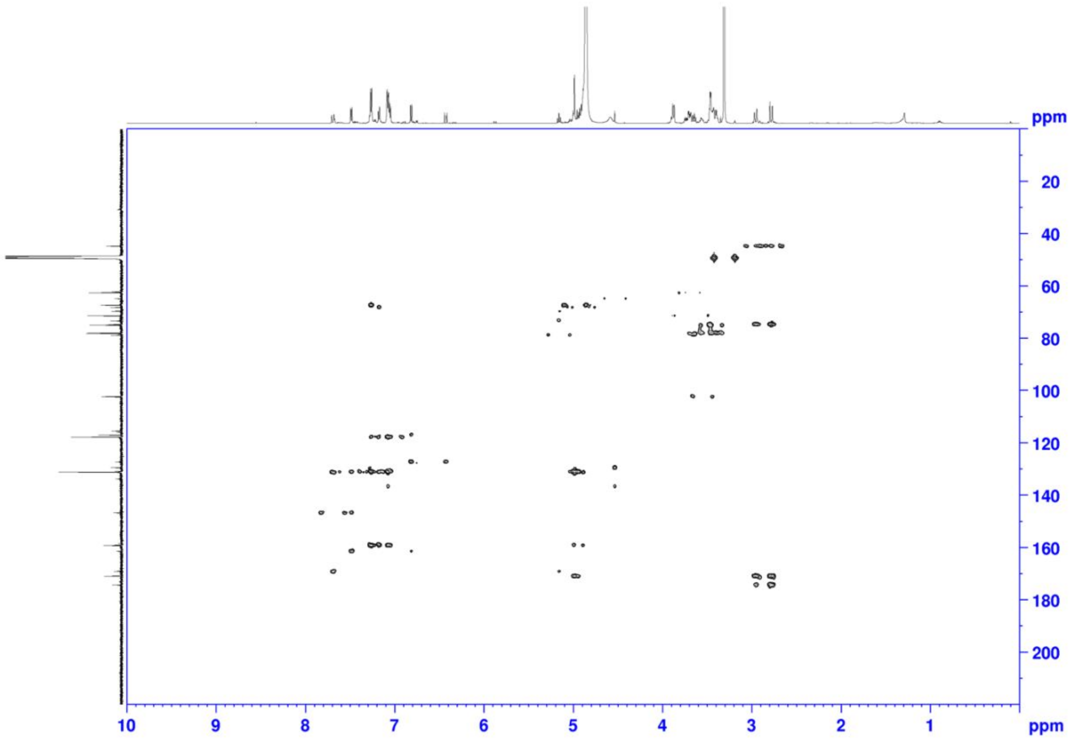

Figure S 42 HMBC NMR spectrum of compound **4** in MeOD.

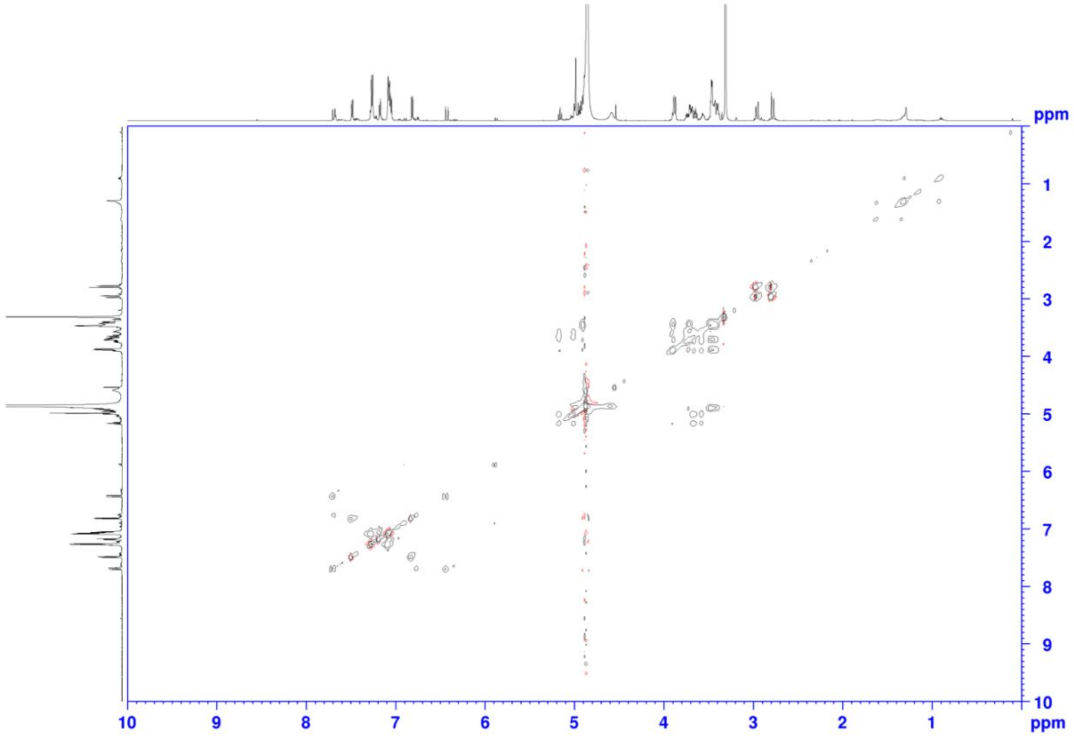

Figure S 43 TOCSY NMR spectrum of compound **4** in MeOD.

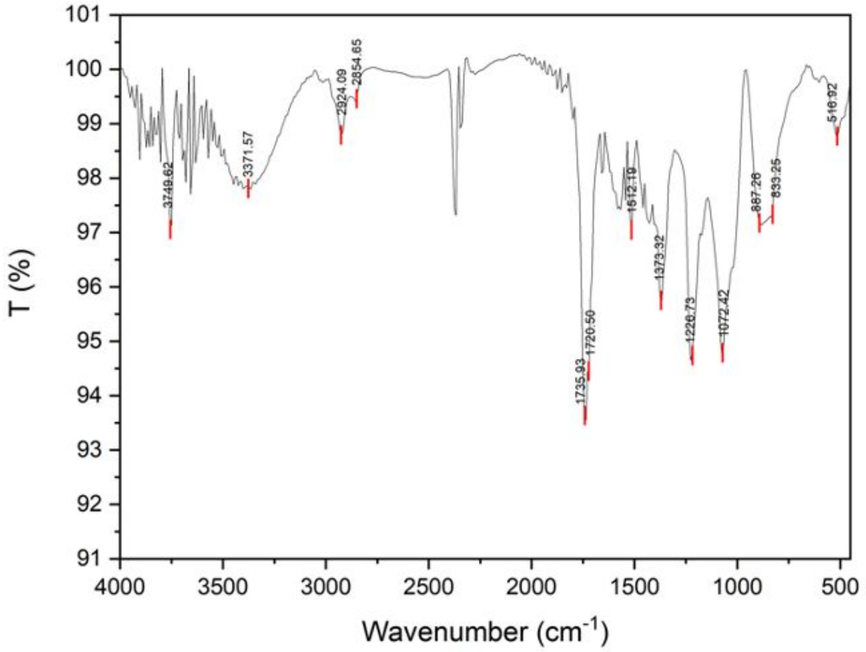

Figure S 44 IR spectrum of compound **4** (KBr).

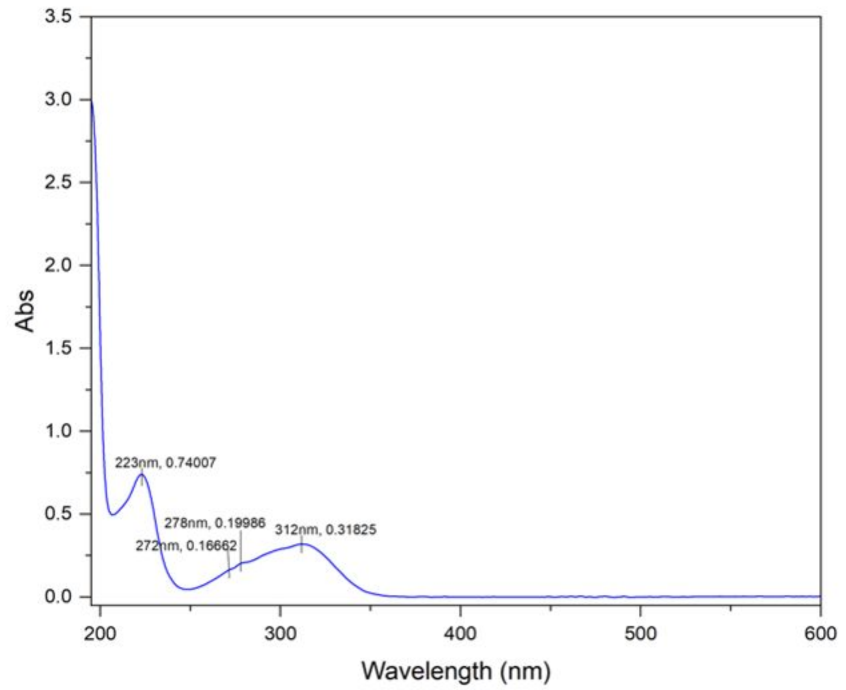

Figure S 45 UV spectrum of compound **4** in MeOH.

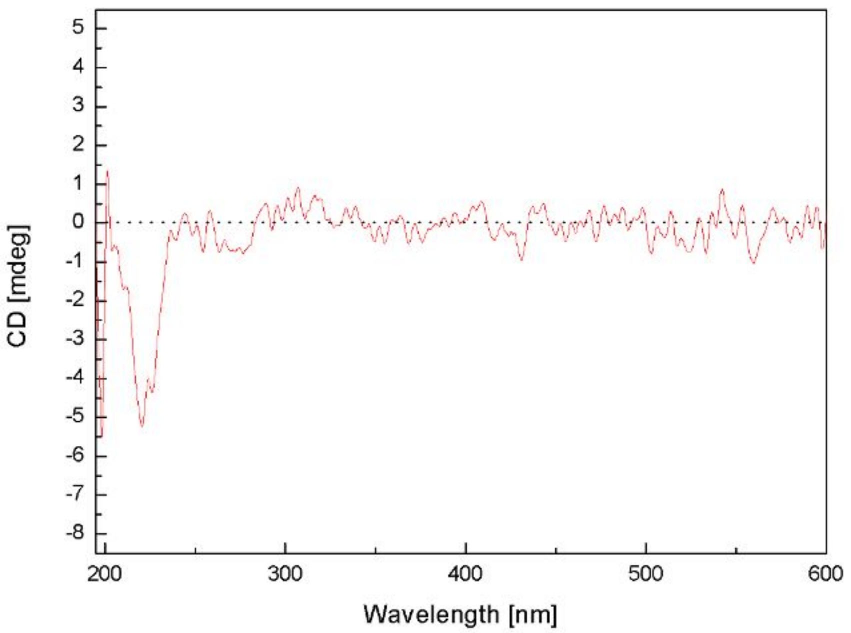

Figure S 46 CD spectrum of compound **4** in MeOH.

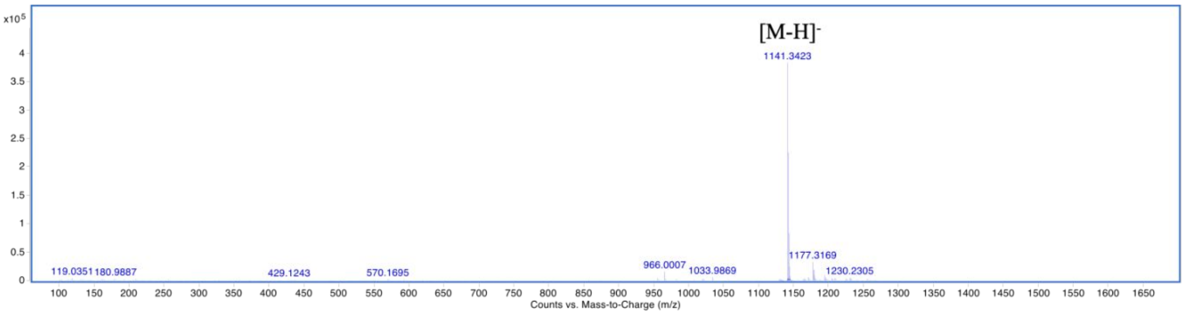

Formula (M)	Score (MFG)	m/z	m/z (calcd.)	Error (ppm)	DBE
$C_{54}H_{62}O_{27}$	98.05	1141.3423	1141.3406	-1.18	24

Figure S 47 HR-ESI-MS spectrum of compound **5**.

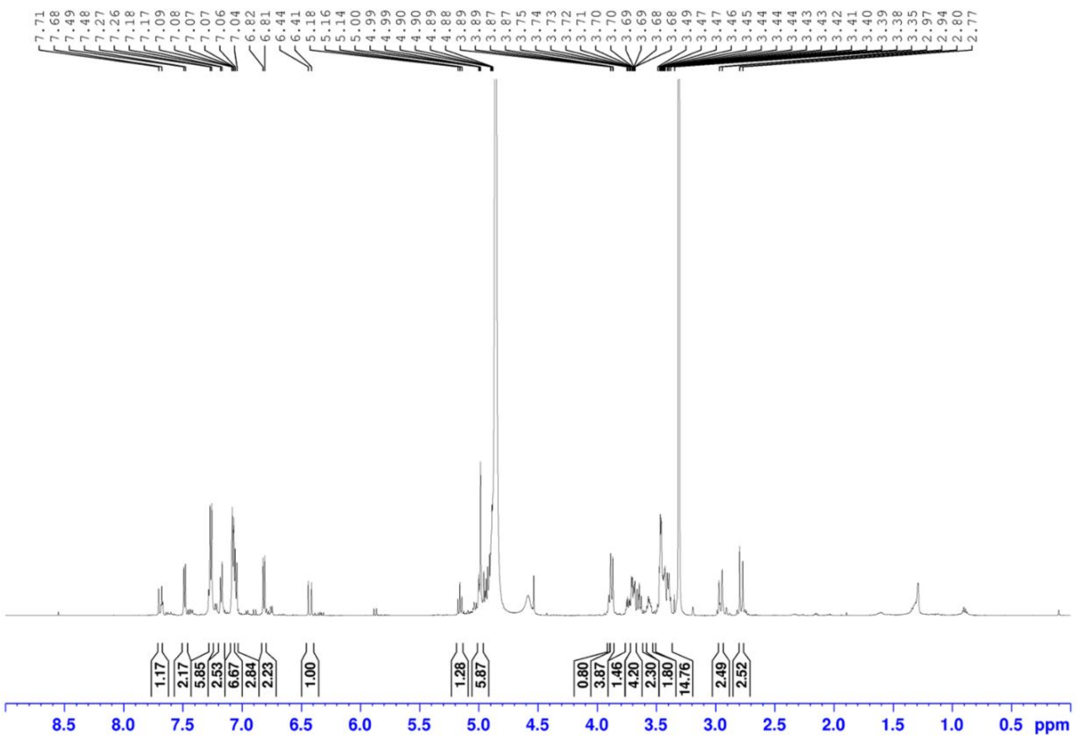

Figure S 48 ^{1}H NMR spectrum of compound **5** in MeOD.

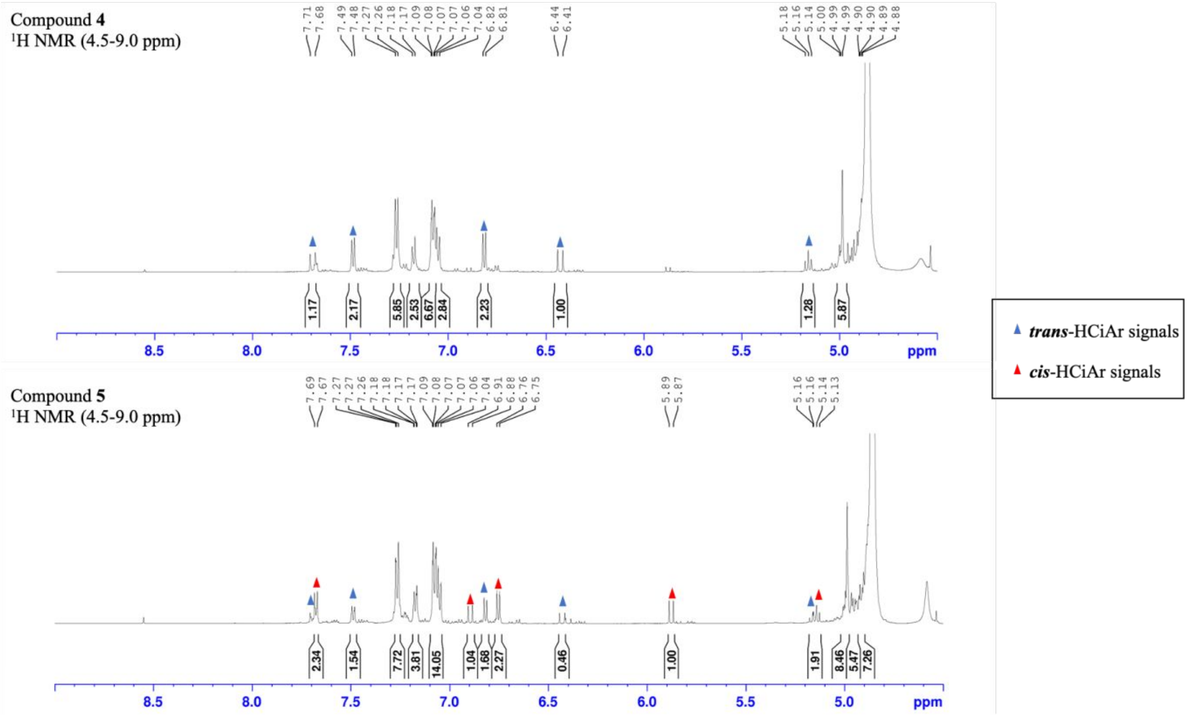

Figure S 49 Comparison of ^{1}H NMR spectra between compound **4** and compound **5**

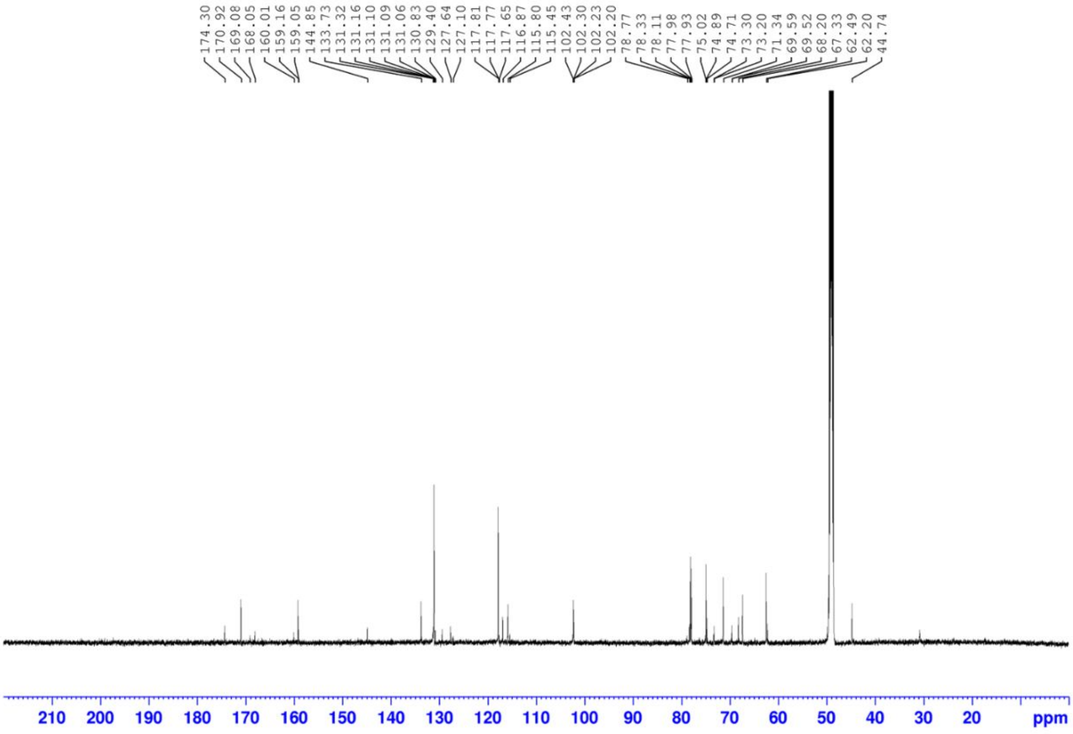

Figure S 50 ^{13}C NMR spectrum of compound **5** in MeOD.

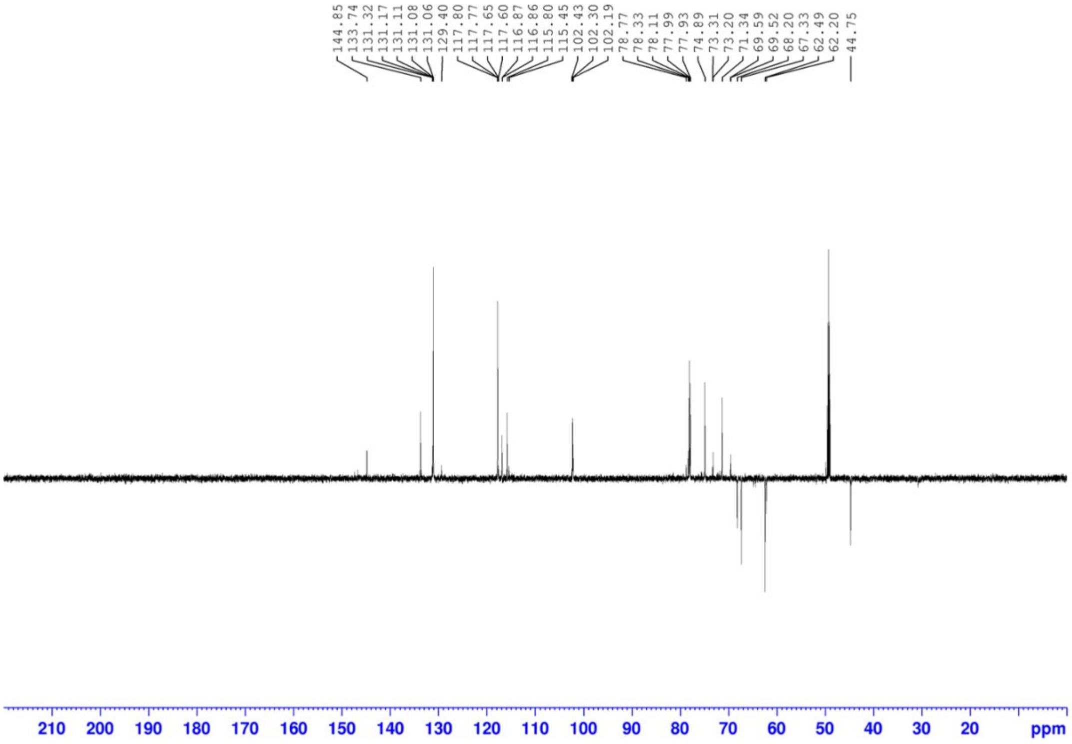

Figure S 51 DEPT135 NMR spectrum of compound **5** in MeOD.

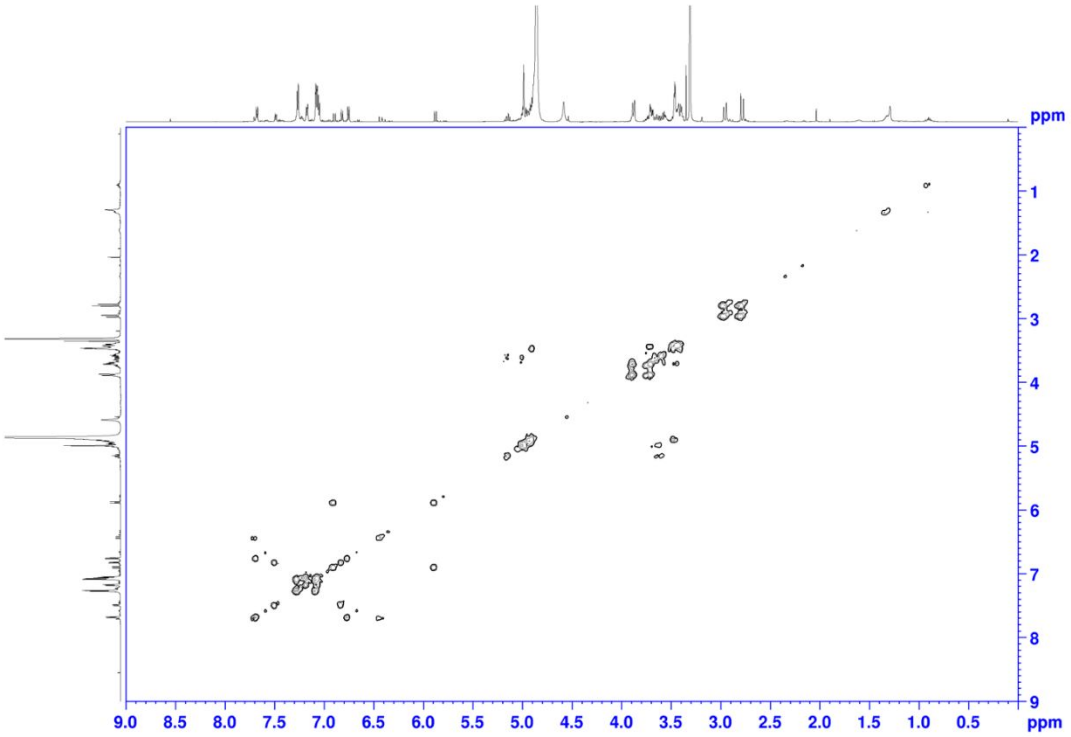

Figure S 52 ^{1}H–^{1}H COSY NMR spectrum of compound **5** in MeOD.

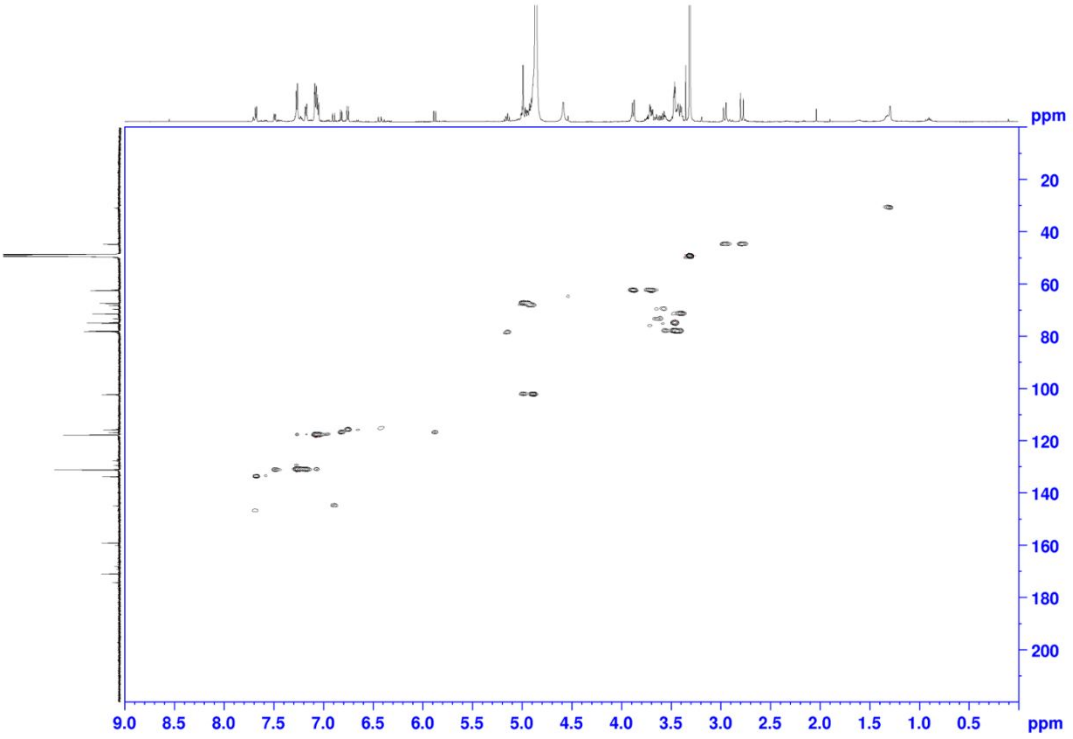

Figure S 53 HSQC NMR spectrum of compound **5** in MeOD.

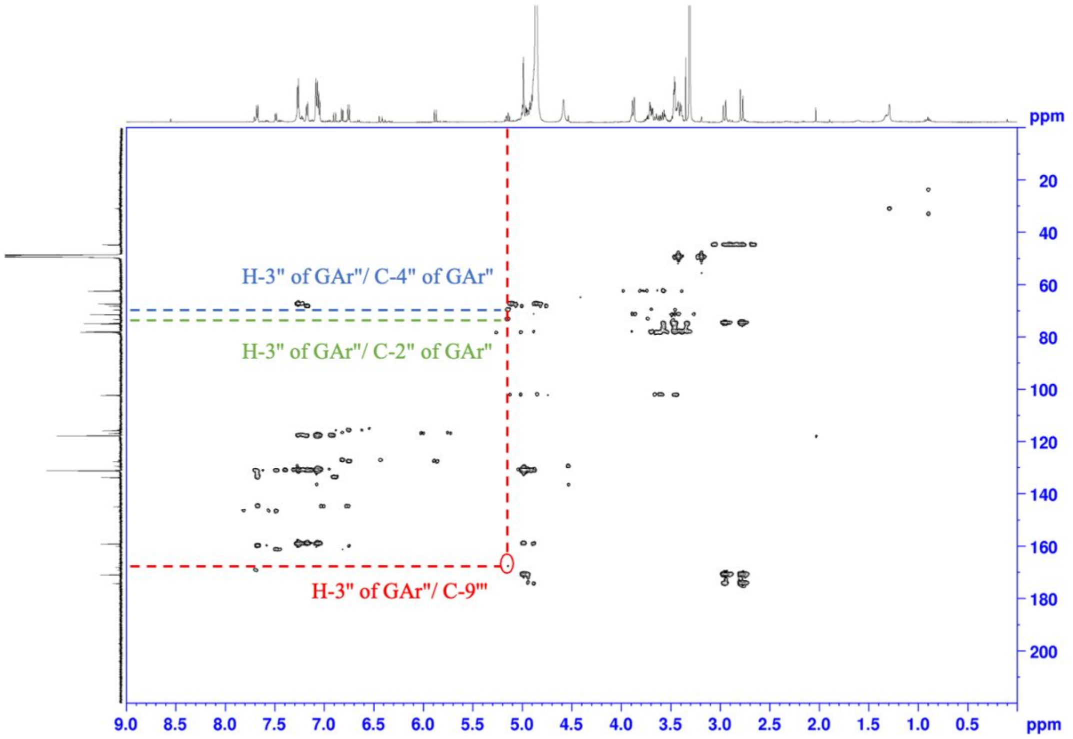

Figure S 54 HMBC NMR spectrum of compound **5** in MeOD.

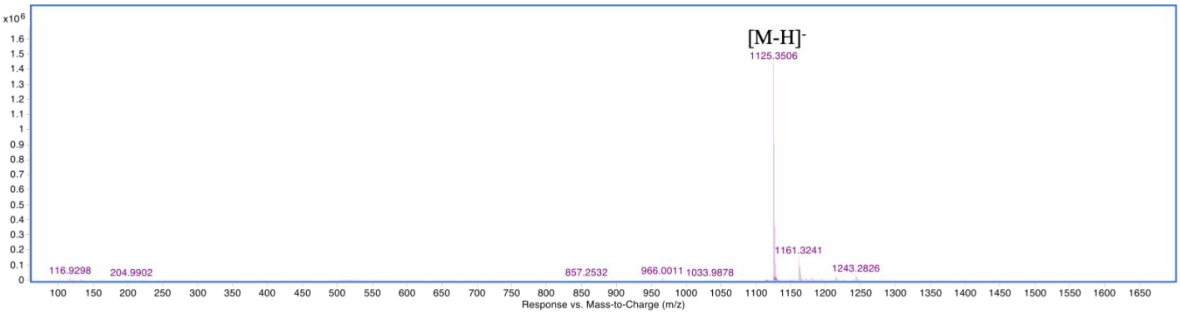

Formula (M)	Score (MFG)	*m/z*	*m/z* (calcd.)	Error (ppm)	DBE
$C_{54}H_{62}O_{26}$	85.67	1125.3506	1125.3457	-4.26	24

Figure S 55 HR-ESI-MS spectrum of compound **6**.

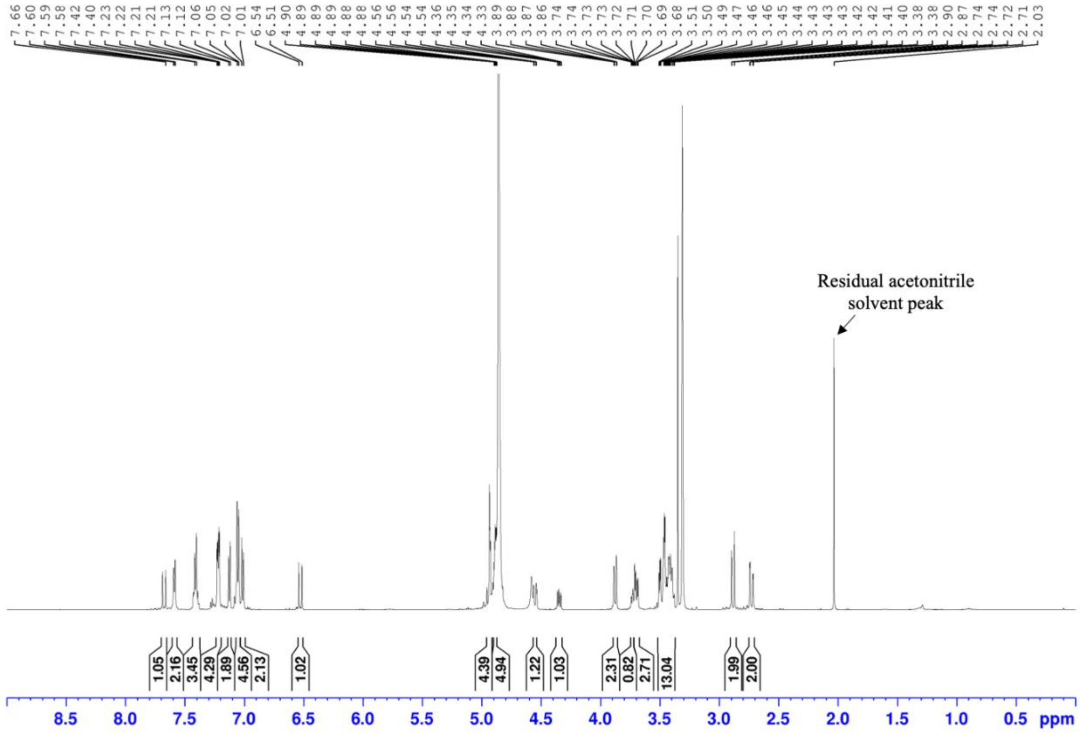

Figure S 56 ^{1}H NMR spectrum of compound **6** in MeOD.

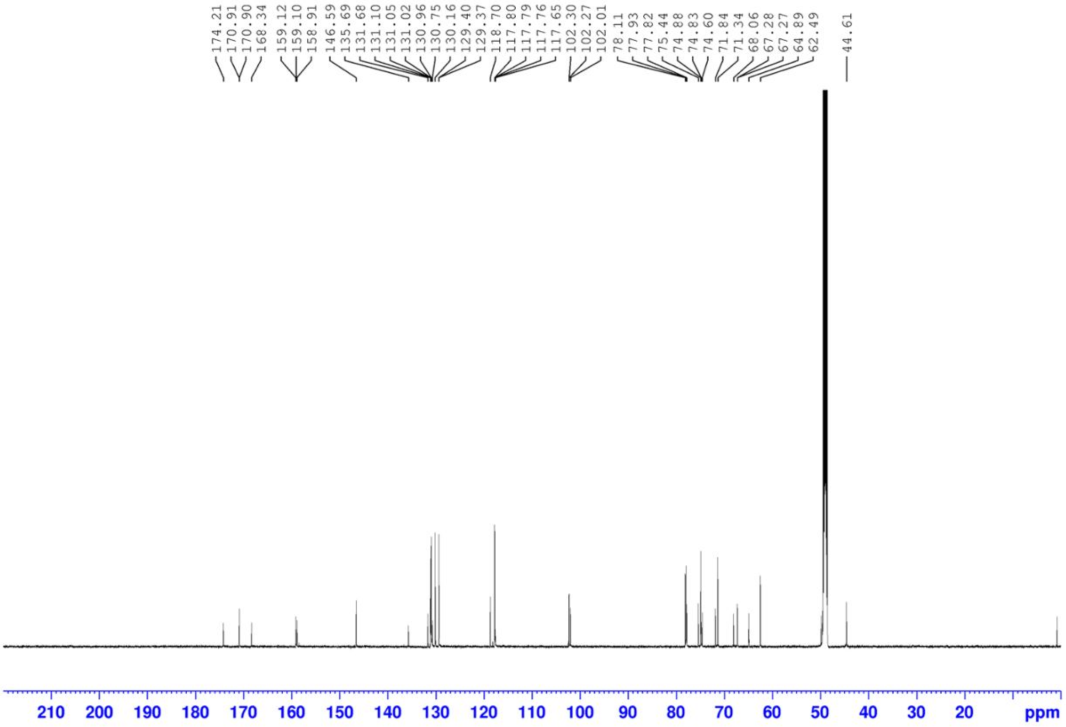

Figure S 57 ^{13}C NMR spectrum of compound **6** in MeOD.

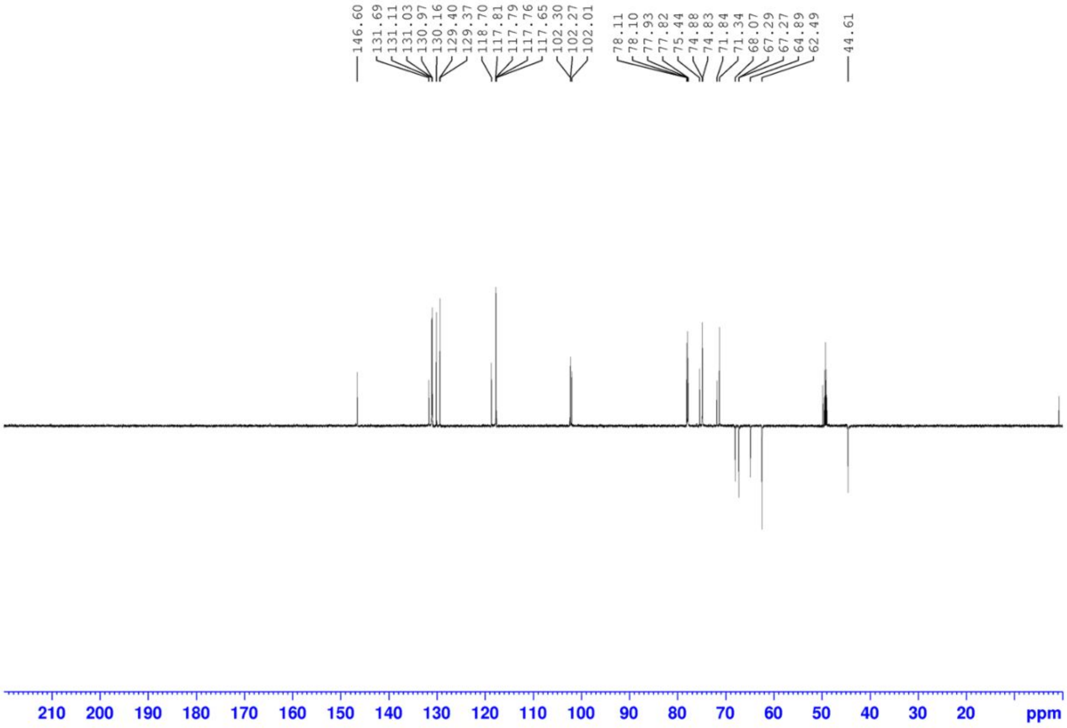

Figure S 58 DEPT135 NMR spectrum of compound **6** in MeOD.

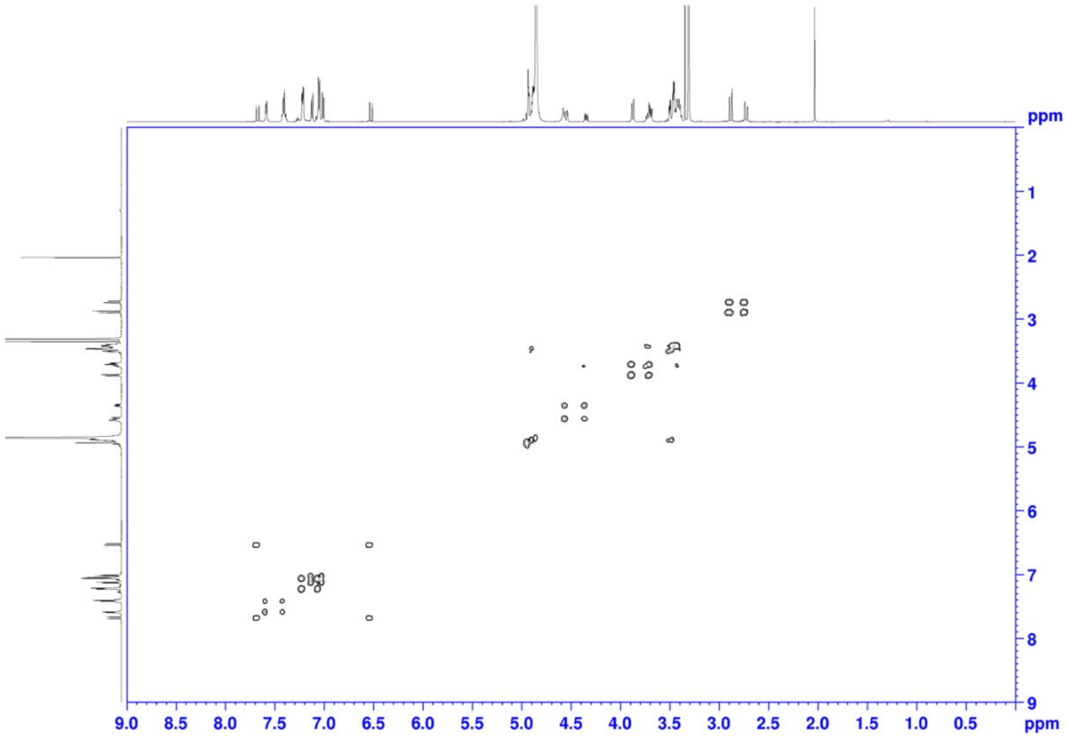

Figure S 59 ^{1}H–^{1}H COSY NMR spectrum of compound **6** in MeOD.

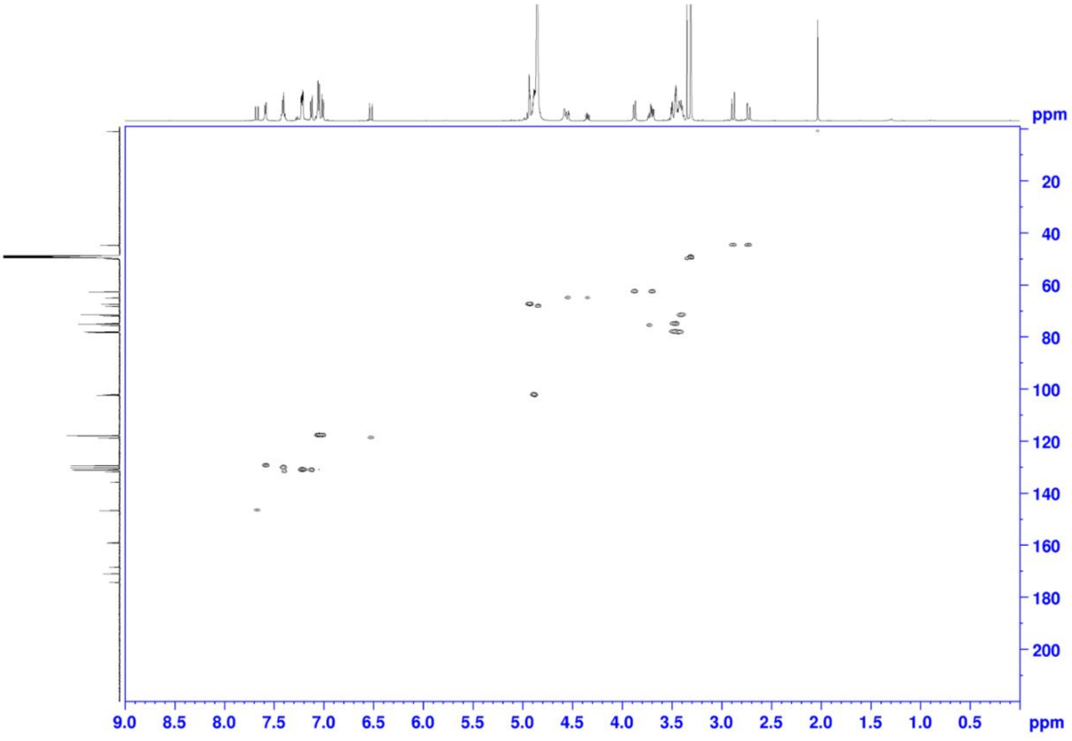

Figure S 60 HSQC NMR spectrum of compound **6** in MeOD.

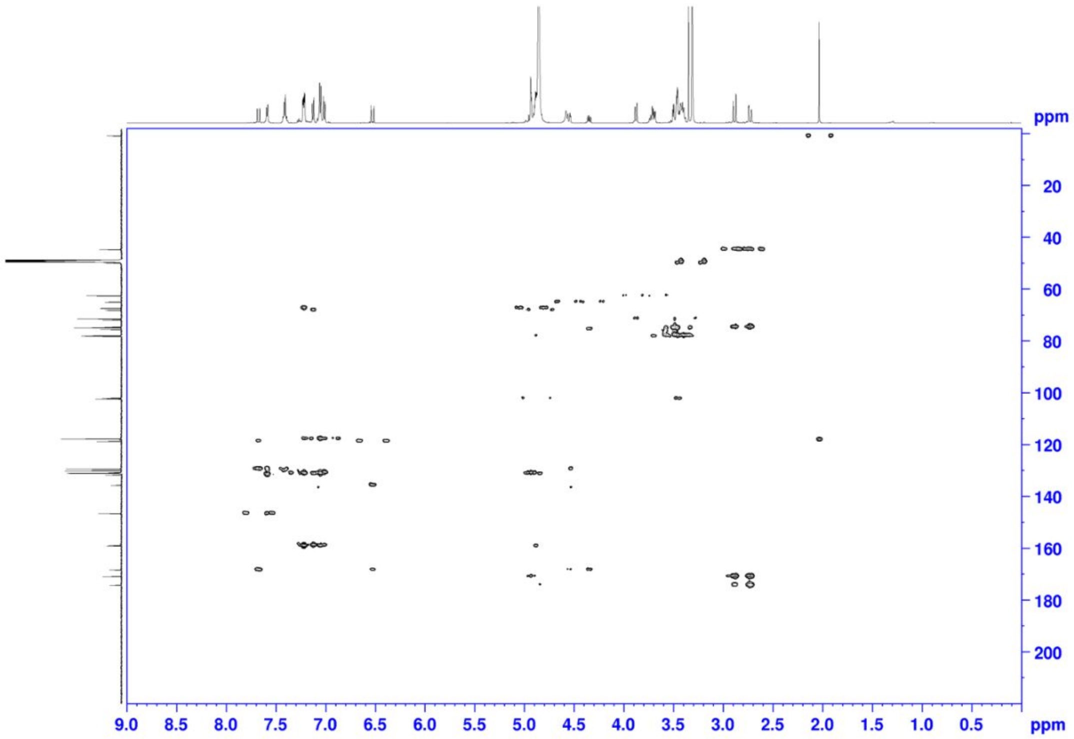

Figure S 61 HMBC NMR spectrum of compound **6** in MeOD.

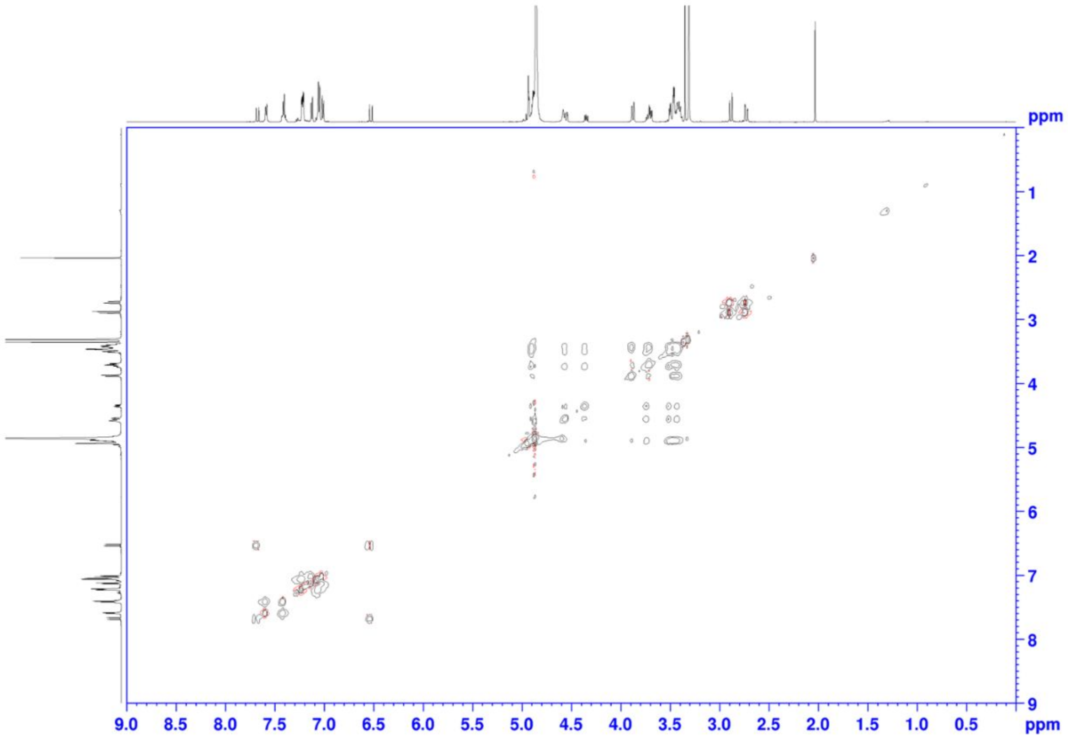

Figure S 62 TOCSY NMR spectrum of compound **6** in MeOD.

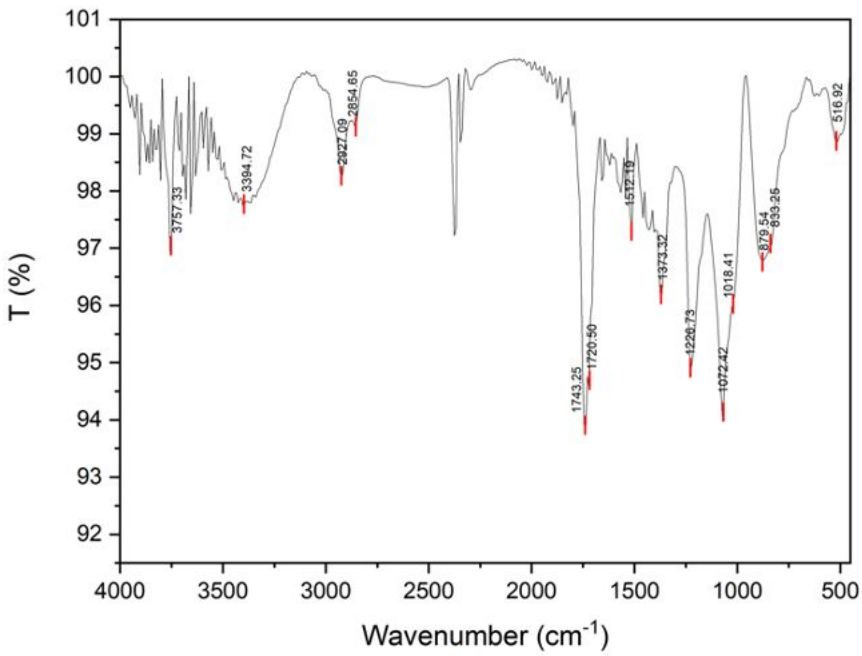

Figure S 63 IR spectrum of compound **6** (KBr).

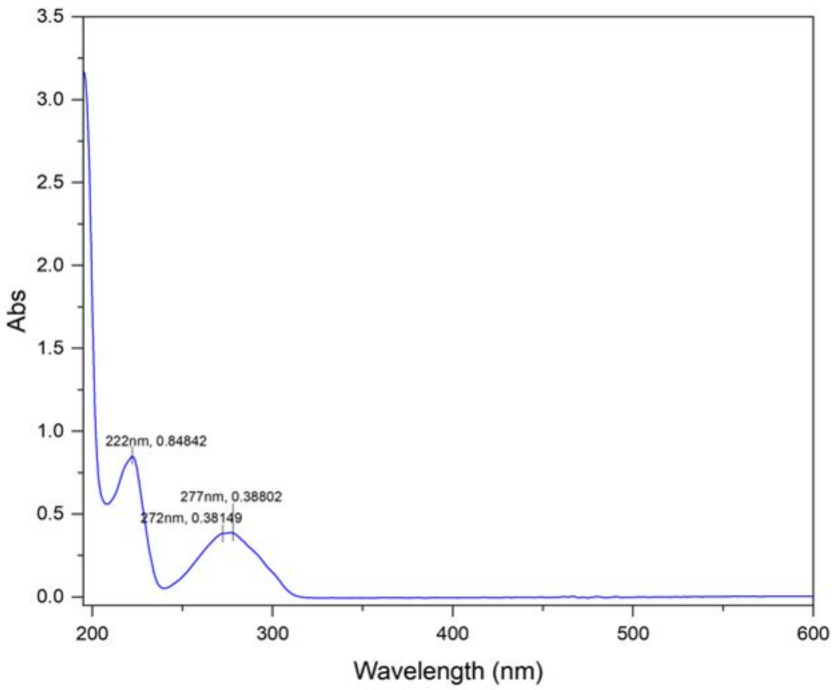

Figure S 64 UV spectrum of compound **6** in MeOH.

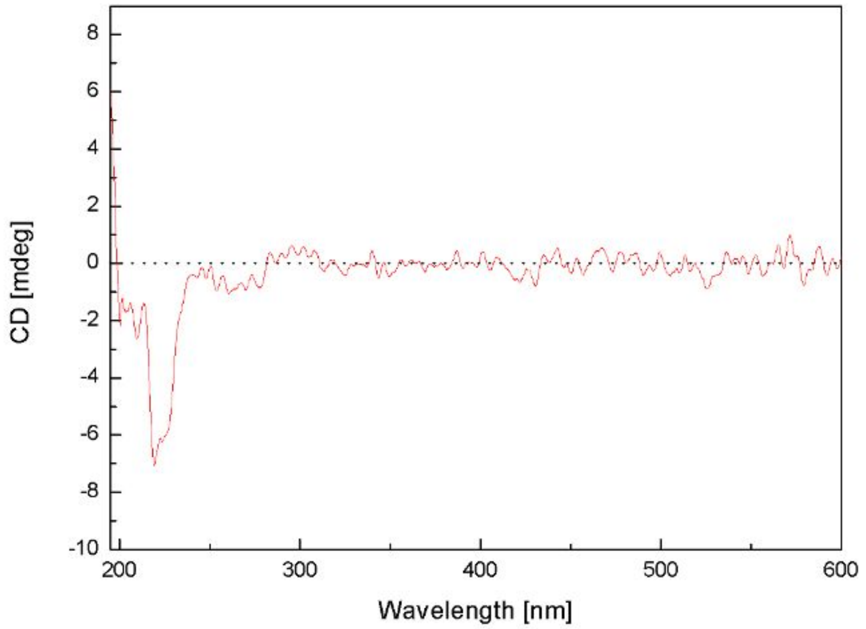

Figure S 65 CD spectrum of compound **6** in MeOH.

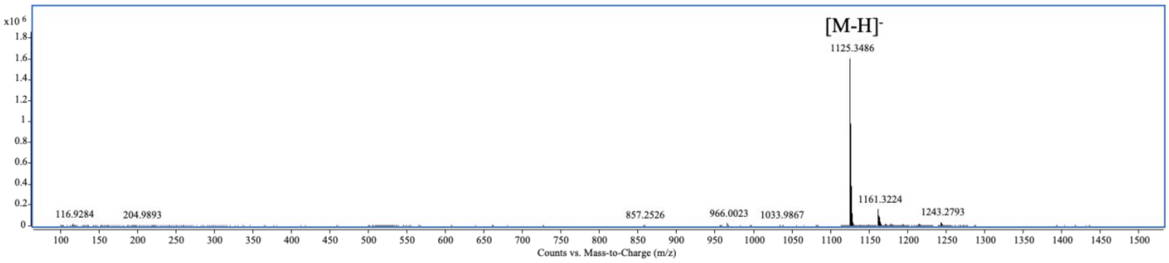

Formula (M)	Score (MFG)	*m/z*	*m/z* (calcd.)	Error (ppm)	DBE
$C_{54}H_{62}O_{26}$	94.38	1125.3486	1125.3457	-2.61	24

Figure S 66 HR-ESI-MS spectrum of compound **7**.

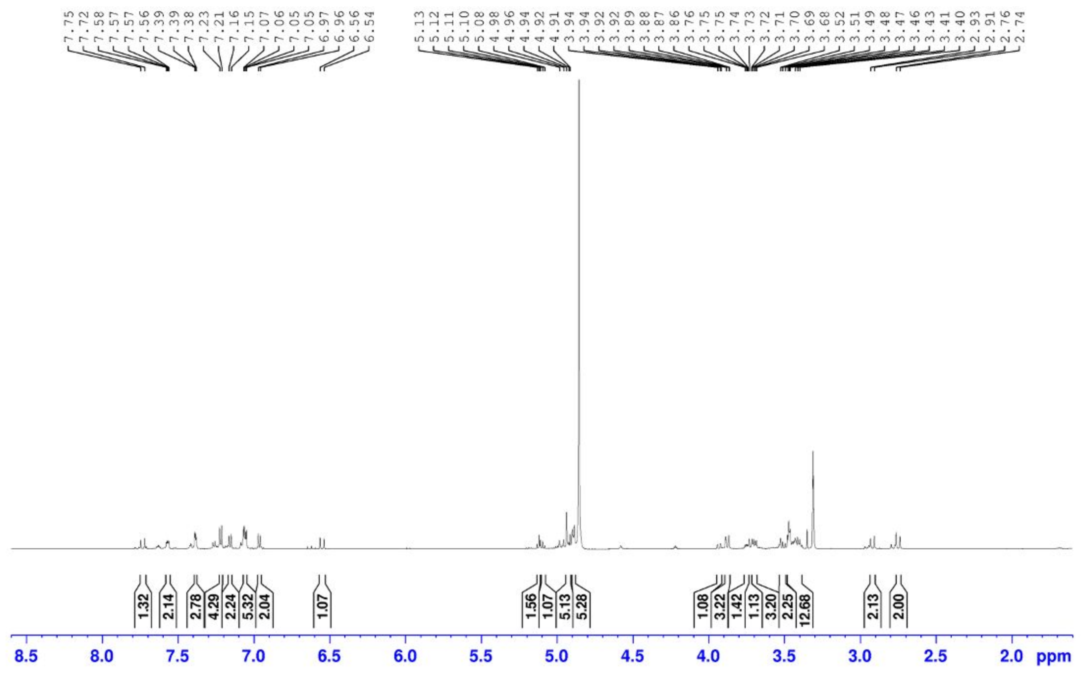

Figure S 67 ^{1}H NMR spectrum of compound **7** in MeOD.

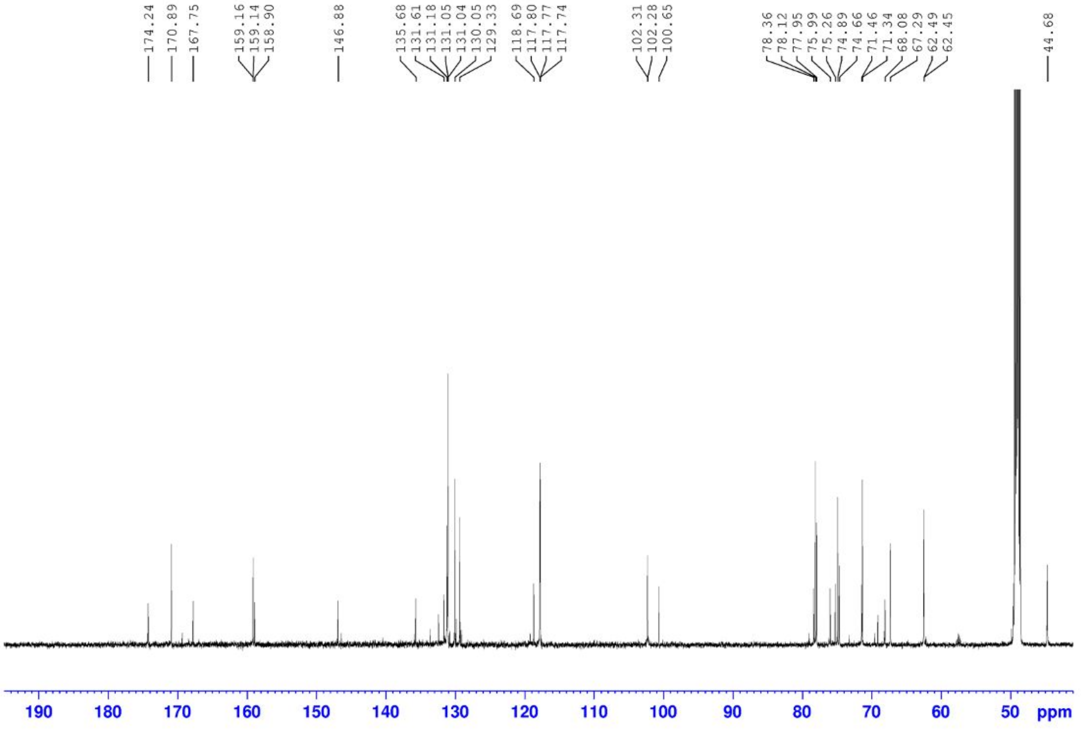

Figure S 68 ^{13}C NMR spectrum of compound **7** in MeOD.

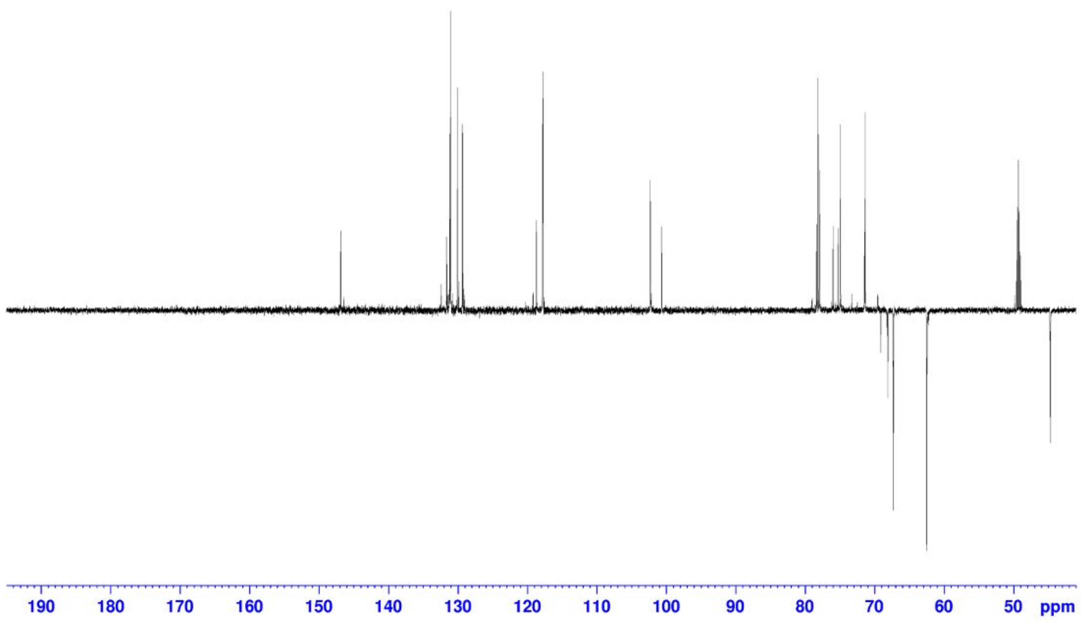

Figure S 69 DEPT135 NMR spectrum of compound **7** in MeOD.

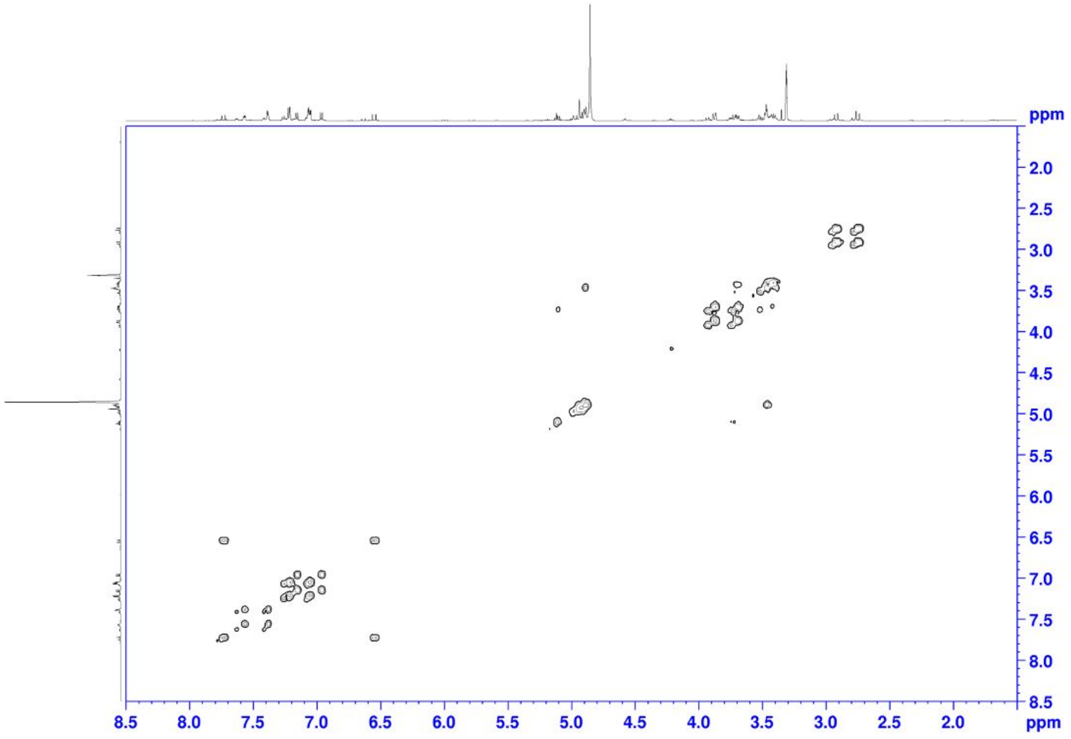

Figure S 70 ^{1}H–^{1}H COSY NMR spectrum of compound **7** in MeOD.

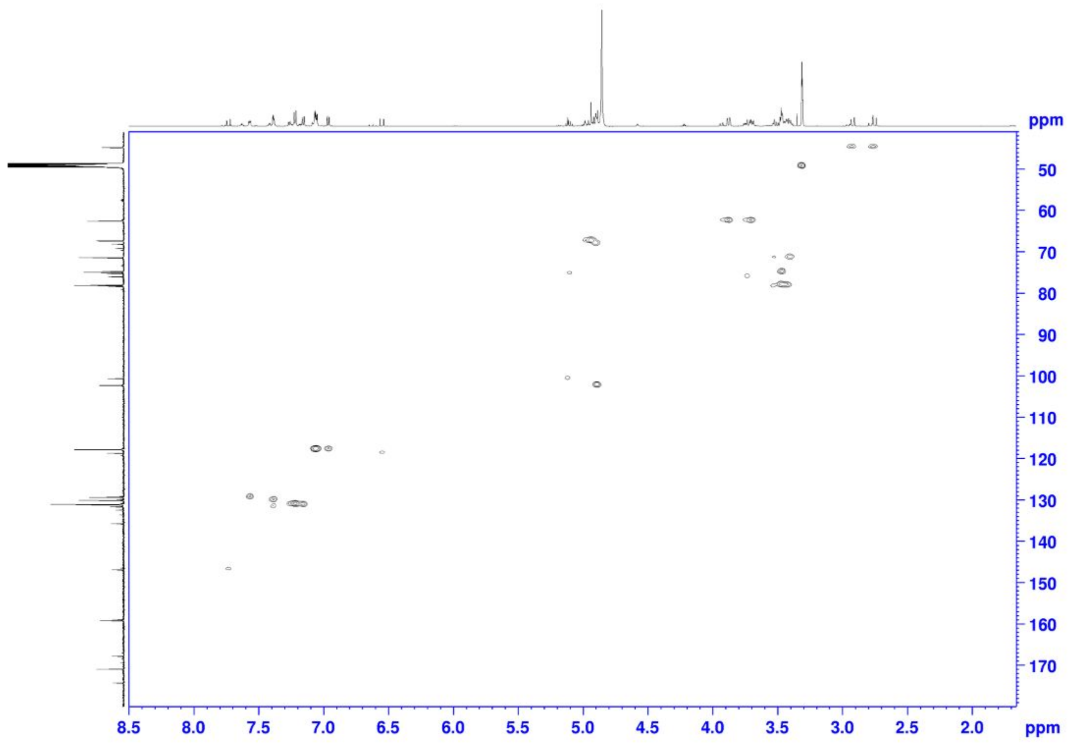

Figure S 71 HSQC NMR spectrum of compound **7** in MeOD.

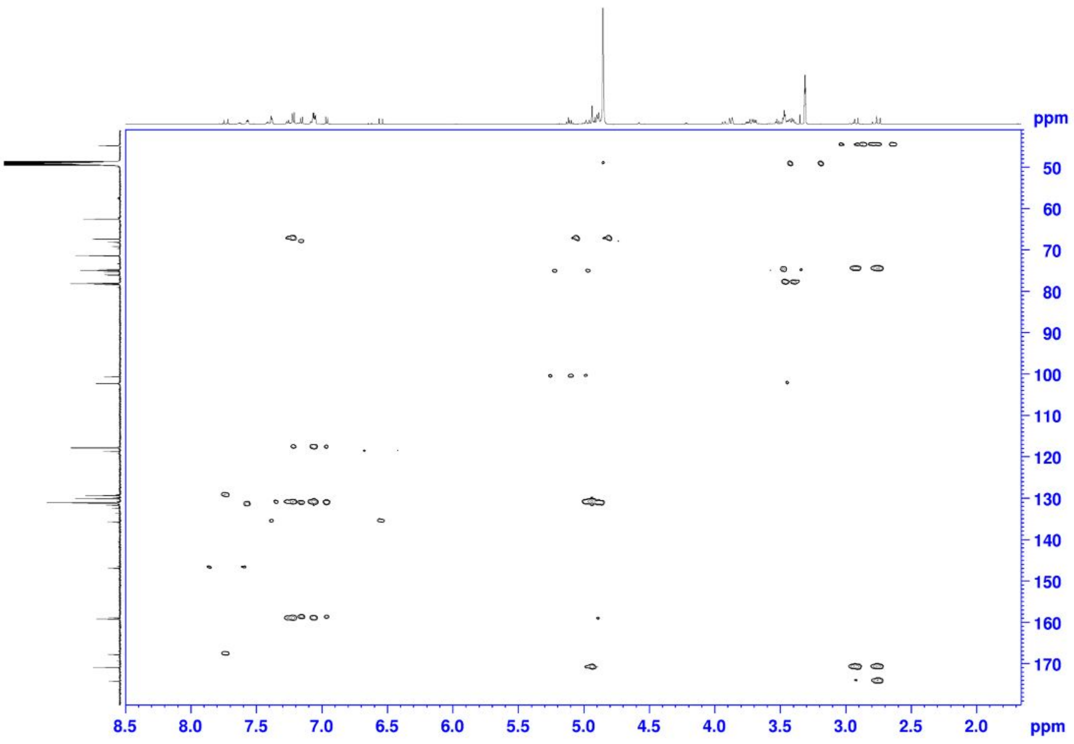

Figure S 72 HMBC NMR spectrum of compound **7** in MeOD.

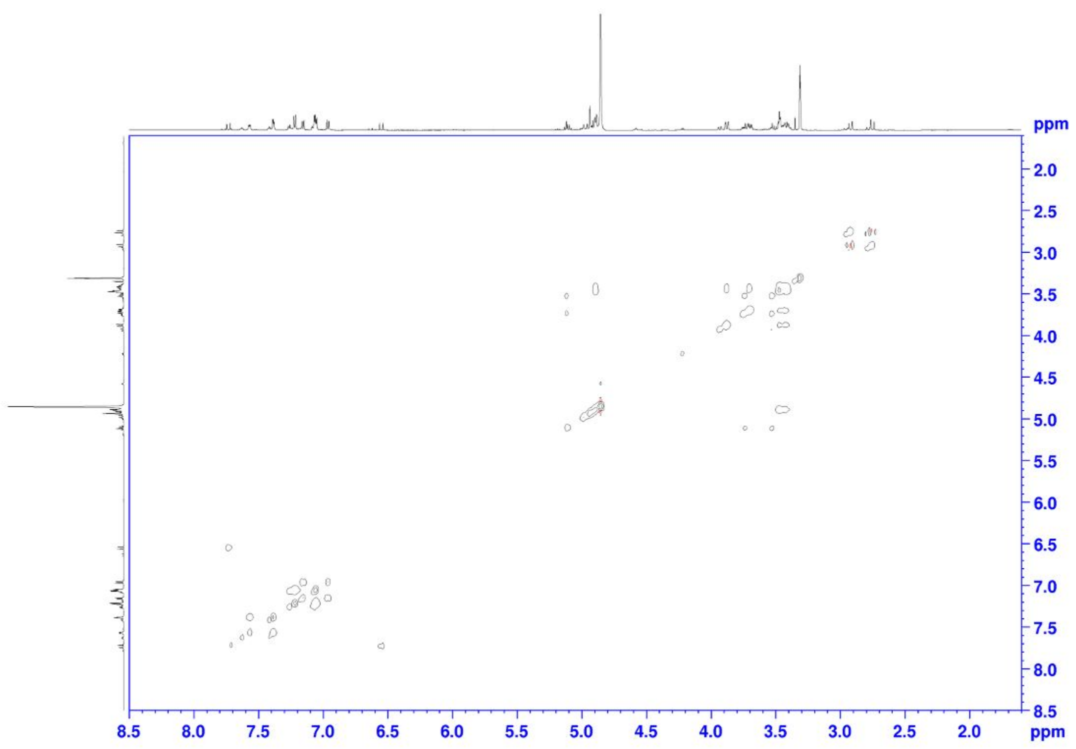

Figure S 73 TOCSY NMR spectrum of compound **7** in MeOD.

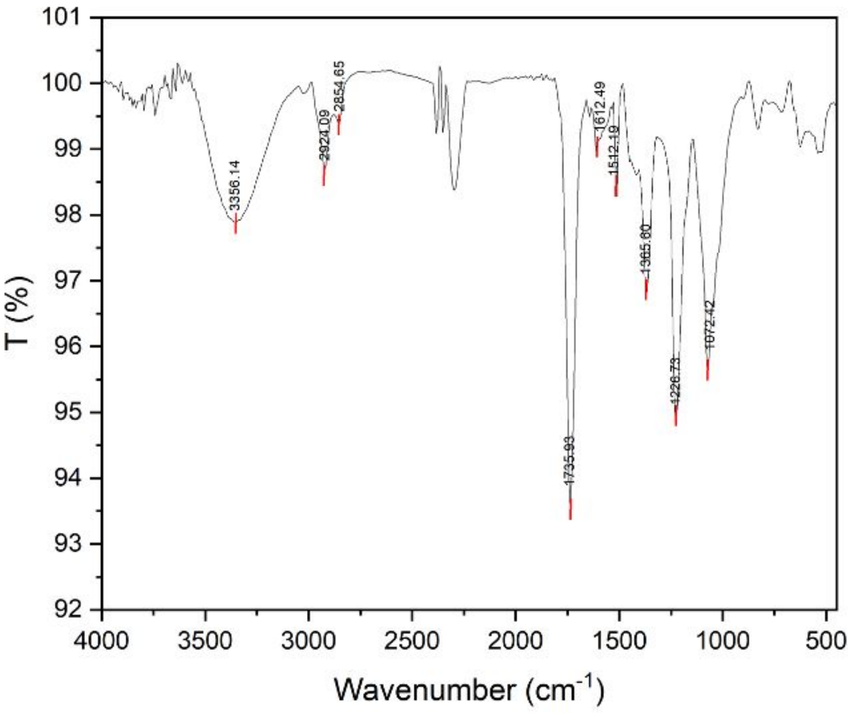

Figure S 74 IR spectrum of compound **7** (KBr).

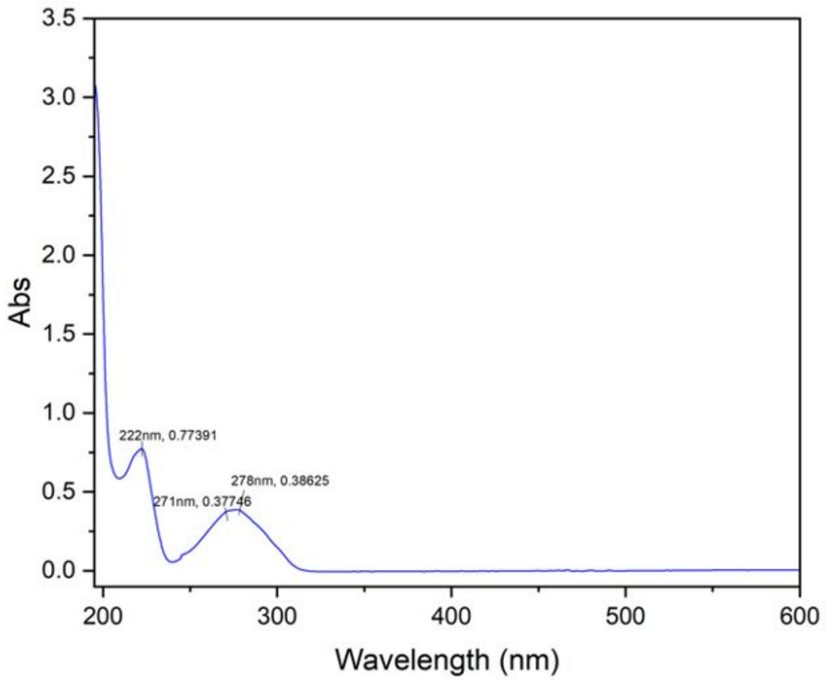

Figure S 75 UV spectrum of compound **7** in MeOH.

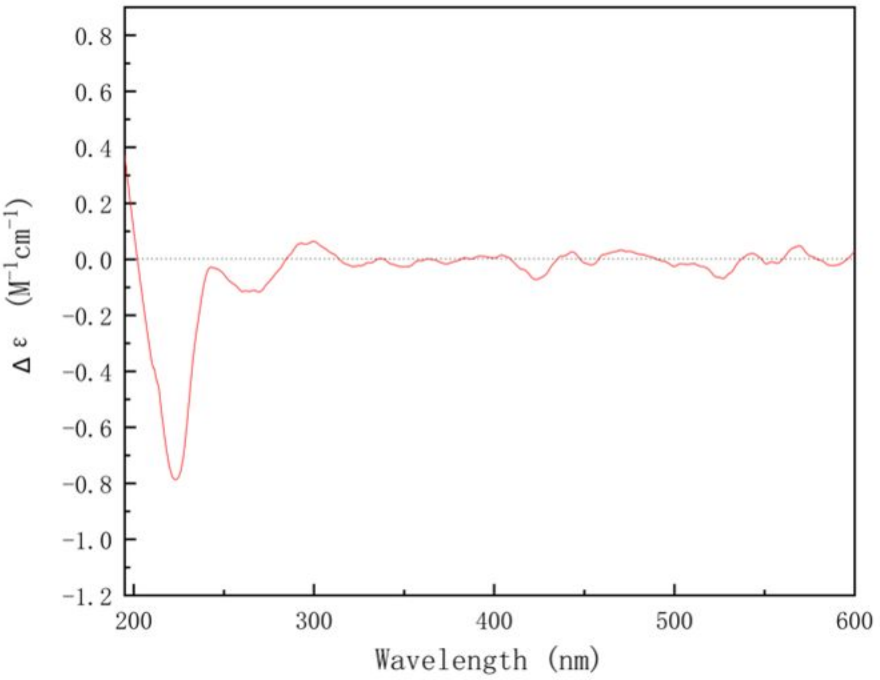

Figure S 76 CD spectrum of compound **7** in MeOH.

Table S 1 Gibbs free energies[a] and equilibrium populations[b] of low-energy conformers of compound **1**.

Conformers	ΔG (a.u.)	P (%)/100	Single point energy (a.u.)
**1**-1	0.00142	18.15	-2711.664995
**1**-2	0.00000	81.85	-2711.666417
**1**-3	0.00944	0.00	-2711.662238
**1**-4	0.00578	0.18	-2711.665892
**1**-5	0.00000	83.69	-2711.671675
**1**-6	0.00281	4.26	-2711.668864
**1**-7	0.00199	10.16	-2711.669684
**1**-8	0.00383	1.45	-2711.667847
**1**-9	0.00614	0.13	-2711.665540
**1**-10	0.00619	0.12	-2711.665485

[a]wB97M-V/def2-TZVP, in kcal/mol.
[b]From ΔG values at 298.15K.

Table S 2 Cartesian coordinates for the low-energy reoptimized random search conformers of **1**-1 at B3LYP-D3(BJ)/6-31G* level of theory in methanol.

**1**-1		Standard Orientation (A.U.)			
Center number	Atomic number	Atomic Type	X	Y	Z
1	8	O	4.752877	7.420937	4.421458
2	6	C	6.661552	7.932827	2.849859
3	8	O	8.815898	7.241069	3.194123
4	6	C	5.794137	9.373209	0.497155
5	8	O	3.379613	10.397327	0.685929
6	6	C	6.101515	7.639554	-1.866149
7	6	C	8.743942	7.596645	-3.046657
8	6	C	5.242301	4.876577	-1.571037
9	6	C	2.45598	4.444319	-1.211464
10	8	O	1.773453	2.14395	-2.017236
11	8	O	0.950381	5.911627	-0.28872
12	6	C	-0.810379	1.432842	-1.670834
13	8	O	9.381973	6.05071	-4.603177
14	8	O	10.240026	9.47597	-2.258178
15	6	C	12.827393	9.461182	-3.14254
16	6	C	5.27074	5.673123	6.46013
17	6	C	5.31837	3.017906	5.445023
18	6	C	14.208549	7.128689	-2.271502
19	6	C	7.595777	1.708332	5.115079
20	6	C	7.664145	-0.620402	3.883343
21	6	C	5.422968	-1.638664	2.932674
22	6	C	3.109521	-0.419874	3.338511
23	6	C	3.077335	1.903073	4.580677
24	8	O	5.581873	-3.849955	1.561391
25	6	C	15.479759	5.572149	-3.985997
26	6	C	16.438678	3.224389	-3.257827
27	6	C	16.065391	2.403432	-0.774102
28	6	C	14.939715	4.012512	0.999525
29	6	C	14.022287	6.360045	0.256424
30	8	O	16.605068	0.006054	0.054821
31	6	C	16.665233	-2.10125	-1.684745
32	6	C	3.883668	-4.151161	-0.521016
33	8	O	1.562559	-5.155182	0.270342
34	6	C	1.582374	-7.666929	1.253192
35	6	C	3.016463	-9.540432	-0.443013
36	6	C	5.554841	-8.501938	-1.423684
37	6	C	5.167958	-5.834218	-2.503281
38	8	O	14.81027	-1.981999	-3.523014
39	6	C	12.211031	-2.444875	-2.908083
40	6	C	11.799917	-4.535002	-0.943639
41	6	C	13.813223	-4.584923	1.169794
42	6	C	16.462157	-4.477262	-0.020734
43	6	C	2.376594	-7.793091	4.036765
44	8	O	1.420078	-10.11288	4.955346

45	8	O	1.587718	-10.091713	-2.666247
46	8	O	7.465652	-8.549312	0.469677
47	8	O	3.727849	-5.847868	-4.731321
48	6	C	10.777622	-0.000901	-2.267461
49	8	O	11.77034	-6.916384	-2.205228
50	8	O	13.418437	-2.65541	2.974853
51	8	O	16.981979	-6.642235	-1.454973
52	8	O	8.135041	-0.408365	-2.509904
53	1	H	7.123958	10.951224	0.277152
54	1	H	2.180486	8.993662	0.68273
55	1	H	4.905272	8.555375	-3.310622
56	1	H	5.861368	3.792252	-3.218943
57	1	H	6.20977	3.957257	0.031285
58	1	H	-0.908119	-0.594015	-2.09093
59	1	H	-1.417459	1.80174	0.28023
60	1	H	-2.034405	2.507896	-2.962449
61	1	H	12.845585	9.595788	-5.216503
62	1	H	13.618897	11.204766	-2.338945
63	1	H	7.06999	6.177703	7.36189
64	1	H	3.708814	5.971173	7.7922
65	1	H	9.360479	2.567078	5.750668
66	1	H	9.461805	-1.588884	3.594873
67	1	H	1.367311	-1.282549	2.66571
68	1	H	1.283787	2.895892	4.829719
69	1	H	15.623307	6.14012	-5.966357
70	1	H	17.304798	1.989949	-4.657224
71	1	H	14.687158	3.356539	2.939772
72	1	H	12.98808	7.512505	1.615474
73	1	H	18.474744	-2.089063	-2.711558
74	1	H	3.472185	-2.291553	-1.338561
75	1	H	-0.420594	-8.243002	1.251599
76	1	H	3.364937	-11.269344	0.666947
77	1	H	6.181912	-9.752116	-2.961044
78	1	H	7.009469	-4.996197	-2.944513
79	1	H	11.412818	-3.170117	-4.68775
80	1	H	9.953699	-4.175062	-0.064895
81	1	H	13.625071	-6.418562	2.14228
82	1	H	17.890765	-4.31937	1.487492
83	1	H	1.55002	-6.143231	5.019076
84	1	H	4.443764	-7.684336	4.248881
85	1	H	2.197888	-10.477115	6.562605
86	1	H	-0.069379	-10.680555	-2.174358
87	1	H	7.26027	-7.02994	1.488138
88	1	H	2.397315	-7.098551	-4.513357
89	1	H	11.429463	1.546125	-3.493142
90	1	H	11.102626	0.570692	-0.302225
91	1	H	10.607379	-7.995376	-1.257436
92	1	H	14.485263	-1.239652	2.487114
93	1	H	15.403097	-7.190367	-2.230224

94	1	H	7.706031	-0.39227	-4.284948

Table S 3 Cartesian coordinates for the low-energy reoptimized random search conformers of **1**-2 at B3LYP-D3(BJ)/6-31G* level of theory in methanol.

**1**-2		Standard Orientation (A.U.)			
Center number	Atomic number	Atomic Type	X	Y	Z
1	8	O	4.118263	7.009022	4.183257
2	6	C	6.173008	8.309914	3.418335
3	8	O	8.310313	7.739401	3.969161
4	6	C	5.437385	10.516262	1.696298
5	8	O	3.326935	11.838424	2.567845
6	6	C	5.106758	9.615643	-1.087867
7	6	C	7.530813	9.425238	-2.638499
8	6	C	3.730616	7.083407	-1.500071
9	6	C	1.102447	6.874584	-0.425289
10	8	O	0.22243	4.524594	-0.6865
11	8	O	-0.126496	8.56262	0.52208
12	6	C	-2.234914	4.031239	0.313751
13	8	O	7.515819	8.952867	-4.875888
14	8	O	9.629193	9.748837	-1.274618
15	6	C	12.034397	9.416437	-2.536873
16	6	C	4.53567	4.715707	5.606769
17	6	C	4.742091	2.48714	3.848693
18	6	C	12.937292	6.719455	-2.400584
19	6	C	6.884315	2.201982	2.298507
20	6	C	7.00966	0.263483	0.532528
21	6	C	5.043308	-1.500151	0.350383
22	6	C	2.909733	-1.277738	1.893634
23	6	C	2.776279	0.737893	3.601628
24	8	O	5.435523	-3.415679	-1.348539
25	6	C	11.755284	4.813194	-3.813845
26	6	C	12.547221	2.304176	-3.655435
27	6	C	14.59435	1.671378	-2.103009
28	6	C	15.816843	3.552726	-0.707636
29	6	C	14.976349	6.045716	-0.849246
30	8	O	15.497645	-0.754593	-1.83182
31	6	C	13.91802	-2.803886	-2.536714
32	6	C	3.467877	-5.133321	-2.047385
33	8	O	2.634048	-6.645922	-0.06982
34	6	C	4.307564	-8.531888	0.944161
35	6	C	5.666529	-10.04973	-1.103016
36	6	C	6.67913	-8.415983	-3.295112
37	6	C	4.559625	-6.667237	-4.262842
38	8	O	11.706671	-2.852643	-1.082982
39	6	C	11.949941	-3.33741	1.596219
40	6	C	13.46117	-5.748984	2.109659
41	6	C	15.913495	-5.861085	0.522973
42	6	C	15.420853	-5.264317	-2.288157
43	6	C	6.064003	-7.400983	2.966014
44	8	O	7.538108	-9.284345	4.093806

45	8	O	3.863882	-11.809088	-2.090293
46	8	O	8.895641	-7.059106	-2.585202
47	8	O	2.648516	-8.040272	-5.470013
48	6	C	12.814368	-1.024726	3.138295
49	8	O	11.969271	-7.95886	1.745768
50	8	O	17.781169	-4.227563	1.521431
51	8	O	14.169037	-7.218545	-3.582648
52	8	O	11.837589	-1.178306	5.610848
53	1	H	7.054556	11.813524	1.75451
54	1	H	1.820756	10.863162	2.137231
55	1	H	3.981463	11.119281	-1.989847
56	1	H	3.58558	6.74824	-3.549313
57	1	H	4.836321	5.486484	-0.759228
58	1	H	-3.673898	5.153772	-0.681115
59	1	H	-2.569617	2.006488	0.032867
60	1	H	-2.304053	4.506014	2.335901
61	1	H	11.826859	10.042693	-4.505197
62	1	H	13.335312	10.679406	-1.532871
63	1	H	6.256012	4.955173	6.7432
64	1	H	2.889449	4.540515	6.85537
65	1	H	8.439602	3.54832	2.450067
66	1	H	8.640406	0.046263	-0.700869
67	1	H	1.406546	-2.680412	1.810053
68	1	H	1.09389	0.930523	4.78494
69	1	H	10.17897	5.301346	-5.049138
70	1	H	11.551712	0.870396	-4.744801
71	1	H	17.398283	3.032074	0.509132
72	1	H	15.922014	7.491919	0.281612
73	1	H	13.28829	-2.594594	-4.507294
74	1	H	1.800627	-4.056502	-2.673271
75	1	H	3.052259	-9.8858	1.895937
76	1	H	7.235899	-11.049213	-0.168218
77	1	H	7.215952	-9.68045	-4.860819
78	1	H	5.352817	-5.32094	-5.638365
79	1	H	9.994482	-3.674457	2.198936
80	1	H	13.998432	-5.737861	4.116066
81	1	H	16.627019	-7.810976	0.642044
82	1	H	17.271479	-4.991049	-3.203086
83	1	H	4.845081	-6.4689	4.381781
84	1	H	7.237992	-5.914822	2.120438
85	1	H	9.29375	-8.982611	3.59792
86	1	H	4.748203	-13.066402	-3.078215
87	1	H	8.461903	-5.327535	-2.11285
88	1	H	2.26392	-9.487238	-4.39721
89	1	H	12.206079	0.707913	2.159852
90	1	H	14.87851	-0.968527	3.315647
91	1	H	11.220102	-7.935857	0.064525
92	1	H	17.630709	-2.606108	0.685778
93	1	H	12.3405	-7.049052	-3.446536

94	1	H	10.062697	-0.744381	5.541665

Table S 4 Cartesian coordinates for the low-energy reoptimized random search conformers of **1**-3 at B3LYP-D3(BJ)/6-31G* level of theory in methanol.

**1**-3		Standard Orientation (A.U.)			
Center number	Atomic number	Atomic Type	X	Y	Z
1	8	O	2.836765	-1.360667	-10.24831
2	6	C	3.749792	-3.682634	-10.583735
3	8	O	2.528279	-5.535602	-11.142782
4	6	C	6.550824	-3.827401	-9.979711
5	8	O	7.671867	-5.852223	-11.250516
6	6	C	6.732173	-4.060831	-7.026053
7	6	C	8.724321	-2.390116	-5.823724
8	6	C	6.980009	-6.818748	-6.171376
9	6	C	6.439741	-7.121475	-3.378116
10	8	O	8.232564	-8.390684	-2.161972
11	8	O	4.539824	-6.314466	-2.379125
12	6	C	8.004993	-8.530756	0.542421
13	8	O	9.782413	-2.887193	-3.851653
14	8	O	9.075525	-0.234006	-7.083946
15	6	C	10.457688	1.758998	-5.828284
16	6	C	0.136439	-1.09226	-9.759743
17	6	C	-0.257817	-1.426579	-6.971553
18	6	C	8.801701	3.081021	-3.926427
19	6	C	-0.669306	-3.845413	-5.958038
20	6	C	-0.578191	-4.239407	-3.365443
21	6	C	-0.04474	-2.217849	-1.744028
22	6	C	0.268143	0.224826	-2.699919
23	6	C	0.160051	0.590141	-5.3115
24	8	O	0.233045	-2.837774	0.749159
25	6	C	9.670669	3.629224	-1.494992
26	6	C	8.109862	4.781451	0.296929
27	6	C	5.609839	5.361884	-0.343008
28	6	C	4.739042	4.888364	-2.800462
29	6	C	6.315005	3.752381	-4.566758
30	8	O	3.839929	6.290411	1.296977
31	6	C	4.529636	7.375603	3.604022
32	6	C	0.308879	-0.98322	2.673153
33	8	O	2.44443	0.577344	2.594924
34	6	C	4.912524	-0.456837	3.208316
35	6	C	4.873304	-2.223929	5.50996
36	6	C	2.573433	-4.021729	5.550984
37	6	C	0.14039	-2.450336	5.16166
38	8	O	5.704173	5.49534	5.113931
39	6	C	5.650738	6.11488	7.72165
40	6	C	2.940459	5.844248	8.754024
41	6	C	0.995124	6.381055	6.649183
42	6	C	2.085013	8.398778	4.866392
43	6	C	6.062569	-1.560805	0.814453
44	8	O	8.631996	-2.108658	1.29501

45	8	O	4.938446	-0.841166	7.814121
46	8	O	2.787334	-6.07087	3.871874
47	8	O	-0.125825	-0.755637	7.22659
48	6	C	7.529341	4.484732	9.145466
49	8	O	2.549111	7.626393	10.72012
50	8	O	0.227835	4.178461	5.356844
51	8	O	2.588425	10.648217	6.20188
52	8	O	7.247769	5.167683	11.741598
53	1	H	4.964727	-3.305421	-6.201804
54	1	H	7.461139	-2.058382	-10.562058
55	1	H	6.321325	-7.035746	-11.635558
56	1	H	5.540722	-7.93204	-7.181076
57	1	H	8.841597	-7.586843	-6.650494
58	1	H	8.457478	-6.671144	1.353531
59	1	H	6.082994	-9.060265	1.115534
60	1	H	9.38356	-9.95042	1.155421
61	1	H	12.153682	0.970344	-4.928947
62	1	H	11.025916	3.019303	-7.378589
63	1	H	-0.325341	0.822628	-10.404678
64	1	H	-0.892724	-2.492718	-10.891196
65	1	H	-0.982706	-5.442077	-7.227025
66	1	H	-0.809007	-6.119695	-2.558883
67	1	H	0.715486	1.787801	-1.442321
68	1	H	0.48728	2.481268	-6.074684
69	1	H	11.581967	3.080578	-0.940559
70	1	H	8.791765	5.094945	2.208998
71	1	H	2.799139	5.404105	-3.267024
72	1	H	5.594118	3.317708	-6.451494
73	1	H	5.864995	8.960245	3.301381
74	1	H	-1.304767	0.318548	2.445961
75	1	H	6.02127	1.207389	3.746314
76	1	H	6.607524	-3.364458	5.413757
77	1	H	2.489715	-4.823435	7.470493
78	1	H	-1.499763	-3.732377	5.047262
79	1	H	6.197319	8.115923	7.965457
80	1	H	2.626372	3.897049	9.430956
81	1	H	-0.699027	7.15793	7.571615
82	1	H	0.740062	8.791505	3.326652
83	1	H	5.856351	-0.12734	-0.683951
84	1	H	5.032982	-3.253961	0.205179
85	1	H	9.414974	-2.437036	-0.332068
86	1	H	3.176352	-0.489302	8.230455
87	1	H	2.320151	-5.528244	2.184553
88	1	H	-0.430993	0.980271	6.647512
89	1	H	9.466191	4.875706	8.465613
90	1	H	7.081905	2.478777	8.815206
91	1	H	3.915209	7.336722	11.917795
92	1	H	1.571301	3.513306	4.270165
93	1	H	2.535972	10.298304	8.006773

94	1	H	7.745647	3.753798	12.777567

Table S 5 Cartesian coordinates for the low-energy reoptimized random search conformers of **1**-4 at B3LYP-D3(BJ)/6-31G* level of theory in methanol.

**1**-4		Standard Orientation (A.U.)			
Center number	Atomic number	Atomic Type	X	Y	Z
1	8	O	5.478033	-2.316162	-10.828012
2	6	C	5.710378	-4.807899	-10.596636
3	8	O	4.239891	-6.363204	-11.407237
4	6	C	8.014714	-5.58625	-9.048764
5	8	O	8.672316	-8.071921	-9.654459
6	6	C	7.354695	-5.337304	-6.193417
7	6	C	6.620863	-2.663656	-5.469172
8	6	C	9.550489	-6.316557	-4.559036
9	6	C	8.883504	-6.253728	-1.778487
10	8	O	6.980332	-7.831253	-1.249349
11	8	O	9.883811	-4.90616	-0.225915
12	6	C	6.033702	-7.794133	1.291625
13	8	O	4.604635	-2.066624	-4.598205
14	8	O	8.512123	-1.02188	-5.908093
15	6	C	7.953848	1.6005	-5.521921
16	6	C	3.086618	-1.327921	-11.795028
17	6	C	1.340758	-0.843654	-9.616287
18	6	C	7.837967	2.374998	-2.779999
19	6	C	1.344041	1.497496	-8.388405
20	6	C	0.031872	1.87546	-6.134086
21	6	C	-1.337107	-0.135618	-5.096202
22	6	C	-1.441522	-2.46317	-6.352629
23	6	C	-0.104338	-2.814453	-8.584729
24	8	O	-2.610143	-0.002574	-2.851865
25	6	C	9.124366	1.037722	-0.901222
26	6	C	9.03573	1.808776	1.622692
27	6	C	7.71891	4.009567	2.255606
28	6	C	6.404091	5.359861	0.403299
29	6	C	6.442471	4.518643	-2.090607
30	8	O	7.564271	4.95097	4.695675
31	6	C	9.345828	4.061203	6.528705
32	6	C	-2.639129	2.310759	-1.430557
33	8	O	-0.259678	3.003891	-0.553714
34	6	C	0.84284	1.657092	1.537269
35	6	C	-1.056436	1.251477	3.695577
36	6	C	-3.540924	0.144511	2.699695
37	6	C	-4.619834	1.912825	0.659721
38	8	O	8.697072	1.671828	7.440682
39	6	C	6.652535	1.455135	9.202507
40	6	C	6.95414	3.29859	11.424452
41	6	C	7.54008	6.043423	10.611862
42	6	C	9.697477	6.023302	8.640672
43	6	C	2.231508	-0.770903	0.656265
44	8	O	2.31908	-2.668326	2.548022

45	8	O	-1.615572	3.552795	4.952513
46	8	O	-3.093556	-2.347377	1.782709
47	8	O	-5.387465	4.235086	1.663401
48	6	C	4.06895	1.44106	7.911084
49	8	O	9.003673	2.318344	12.881709
50	8	O	5.402253	7.420912	9.893234
51	8	O	12.026989	5.504077	9.831558
52	8	O	3.259264	3.836218	6.961581
53	1	H	5.678593	-6.507767	-5.825701
54	1	H	9.606611	-4.318067	-9.481337
55	1	H	7.13043	-8.869244	-10.261025
56	1	H	9.946737	-8.267934	-5.151468
57	1	H	11.247383	-5.158417	-4.842495
58	1	H	5.042017	-6.006398	1.673679
59	1	H	4.716075	-9.388828	1.424201
60	1	H	7.590098	-8.024625	2.648134
61	1	H	9.496721	2.592817	-6.501664
62	1	H	6.160961	2.053536	-6.467147
63	1	H	3.609707	0.421873	-12.775392
64	1	H	2.317213	-2.697645	-13.147886
65	1	H	2.44944	3.055761	-9.176831
66	1	H	0.170302	3.674778	-5.148565
67	1	H	-2.549296	-3.992378	-5.522543
68	1	H	-0.124038	-4.655822	-9.515257
69	1	H	10.14289	-0.693661	-1.355394
70	1	H	9.934002	0.650833	3.067078
71	1	H	5.348898	7.047406	0.944202
72	1	H	5.365172	5.551797	-3.517739
73	1	H	11.178304	3.819864	5.585411
74	1	H	-3.259511	3.861739	-2.669451
75	1	H	2.260135	2.987205	2.253239
76	1	H	-0.229108	-0.109602	5.030187
77	1	H	-4.905281	0.045683	4.26795
78	1	H	-6.273923	1.01266	-0.230918
79	1	H	6.891213	-0.448503	10.006287
80	1	H	5.183142	3.296801	12.53402
81	1	H	8.343836	7.015341	12.274879
82	1	H	9.817802	7.906242	7.770477
83	1	H	4.190728	-0.296513	0.183045
84	1	H	1.383476	-1.493263	-1.091419
85	1	H	0.599633	-3.315985	2.675014
86	1	H	-0.056457	4.094078	5.777405
87	1	H	-3.003368	-2.262758	-0.050891
88	1	H	-4.105003	4.740746	2.889878
89	1	H	2.635643	0.845156	9.294727
90	1	H	4.10456	0.030321	6.375199
91	1	H	9.174921	3.340189	14.386985
92	1	H	4.264022	6.328826	8.940392
93	1	H	11.781488	3.959883	10.797805

94	1	H	4.585237	4.443165	5.801143

Table S 6 Cartesian coordinates for the low-energy reoptimized random search conformers of **1**-5 at B3LYP-D3(BJ)/6-31G* level of theory in methanol.

**1**-5		Standard Orientation (A.U.)			
Center number	Atomic number	Atomic Type	X	Y	Z
1	8	O	5.837905	-0.89936	-9.416558
2	6	C	6.042325	-3.409385	-9.622087
3	8	O	4.497739	-4.772796	-10.608072
4	6	C	8.403647	-4.351032	-8.266161
5	8	O	9.270317	-6.632701	-9.240537
6	6	C	7.749445	-4.46678	-5.357196
7	6	C	9.776856	-3.31346	-3.683668
8	6	C	7.086529	-7.145712	-4.447335
9	6	C	4.607864	-8.049061	-5.535114
10	8	O	4.945989	-9.51753	-7.593707
11	8	O	2.550693	-7.505534	-4.716006
12	6	C	2.709759	-10.290254	-8.909447
13	8	O	10.37507	-4.114317	-1.616655
14	8	O	10.728669	-1.198585	-4.657757
15	6	C	12.221528	0.377419	-2.974689
16	6	C	3.341866	0.200453	-9.759245
17	6	C	1.925925	-0.075316	-7.315709
18	6	C	10.582019	1.524734	-0.958091
19	6	C	2.409988	1.578	-5.305326
20	6	C	1.450045	1.127206	-2.892278
21	6	C	-0.015487	-1.037812	-2.477731
22	6	C	-0.58792	-2.663176	-4.480845
23	6	C	0.382436	-2.184829	-6.875465
24	8	O	-0.899747	-1.767533	-0.153779
25	6	C	8.663045	3.22617	-1.612472
26	6	C	6.995481	4.182546	0.191162
27	6	C	7.225005	3.404101	2.71577
28	6	C	9.193237	1.76952	3.407823
29	6	C	10.834017	0.824919	1.584269
30	8	O	5.61013	4.084064	4.595021
31	6	C	3.409317	5.50689	3.970375
32	6	C	-0.392835	-0.296967	2.041495
33	8	O	2.177682	0.041921	2.49594
34	6	C	3.697004	-2.083358	3.21513
35	6	C	2.452693	-3.683713	5.27307
36	6	C	-0.376914	-4.18648	4.788498
37	6	C	-1.71825	-1.660793	4.234965
38	8	O	4.030874	7.969146	3.274077
39	6	C	5.125001	9.582122	5.153071
40	6	C	4.426075	8.73185	7.831073
41	6	C	1.73465	7.749584	7.812953
42	6	C	1.593528	5.324985	6.255964
43	6	C	4.640696	-3.581117	0.919877
44	8	O	6.492948	-5.31636	1.733471

45	8	O	2.548141	-2.393006	7.663049
46	8	O	-0.757357	-5.993776	2.885645
47	8	O	-1.717909	-0.076454	6.396423
48	6	C	7.978188	9.872906	4.802742
49	8	O	4.716341	10.827999	9.464431
50	8	O	0.982218	7.383837	10.351509
51	8	O	-0.930626	4.940386	5.514087
52	8	O	8.920144	11.875662	6.275327
53	1	H	6.11136	-3.213822	-5.01954
54	1	H	9.887075	-2.928176	-8.534571
55	1	H	7.835855	-7.776617	-9.374005
56	1	H	8.618563	-8.441245	-4.967551
57	1	H	6.876779	-7.128099	-2.386112
58	1	H	3.331111	-11.651655	-10.342202
59	1	H	1.364231	-11.169101	-7.595462
60	1	H	1.838126	-8.640152	-9.817577
61	1	H	13.749565	-0.762748	-2.154962
62	1	H	13.026405	1.808214	-4.241871
63	1	H	3.708173	2.183216	-10.240737
64	1	H	2.390523	-0.748913	-11.338868
65	1	H	3.619284	3.225334	-5.605946
66	1	H	1.938944	2.388119	-1.348747
67	1	H	-1.704307	-4.354539	-4.113877
68	1	H	0.010215	-3.516311	-8.404496
69	1	H	8.449942	3.808222	-3.58303
70	1	H	5.564696	5.556277	-0.350465
71	1	H	9.373377	1.22582	5.389265
72	1	H	12.303635	-0.516505	2.130084
73	1	H	2.483174	4.667047	2.319028
74	1	H	-1.15981	1.622658	1.797926
75	1	H	5.399897	-1.224562	4.041024
76	1	H	3.46111	-5.500721	5.380132
77	1	H	-1.169075	-4.951132	6.556994
78	1	H	-3.688972	-2.033322	3.684398
79	1	H	4.258147	11.456241	4.877329
80	1	H	5.691951	7.174039	8.429448
81	1	H	0.515714	9.167552	6.876915
82	1	H	2.280331	3.752745	7.439139
83	1	H	5.399729	-2.189285	-0.433149
84	1	H	3.09552	-4.599165	-0.028929
85	1	H	8.070531	-4.955367	0.859664
86	1	H	4.236739	-1.749407	7.931252
87	1	H	-0.832476	-5.127457	1.272299
88	1	H	-0.202763	-0.515741	7.372694
89	1	H	8.370093	10.292406	2.800801
90	1	H	8.916305	8.050728	5.244729
91	1	H	3.824063	10.404227	11.008511
92	1	H	-0.785715	6.923077	10.339329
93	1	H	-1.385238	3.171602	5.834143

94	1	H	8.047509	11.855217	7.889132

Table S 7 Cartesian coordinates for the low-energy reoptimized random search conformers of **1**-6 at B3LYP-D3(BJ)/6-31G* level of theory in methanol.

**1**-6		Standard Orientation (A.U.)			
Center number	Atomic number	Atomic Type	X	Y	Z
1	8	O	5.115293	-2.03588	-10.842731
2	6	C	6.359812	-4.207037	-10.63774
3	8	O	5.63571	-6.237713	-11.406506
4	6	C	8.783612	-3.9693	-9.093766
5	8	O	10.192921	-6.186426	-9.336597
6	6	C	7.955104	-3.50812	-6.312687
7	6	C	6.870755	-0.891411	-5.871323
8	6	C	10.1325	-3.961517	-4.439539
9	6	C	9.09153	-4.135782	-1.789432
10	8	O	10.268148	-2.650724	-0.148088
11	8	O	7.312639	-5.489765	-1.23931
12	6	C	9.384701	-2.669181	2.41772
13	8	O	4.881573	-0.483059	-4.832501
14	8	O	8.476573	0.918353	-6.621355
15	6	C	7.847899	3.496011	-5.933984
16	6	C	2.458186	-2.138076	-11.594459
17	6	C	0.854781	-2.348117	-9.261503
18	6	C	7.993465	3.876868	-3.120308
19	6	C	-0.08619	-0.178178	-8.073783
20	6	C	-1.250243	-0.316073	-5.717874
21	6	C	-1.491259	-2.655467	-4.519512
22	6	C	-0.679303	-4.861806	-5.735457
23	6	C	0.500186	-4.697713	-8.085634
24	8	O	-2.495782	-2.721176	-2.119697
25	6	C	5.806883	4.344142	-1.715365
26	6	C	5.873502	4.453391	0.916597
27	6	C	8.151486	3.982259	2.170696
28	6	C	10.380876	3.608446	0.794757
29	6	C	10.292583	3.565876	-1.8349
30	8	O	8.321317	3.793301	4.763386
31	6	C	6.05888	3.075229	6.095567
32	6	C	-0.994353	-3.982626	-0.192512
33	8	O	-2.29368	-3.906241	2.067008
34	6	C	-2.211634	-1.719183	3.689236
35	6	C	0.436045	-0.510022	3.854907
36	6	C	1.698431	-0.249397	1.245345
37	6	C	1.6687	-2.85519	-0.054544
38	8	O	4.469269	5.113938	6.527925
39	6	C	5.066153	6.854603	8.54508
40	6	C	5.701073	5.465392	11.03079
41	6	C	7.636618	3.302212	10.681218
42	6	C	6.748412	1.597129	8.495162
43	6	C	-4.397909	0.064959	3.068118
44	8	O	-4.264011	1.185705	0.64144

45	8	O	2.016444	-1.952954	5.490579
46	8	O	0.687659	1.717405	-0.205324
47	8	O	3.118075	-4.575915	1.431765
48	6	C	6.924387	8.888281	7.678968
49	8	O	3.462637	4.541501	12.140196
50	8	O	10.146667	4.087422	10.422581
51	8	O	4.550524	0.34196	9.360284
52	8	O	9.453314	8.081616	7.337916
53	1	H	6.44479	-4.857337	-5.84871
54	1	H	9.898119	-2.336826	-9.74886
55	1	H	9.039775	-7.44608	-10.023699
56	1	H	11.008388	-5.786207	-4.920598
57	1	H	11.572654	-2.475053	-4.560415
58	1	H	9.771949	-4.514141	3.292658
59	1	H	10.41551	-1.164931	3.392513
60	1	H	7.344453	-2.302537	2.49232
61	1	H	9.25175	4.628952	-6.959016
62	1	H	5.947827	3.938186	-6.644656
63	1	H	2.131689	-0.357463	-12.602734
64	1	H	2.209542	-3.741818	-12.884691
65	1	H	0.177676	1.668008	-8.961514
66	1	H	-1.859808	1.38772	-4.736659
67	1	H	-0.93131	-6.696874	-4.830958
68	1	H	1.196642	-6.410379	-9.002597
69	1	H	3.982434	4.518066	-2.655906
70	1	H	4.144077	4.829492	1.962877
71	1	H	12.145775	3.286511	1.812389
72	1	H	12.025881	3.203998	-2.898283
73	1	H	5.00724	1.804407	4.841677
74	1	H	-0.847242	-5.997367	-0.682908
75	1	H	-2.592962	-2.484945	5.585758
76	1	H	0.249772	1.377613	4.714178
77	1	H	3.703729	0.204264	1.549788
78	1	H	2.465199	-2.657291	-1.960055
79	1	H	3.260939	7.822864	8.9014
80	1	H	6.526561	6.858669	12.343997
81	1	H	7.510327	2.132805	12.399696
82	1	H	8.285705	0.266038	8.01636
83	1	H	-4.42818	1.610272	4.462024
84	1	H	-6.174353	-1.011289	3.272788
85	1	H	-4.246663	-0.18283	-0.603422
86	1	H	2.642849	-3.384912	4.486357
87	1	H	-1.15119	1.813408	0.050101
88	1	H	4.661072	-5.058803	0.525383
89	1	H	6.963144	10.384042	9.12603
90	1	H	6.179973	9.73364	5.918967
91	1	H	3.122242	2.923211	11.3264
92	1	H	10.196298	5.688045	9.497764
93	1	H	3.714674	-0.584917	7.983737

94	1	H	9.445484	6.762736	6.048587

Table S 8 Cartesian coordinates for the low-energy reoptimized random search conformers of **1**-7 at B3LYP-D3(BJ)/6-31G* level of theory in methanol.

**1**-7		Standard Orientation (A.U.)			
Center number	Atomic number	Atomic Type	X	Y	Z
1	8	O	5.868089	-1.864347	-10.640498
2	6	C	6.18851	-4.354784	-10.66704
3	8	O	4.871068	-5.863181	-11.773782
4	6	C	8.363632	-5.22727	-8.97893
5	8	O	9.062328	-7.680751	-9.64565
6	6	C	7.444983	-5.110329	-6.18801
7	6	C	6.760748	-2.458221	-5.368288
8	6	C	9.403886	-6.269201	-4.371138
9	6	C	8.342332	-6.271225	-1.714166
10	8	O	6.642545	-8.114961	-1.411156
11	8	O	8.860055	-4.727703	-0.106818
12	6	C	5.245465	-8.144107	0.915982
13	8	O	4.738885	-1.87358	-4.477958
14	8	O	8.654832	-0.824782	-5.702075
15	6	C	8.100672	1.801556	-5.273355
16	6	C	3.495004	-0.835862	-11.641522
17	6	C	1.663197	-0.536468	-9.504247
18	6	C	7.898245	2.536343	-2.527265
19	6	C	1.5469	1.714818	-8.120043
20	6	C	0.163199	1.895011	-5.882449
21	6	C	-1.159336	-0.229287	-5.007084
22	6	C	-1.150147	-2.466262	-6.440174
23	6	C	0.254289	-2.61877	-8.650494
24	8	O	-2.48331	-0.331092	-2.827971
25	6	C	9.278952	1.305325	-0.640866
26	6	C	9.1429	2.096113	1.875872
27	6	C	7.657012	4.192116	2.495684
28	6	C	6.21026	5.400665	0.64456
29	6	C	6.321648	4.553581	-1.843961
30	8	O	7.477769	5.167906	4.912919
31	6	C	9.447796	4.681683	6.633967
32	6	C	-2.53867	1.745399	-1.118309
33	8	O	-0.149595	2.387647	-0.190404
34	6	C	1.012721	0.960019	1.80285
35	6	C	-0.857472	0.066537	3.850809
36	6	C	-3.351778	-0.946911	2.757482
37	6	C	-4.460312	1.042333	0.934095
38	8	O	9.255747	2.311895	7.798554
39	6	C	7.136903	1.907755	9.478069
40	6	C	6.856622	4.033113	11.465225
41	6	C	7.244946	6.729411	10.385949
42	6	C	9.568066	6.753984	8.653387
43	6	C	2.826383	-1.041181	0.802191

44	8	O	1.650578	-3.080164	-0.48046
45	8	O	-1.49423	2.093037	5.492769
46	8	O	-3.090309	-3.355087	1.697569
47	8	O	-5.246407	3.2337	2.214553
48	6	C	4.708133	1.22837	8.059799
49	8	O	8.540054	3.64612	13.499201
50	8	O	5.198625	7.712712	9.002079
51	8	O	11.769316	6.303773	10.140558
52	8	O	3.205861	3.305287	7.29583
53	1	H	5.696768	-6.225772	-6.04923
54	1	H	9.985969	-3.94217	-9.202992
55	1	H	7.613496	-8.398396	-10.524046
56	1	H	9.795448	-8.202453	-5.01461
57	1	H	11.155872	-5.15772	-4.387197
58	1	H	3.839844	-6.612227	0.892684
59	1	H	4.300578	-9.986663	0.989347
60	1	H	6.524545	-7.912537	2.53516
61	1	H	9.684448	2.791294	-6.186512
62	1	H	6.346291	2.278334	-6.2743
63	1	H	4.032648	0.983758	-12.47646
64	1	H	2.811162	-2.121515	-13.116732
65	1	H	2.610753	3.362122	-8.775862
66	1	H	0.200271	3.630546	-4.778763
67	1	H	-2.233472	-4.073049	-5.734405
68	1	H	0.312013	-4.389216	-9.711031
69	1	H	10.398856	-0.362708	-1.094553
70	1	H	10.1443	1.046619	3.337178
71	1	H	5.007732	6.986356	1.183088
72	1	H	5.155136	5.477759	-3.274741
73	1	H	11.255427	4.638999	5.599193
74	1	H	-3.203734	3.446514	-2.118628
75	1	H	2.251592	2.342968	2.737439
76	1	H	0.050136	-1.487807	4.914333
77	1	H	-4.683188	-1.104419	4.349681
78	1	H	-6.102245	0.205398	-0.03074
79	1	H	7.699907	0.214201	10.539785
80	1	H	4.924013	3.901558	12.243023
81	1	H	7.678145	7.94273	12.032853
82	1	H	9.722724	8.611848	7.731325
83	1	H	3.918516	-1.787659	2.418712
84	1	H	4.168833	-0.108935	-0.466205
85	1	H	2.249995	-3.029238	-2.21936
86	1	H	0.075502	2.750302	6.21697
87	1	H	-1.672254	-3.353081	0.516028
88	1	H	-4.02118	3.52609	3.559827
89	1	H	3.51588	0.093453	9.33181
90	1	H	5.225674	0.01913	6.436105
91	1	H	10.075713	4.571736	13.114864
92	1	H	3.638549	7.253957	9.833203

93	1	H	12.333141	4.595104	9.795733
94	1	H	4.287572	4.540681	6.450656

Table S 9 Cartesian coordinates for the low-energy reoptimized random search conformers of **1**-8 at B3LYP-D3(BJ)/6-31G* level of theory in methanol.

**1**-8		Standard Orientation (A.U.)			
Center number	Atomic number	Atomic Type	X	Y	Z
1	8	O	4.372143	-2.986088	-9.459976
2	6	C	5.742752	-5.053694	-9.0322
3	8	O	4.935642	-7.194358	-9.013289
4	6	C	8.469336	-4.373664	-8.408308
5	8	O	10.103365	-6.381717	-8.885882
6	6	C	8.537121	-3.43564	-5.588474
7	6	C	10.181625	-1.113699	-5.22719
8	6	C	9.283226	-5.511758	-3.709542
9	6	C	7.263011	-7.513913	-3.576712
10	8	O	7.906242	-9.659116	-4.73542
11	8	O	5.211863	-7.207618	-2.583622
12	6	C	5.998304	-11.580701	-4.899597
13	8	O	11.788071	-0.859915	-3.640328
14	8	O	9.471095	0.717535	-6.856203
15	6	C	10.160531	3.252712	-6.210921
16	6	C	1.637834	-3.235795	-9.489913
17	6	C	0.628328	-3.047584	-6.844275
18	6	C	8.572939	4.21319	-4.034398
19	6	C	0.340627	-0.682543	-5.685151
20	6	C	-0.404393	-0.470748	-3.168089
21	6	C	-0.871855	-2.667063	-1.77228
22	6	C	-0.628925	-5.040291	-2.90766
23	6	C	0.125333	-5.223741	-5.420075
24	8	O	-1.570225	-2.674835	0.723152
25	6	C	9.348357	6.315151	-2.632137
26	6	C	7.942261	7.197553	-0.57717
27	6	C	5.725962	5.921811	0.100272
28	6	C	4.931868	3.807688	-1.268828
29	6	C	6.330203	2.989254	-3.336374
30	8	O	4.22001	6.568259	2.115855
31	6	C	4.741527	8.724651	3.600773
32	6	C	-1.422846	-0.43709	2.193955
33	8	O	1.065667	0.438445	2.436526
34	6	C	2.867924	-1.10978	3.799156
35	6	C	1.8438	-1.869585	6.40992
36	6	C	-0.919401	-2.821445	6.285563
37	6	C	-2.646455	-1.033787	4.754473
38	8	O	7.082384	8.624739	4.832047
39	6	C	7.308496	7.081929	7.064288
40	6	C	5.215613	5.090874	7.199345
41	6	C	2.566584	6.304136	7.00372
42	6	C	2.611511	8.839884	5.544323
43	6	C	3.863625	-3.36672	2.293236
44	8	O	4.676108	-2.602952	-0.108496

45	8	O	2.081005	0.120515	8.196598
46	8	O	-1.022386	-5.308181	5.307326
47	8	O	-3.330993	1.183741	6.038851
48	6	C	9.947801	5.904785	7.058485
49	8	O	5.560023	3.683664	9.434576
50	8	O	0.826822	4.570672	5.864836
51	8	O	2.942045	10.937961	7.139372
52	8	O	10.605091	4.946372	9.43441
53	1	H	6.63045	-2.753719	-5.09726
54	1	H	9.011309	-2.781992	-9.624202
55	1	H	9.274147	-7.925912	-8.341145
56	1	H	11.107737	-6.335313	-4.238546
57	1	H	9.435719	-4.634741	-1.834975
58	1	H	6.940506	-13.246753	-5.690789
59	1	H	5.224507	-11.996272	-3.018946
60	1	H	4.485458	-10.930668	-6.162795
61	1	H	12.18134	3.340164	-5.729987
62	1	H	9.834857	4.34786	-7.948091
63	1	H	0.994255	-1.661497	-10.675579
64	1	H	1.138597	-5.039545	-10.383585
65	1	H	0.739978	1.03675	-6.758174
66	1	H	-0.555548	1.390535	-2.307735
67	1	H	-0.984679	-6.730734	-1.782478
68	1	H	0.377619	-7.081749	-6.277655
69	1	H	11.115688	7.273529	-3.110651
70	1	H	8.632191	8.785584	0.532908
71	1	H	3.264929	2.769444	-0.652135
72	1	H	5.666215	1.344934	-4.380716
73	1	H	4.813277	10.42273	2.396341
74	1	H	-2.468439	1.107631	1.270566
75	1	H	4.467217	0.189338	4.069599
76	1	H	2.990277	-3.442516	7.137969
77	1	H	-1.62907	-2.862748	8.240102
78	1	H	-4.410672	-2.071103	4.376418
79	1	H	7.172437	8.299666	8.757719
80	1	H	5.453353	3.867257	5.526379
81	1	H	1.861444	6.693558	8.916843
82	1	H	0.779619	9.046116	4.582318
83	1	H	2.429564	-4.867482	2.183985
84	1	H	5.436687	-4.150154	3.438792
85	1	H	4.70092	-4.12279	-1.155659
86	1	H	1.224487	1.616091	7.532713
87	1	H	-1.312022	-5.195858	3.50318
88	1	H	-2.019355	2.468125	5.963537
89	1	H	11.313633	7.391641	6.54915
90	1	H	10.019432	4.459943	5.535827
91	1	H	4.600041	2.104492	9.259881
92	1	H	1.433057	4.174152	4.168721
93	1	H	4.7345	11.229879	7.345314

94	1	H	9.163595	3.956158	10.001763

Table S 10 Cartesian coordinates for the low-energy reoptimized random search conformers of **1**-9 at B3LYP-D3(BJ)/6-31G* level of theory in methanol.

**1**-9		Standard Orientation (A.U.)			
Center number	Atomic number	Atomic Type	X	Y	Z
1	8	O	5.715485	-2.199805	-10.79627
2	6	C	6.098673	-4.673669	-10.574311
3	8	O	4.726493	-6.313422	-11.39042
4	6	C	8.44749	-5.312405	-9.029769
5	8	O	9.25815	-7.751344	-9.640877
6	6	C	7.775048	-5.111942	-6.173663
7	6	C	6.876479	-2.491791	-5.43885
8	6	C	10.028153	-5.959347	-4.544066
9	6	C	9.368337	-5.935676	-1.761039
10	8	O	7.524435	-7.580824	-1.235039
11	8	O	10.32649	-4.560443	-0.205131
12	6	C	6.569026	-7.582262	1.303851
13	8	O	4.826211	-2.024815	-4.568581
14	8	O	8.663504	-0.731465	-5.863566
15	6	C	7.943288	1.846223	-5.4519
16	6	C	3.271888	-1.352922	-11.766572
17	6	C	1.498603	-0.957285	-9.592171
18	6	C	7.81228	2.589664	-2.70106
19	6	C	1.369257	1.385447	-8.373831
20	6	C	0.018991	1.703244	-6.132528
21	6	C	-1.253854	-0.372099	-5.09785
22	6	C	-1.219354	-2.708696	-6.342062
23	6	C	0.154341	-2.998278	-8.561101
24	8	O	-2.5564	-0.295802	-2.868363
25	6	C	9.153469	1.281273	-0.841182
26	6	C	9.033247	2.010048	1.694821
27	6	C	7.635037	4.150522	2.358244
28	6	C	6.269186	5.475105	0.523332
29	6	C	6.334987	4.668124	-1.981259
30	8	O	7.447064	5.060208	4.808841
31	6	C	9.211852	4.168696	6.652182
32	6	C	-2.707778	2.01383	-1.447763
33	8	O	-0.368207	2.833337	-0.572885
34	6	C	0.807363	1.544893	1.515708
35	6	C	-1.064191	1.038658	3.676921
36	6	C	-3.488882	-0.195974	2.684423
37	6	C	-4.661773	1.510807	0.6453
38	8	O	8.63162	1.760078	7.505823
39	6	C	6.556395	1.452187	9.244777
40	6	C	6.80692	3.262478	11.516913
41	6	C	7.28567	6.038777	10.737946
42	6	C	9.444758	6.137368	8.778997
43	6	C	2.319739	-0.805706	0.628383
44	8	O	2.49937	-2.708879	2.506589

45	8	O	-1.73733	3.307251	4.935475
46	8	O	-2.915292	-2.66239	1.767752
47	8	O	-5.550973	3.788758	1.649993
48	6	C	4.00303	1.36302	7.904011
49	8	O	8.74453	2.403418	13.13134
50	8	O	5.136939	7.364992	9.956341
51	8	O	11.692672	5.603313	10.160412
52	8	O	3.102654	3.744539	6.988798
53	1	H	6.17395	-6.383599	-5.80839
54	1	H	9.95697	-3.946549	-9.461206
55	1	H	7.76957	-8.640702	-10.252748
56	1	H	10.536684	-7.884555	-5.137318
57	1	H	11.655665	-4.706659	-4.832945
58	1	H	5.356294	-9.256979	1.447806
59	1	H	8.132104	-7.697743	2.666606
60	1	H	5.460335	-5.859312	1.662149
61	1	H	9.406052	2.944445	-6.441655
62	1	H	6.112521	2.190049	-6.370664
63	1	H	3.694772	0.418796	-12.756123
64	1	H	2.581348	-2.7705	-13.112552
65	1	H	2.396084	2.997136	-9.161282
66	1	H	0.051571	3.512299	-5.155739
67	1	H	-2.250966	-4.29112	-5.513474
68	1	H	0.240582	-4.84236	-9.482802
69	1	H	10.243662	-0.399173	-1.317884
70	1	H	9.965708	0.856432	3.121356
71	1	H	5.150305	7.11353	1.087011
72	1	H	5.213642	5.67646	-3.392269
73	1	H	11.056952	3.98854	5.699866
74	1	H	-3.410841	3.528715	-2.687288
75	1	H	2.152609	2.948465	2.231128
76	1	H	-0.165442	-0.279392	5.00767
77	1	H	-4.843722	-0.364898	4.254866
78	1	H	-6.266569	0.52293	-0.242711
79	1	H	6.85287	-0.454165	10.018489
80	1	H	5.027226	3.199889	12.599071
81	1	H	8.026953	6.992141	12.432912
82	1	H	9.484812	8.038642	7.925145
83	1	H	4.253438	-0.22946	0.164338
84	1	H	1.516345	-1.5598	-1.127059
85	1	H	0.81251	-3.437803	2.633324
86	1	H	-0.213575	3.904798	5.787696
87	1	H	-2.837826	-2.575788	-0.06619
88	1	H	-4.301195	4.360863	2.881018
89	1	H	2.577028	0.687681	9.258079
90	1	H	4.10667	-0.014031	6.340987
91	1	H	10.334439	2.962018	12.400806
92	1	H	4.014029	6.223851	9.04582
93	1	H	13.134346	5.621638	9.039727

94	1	H	4.41223	4.432554	5.865021

Table S 11 Cartesian coordinates for the low-energy reoptimized random search conformers of **1**-10 at B3LYP-D3(BJ)/6-31G* level of theory in methanol.

**1**-10		Standard Orientation (A.U.)			
Center number	Atomic number	Atomic Type	X	Y	Z
1	8	O	5.903943	-1.585724	-10.652457
2	6	C	6.245513	-4.069477	-10.784462
3	8	O	4.960904	-5.536639	-11.981507
4	6	C	8.398008	-4.999029	-9.098087
5	8	O	9.095651	-7.434224	-9.82891
6	6	C	7.453038	-4.96041	-6.313618
7	6	C	6.771628	-2.329175	-5.426904
8	6	C	9.391686	-6.183853	-4.518651
9	6	C	8.328425	-6.25376	-1.863298
10	8	O	6.545356	-8.026845	-1.636573
11	8	O	8.92101	-4.811839	-0.188405
12	6	C	5.154675	-8.099334	0.694378
13	8	O	4.741896	-1.758703	-4.546318
14	8	O	8.675863	-0.693006	-5.690909
15	6	C	8.120131	1.922408	-5.220827
16	6	C	3.538805	-0.531464	-11.648553
17	6	C	1.667325	-0.334776	-9.534234
18	6	C	7.946147	2.62433	-2.462319
19	6	C	1.486451	1.865313	-8.076954
20	6	C	0.049122	1.950516	-5.867193
21	6	C	-1.259536	-0.222295	-5.092862
22	6	C	-1.175765	-2.412174	-6.596085
23	6	C	0.276762	-2.467662	-8.779426
24	8	O	-2.631585	-0.418315	-2.951423
25	6	C	9.13061	1.219788	-0.566639
26	6	C	9.029512	1.988314	1.959879
27	6	C	7.791146	4.236927	2.583762
28	6	C	6.526666	5.620839	0.721052
29	6	C	6.595051	4.7997	-1.777594
30	8	O	7.752063	5.233884	4.994541
31	6	C	9.447315	4.296101	6.822993
32	6	C	-2.83271	1.63094	-1.215643
33	8	O	-0.502343	2.419454	-0.261815
34	6	C	0.754773	1.028844	1.702381
35	6	C	-1.05649	-0.022467	3.727388
36	6	C	-3.488432	-1.164283	2.617238
37	6	C	-4.712423	0.779855	0.819138
38	8	O	8.764218	1.966512	7.855529
39	6	C	6.609558	1.792274	9.537117
40	6	C	6.470443	3.963797	11.49823
41	6	C	7.188009	6.546118	10.347993
42	6	C	9.695583	6.292147	8.903284
43	6	C	2.681533	-0.839053	0.66428
44	8	O	1.628877	-2.960988	-0.593572

45	8	O	-1.810434	1.925448	5.41647
46	8	O	-3.089637	-3.539464	1.527151
47	8	O	-5.628616	2.899637	2.134057
48	6	C	4.127533	1.243108	8.167719
49	8	O	7.953662	3.444893	13.648706
50	8	O	5.229259	7.487861	8.767863
51	8	O	11.647514	5.538547	10.588246
52	8	O	2.938977	3.306299	6.957785
53	1	H	5.698286	-6.070069	-6.222009
54	1	H	10.02599	-3.711899	-9.267779
55	1	H	7.665545	-8.108347	-10.770634
56	1	H	9.767342	-8.102707	-5.214116
57	1	H	11.15618	-5.09253	-4.498327
58	1	H	4.12613	-9.897947	0.686448
59	1	H	6.447971	-8.002557	2.315753
60	1	H	3.819518	-6.506358	0.748139
61	1	H	9.690478	2.932897	-6.135216
62	1	H	6.354283	2.409422	-6.198531
63	1	H	4.076837	1.325259	-12.397053
64	1	H	2.891248	-1.759072	-13.188211
65	1	H	2.540053	3.549463	-8.651126
66	1	H	0.036731	3.645193	-4.701894
67	1	H	-2.245894	-4.059268	-5.967231
68	1	H	0.385321	-4.199865	-9.897658
69	1	H	10.068746	-0.558109	-1.014186
70	1	H	9.869064	0.798822	3.414951
71	1	H	5.504767	7.331867	1.250186
72	1	H	5.579502	5.876929	-3.217678
73	1	H	11.302412	3.972564	5.92903
74	1	H	-3.600697	3.294565	-2.206357
75	1	H	1.912581	2.465283	2.660061
76	1	H	-0.058147	-1.543548	4.758156
77	1	H	-4.808982	-1.417341	4.206429
78	1	H	-6.300384	-0.140515	-0.160177
79	1	H	7.019989	0.085748	10.64877
80	1	H	4.493366	4.090023	12.147441
81	1	H	7.452639	7.875403	11.92854
82	1	H	10.215224	8.116899	8.044162
83	1	H	3.859547	-1.499217	2.258823
84	1	H	3.925164	0.189099	-0.632286
85	1	H	2.258917	-2.92283	-2.321485
86	1	H	-0.270259	2.701138	6.08677
87	1	H	-1.659004	-3.441174	0.364787
88	1	H	-4.421013	3.242669	3.483436
89	1	H	2.842481	0.400892	9.588132
90	1	H	4.487502	-0.206743	6.719215
91	1	H	9.607547	4.183728	13.364895
92	1	H	5.793342	7.387657	7.019407
93	1	H	12.039895	3.781903	10.244068

94	1	H	3.291453	4.88208	7.866652

Table S 12 Effects of gastrodin derivatives in the H_2O_2-induced PC12 cells injury model

Compound	Cell viability (%)		
	5 μM	10 μM	20 μM
Trolox			79.95 ± 3.05
**1**	60.93 ± 8.66	65.25 ± 8.10	78.44 ± 5.50
**2**	58.33 ± 5.42	58.52 ± 6.08	55.46 ± 4.52
**3**	54.70 ± 6.64	56.81 ± 8.14	53.84 ± 5.40
**4**	56.38 ± 2.08	56.28 ± 8.47	56.61 ± 4.40
**6**	59.18 ± 4.60	61.62 ± 3.92	71.22 ± 4.85
**7**	55.32 ± 8.44	57.76 ± 7.87	55.59 ± 5.02
**8**	49.52 ± 5.52	54.37 ± 2.50	54.60 ± 1.72
**9**	54.90 ± 6.16	56.64 ± 7.86	52.72 ± 3.27
**10**	54.34 ± 4.59	53.35 ± 2.82	52.36 ± 6.59
**11**	55.00 ± 7.68	55.29 ± 4.70	55.95 ± 3.83
**12**	87.87 ± 5.05	71.61 ± 4.43	68.58 ± 6.28
**14**	53.15 ± 5.69	54.86 ± 4.25	54.17 ± 5.64
**15**	58.59 ± 7.76	58.75 ± 7.76	55.62 ± 6.33

Table S 13 Molecular docking results of representative compounds with Keap1-Nrf2 (PDB: 4L7B), BACE1 (PDB: 1M4H), and APOE4 (PDB: 1B68).

Ligand	Protein	Binding energy (kcal/mol)	Number of hydrogen bonds (total/drug-enzyme)	Amino acid residue (H-bond distance Å)
**1**	4L7B	-8.9	15/12	GLN-563 (2.4, 2.4); ARG-326 (2.5, 2.2, 2.1); VAL-420 (2.2); VAL-467 (2.1); VAL-514 (2.1); VAL-512 (2.2); VAL-463 (2.3); VAL-604 (2.3, 2.5)
	1M4H	-10.0	9/7	THR-275 (2.7, 2.3); TRP-277 (2.3); ASP-363 (2.9); SER-58 (2.7, 2.4); HIS-362 (2.6)
	1B68	-7.7	17/13	ARG-142 (2.3, 2.3, 2.0); ARG-145 (2.7, 2.2, 1.8); ARG-38 (2.2, 2.1); ARG-150 (2.3); LEU-30 (2.9); GLU-27 (2.2); GLN-156 (2.3, 2.0)
**6**	4L7B	-9.2	21/14	ARG-326 (2.5, 1.9); HIS-516 (2.9, 2.6, 2.1); ASN-517 (2.4); ASN-469 (2.2); ARG-470 (2.2); VAL-369 (2.4); ILE-416 (2.8); LEU-365 (2.5); ALA-510 (2.1); LEU-557 (2.7, 2.1)
	1M4H	-10.3	12/6	THR-275 (2.4); TYR-320 (2.1); TRP-277 (2.7); ARG-64 (2.4, 1.9); GLY-66 (2.7)
	1B68	-8.5	12/11	ARG-142 (2.4, 2.2); ARG-38 (2.1); ARG-145 (2.4, 2.2); LYS-146 (2.7, 1.9); ARG-150 (2.7, 2.6); GLN-156 (2.4, 2.1)
Trolox	4L7B	-7.5	2/2	VAL-512 (2.6); LEU-557 (2.5)
	1M4H	-7.3	2/2	TYR-320 (2.1); THR-275 (2.5)
	1B68	-5.7	/	/
